# Non-canonical fungal G-protein coupled receptors promote Fusarium head blight on wheat

**DOI:** 10.1371/journal.ppat.1007666

**Published:** 2019-04-01

**Authors:** Tess Dilks, Kirstie Halsey, Rebecca P. De Vos, Kim E. Hammond-Kosack, Neil Andrew Brown

**Affiliations:** 1 Biointeractions and Crop Protection, Rothamsted Research, Hertfordshire, United Kingdom; 2 Computational and Analytical Sciences, Rothamsted Research, Hertfordshire, United Kingdom; 3 Department of Biology & Biochemistry, University of Bath, Claverton Down, Bath, United Kingdom; Institute of Microbiology, CHINA

## Abstract

Fusarium Head Blight (FHB) is the number one floral disease of cereals and poses a serious health hazard by contaminating grain with the harmful mycotoxin deoxynivalenol (DON). Fungi adapt to fluctuations in their environment, coordinating development and metabolism accordingly. G-protein coupled receptors (GPCRs) communicate changes in the environment to intracellular G-proteins that direct the appropriate biological response, suggesting that fungal GPCR signalling may be key to virulence. Here we describe the expansion of non-classical GPCRs in the FHB causing pathogen, *Fusarium graminearum*, and show that class X receptors are highly expressed during wheat infection. We identify class X receptors that are required for FHB disease on wheat, and show that the absence of a GPCR can cause an enhanced host response that restricts the progression of infection. Specific receptor sub-domains are required for virulence. These non-classical receptors physically interact with intracellular G-proteins and are therefore bona fide GPCRs. Disrupting a class X receptor is shown to dysregulate the transcriptional coordination of virulence traits during infection. This amounts to enhanced wheat defensive responses, including chitinase and plant cell wall biosynthesis, resulting in apoplastic and vascular occlusions that impede infection. Our results show that GPCR signalling is important to FHB disease establishment.

## Author summary

Fusarium Head Blight (FHB) is the number one floral disease of cereals and poses a serious health hazard by contaminating grain with harmful mycotoxins. *Fusarium graminearum* adapts to the host plant environment, coordinating fungal development, metabolism and virulence. Here we show that non-classical G-protein coupled receptors (GPCRs) contribute to FHB disease on wheat, promoting symptomless infection through their regulation of fungal membrane, mycotoxin and secreted protein biosynthesis. Disruption of GPCR host sensing activated an enhanced wheat defensive response to infection. This amounts to increased chitinase and plant cell wall biosynthesis, resulting in apoplastic and vascular occlusions that impede the progression of symptomless infection. These non-classical receptors were confirmed to be bona fide G-protein interactors, and specific receptors domains were required for virulence. Our results show that GPCR signalling is important to FHB disease establishment. The discovery of fungal GPCRs and specific extracellular domains that influence sterol membrane and mycotoxin biosynthesis, while contributing to virulence, opens new avenues for biotechnology to minimise diseases in crop species.

## Introduction

Wheat is prone to FHB disease when warm moist weather coincides with *Fusarium graminearum* spores arriving during crop anthesis. Germinating fungal spores produce hyphae that invade the inner surface of the floral tissues, with or without the production of complex infection structures [1]. The trichothecene (*TRI*) genes responsible for deoxynivalenol (DON) biosynthesis are highly expressed within these infection structures [1]. Once within the plant, invasive hyphae grow throughout the wheat head. Infection first spreads via symptomless colonisation of the apoplast, between live plant cells [2], during which the *TRI* genes are again highly expressed [3,4]. After the establishment of infection, the pathogen promotes the colonisation and deconstruction of dead plant cells, causing the visible bleached appearance characteristic of FHB disease [2]. DON is required for the establishment and progression of wheat infection. In DON’s absence, wheat cells mounts a defensive response that confines infection [5]. Other biologically active secreted molecules contribute to virulence [6], including the Fgl1 lipase that inhibits callose deposition and facilitates vasculature colonisation [7,8], the iron scavenging triacetyl fusarinine C siderophore [9–11], and carbohydrate-active enzymes that deconstruct plant cell walls and induce host cell death [2,4,12–15]. These virulence traits are spatially and temporally coordinated during infection [4], suggesting that the pathogen is responding to micro-environmental changes whilst inside the host.

G-protein signalling regulates fungal development, DON production and virulence in *F*. *graminearum*, where five G-proteins and seven RGS (repressors of G-protein signalling) differentially regulate vegetative growth, sexual reproduction, cell wall composition, DON production and virulence [16,17]. Components of the downstream cAMP-Protein Kinase A (PKA) pathway, including the adenylate cyclase and the major PKA catalytic subunit, have a broad impact on growth, sporulation, DON production, the formation of infection structures and virulence [18,19]. Further downstream, the terminal Map1 mitogen-activated kinase (MAPK) of the pheromone responsive/filamentous growth pathway regulates growth, sexual development, hydrolytic enzyme secretion (including Fgl1) and virulence [20–22]. The Map1 pathway-specific FgSte12 transcription factor also influences hydrolytic enzyme secretion and virulence [23]. The cell wall integrity MAPK pathway influences growth, sexual development, DON production, and virulence, whilst the osmotic stress response MAPK pathway impacts upon sexual development, DON production, and virulence [24,25]. The well-characterised contributions of the G-protein, cAMP-PKA and MAPK pathways to virulence suggest an important role for unknown upstream GPCR signalling events in sensing host-derived ligands and promoting infection.

Here we describe the expansion of non-classical GPCRs in *F*. *graminearum*, and show that class X receptors are highly expressed during wheat infection. We identify class X receptors that are required for FHB disease on wheat. These non-classical receptors are shown to physically interact with intracellular G-proteins. Disrupting a class X receptor is shown to dysregulate the transcriptional coordination of virulence traits during infection. This amounts to enhanced wheat defensive responses, including chitinase and plant cell wall biosynthesis, resulting in apoplastic and vascular occlusions that impede rachis infection. Our results show that GPCR signalling is important to FHB disease establishment.

## Results

### Expansion of non-classical GPCRs in *Fusarium graminearum*

**Fig 1:**
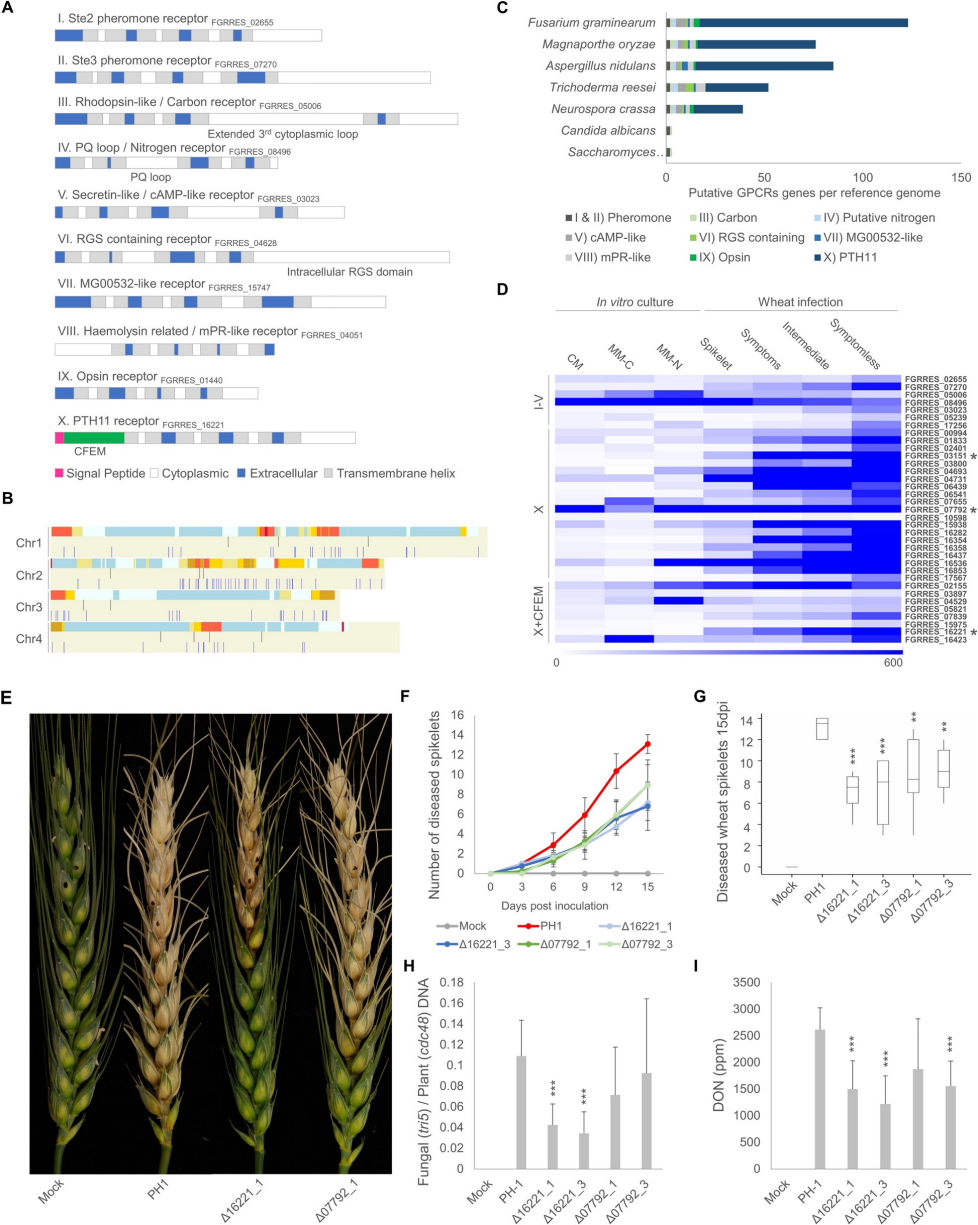
Putative *Fusarium graminearum* GPCRs expression patterns during infection and contribution to virulence on wheat. **A)** Classification of the ten putative GPCR classes in *F*. *graminearum*, according to structural similarities [27]. Schematics depict the putative protein structures and distinct characteristics of each class. **B)** Distribution of putative classical (black) and non-classical (blue) GPCR-encoding genes across the four *F*. *graminearum* chromosomes alongside a chromosomal recombination frequency heatmap: Red high level–blue low level recombination. Displayed using OmniMap [50]. **C)** Expansion of class X receptors in *F*. *graminearum* in comparison to other ascomycete fungi, including model non-pathogenic and pathogenic yeasts *Saccharomyces cerevisiae* and *Candida albicans*, plus saprophytic (*Aspergillus nidulans*, *Neurospora crassa* and *Trichoderma reesei*) and plant pathogenic (*Magnaporthae oryzae*) fungi from the subphylum Pezizomycotina [26,27]. **D)** Affymetrix expression heatmap for selected *F*. *graminearum* classical (classes I-V) and non-classical (class X with or without a CFEM domain) receptors in axenic culture or during wheat infection [4]. Axenic culture represented by complete media (CM), carbon (MM-C) and nitrogen (MM-N) starvation. Wheat infection represents the distinct phases of infection at 7 days post infection (dpi), namely symptomless rachis infection, intermediate rachis infection, symptomatic rachis and symptomatic spikelet infection. * denotes GPCR encoding genes with either the highest absolute level, or fold change, in expression during infection. **E)** Identification of *F*. *graminearum* GPCRs that contribute to virulence. Presented are mutants lacking either FGRRES_16221 or FGRRES_07792, compared to the parental PH-1 strain and the mock non-infected controls at 15 dpi. **F)** Disease progression throughout the 15 day time course. **G)** Boxplot showing the number of diseased spikelets at 15 days. **H)** Quantification of fungal burden, within wheat at 15 dpi, presented as the ratio of *F*. *graminearum tri5* to wheat *cdc48* DNA. **I)** DON mycotoxin contamination within wheat heads at 15 dpi. G-I) *** = *p*<0.001, ** = *p* <0.01.

The *F*. *graminearum* genome encodes 123 putative GPCRs containing 7- transmembrane domains [26]. Based on sequence homology and structural similarity, the 123 GPCRs can be sub-divided into 10 classes (**Fig 1A**) [26,27]. In *F*. *graminearum* this includes the class I and II sex pheromone receptors, a class III carbon receptor, two class IV nitrogen receptors, five class V cAMP-like receptors, a class VI RGS domain-containing receptor, a class VII MG00532-like receptor, two class VIII mPR-like receptors, three class IX opsin receptors and 106 class X PTH11-like receptors. Similarly, 61.5% of classical (classes I-V and IX) and 66.3% of non-classical (classes VI-VIII and X) GPCRs resided in chromosomal regions associated with high frequency recombination and virulence (**Fig 1B**) [28]. The number of putative GPCRs in *F*. *graminearum* is expanded in comparison to other model non-pathogenic and pathogenic ascomycete yeasts and filamentous fungi (**Fig 1C**), primarily due to the increased number of class X receptors. No tandem duplications of PTH11-like receptors are reported [29]. These PTH11-like receptors are restricted to the filamentous Pezizomycotina subphylum of Ascomycota, and are more prevalent in fungi which interact with live or dead plants [27,29]. Among the 106 *F*. *graminearum* PTH11-like receptors, 8 possess an extracellular cysteine-rich CFEM domain (PF05730), common in fungal cell membrane proteins [30]. The expansion of non-classical receptors in *F*. *graminearum* and their location within genomic evolutionary hotspots, suggests that these receptors may be important for virulence.

### Class X receptors are transcriptionally induced during wheat infection

Quantifying the transcriptional regulation of the *F*. *graminearum* GPCR encoding genes during either axenic culture or the progression of wheat infection [4] revealed that the classical class I-V receptors were not highly expressed during plant infection. In contrast the non-classical class X receptors were highly expressed, particularly during the initial phase of symptomless infection (**Fig 1D**). The FGRRES_03151 receptor had the highest fold induction (ranging from 80 to 130-fold) throughout wheat infection, while the FGRRES_07792 receptor had the highest absolute level of expression, which represented an 8.4-fold induction, during the later phase of symptomatic infection. However, the CFEM-containing PTH11-like receptor, FGRRES_16221, showed the second highest fold induction (123-fold) particularly during the establishment of symptomless infection (**S1 Fig**). This is reminiscent of the expression of PTH11-like receptors in other fungal phytopathogens and saprophytes during either plant infection or growth on complex plant-derived carbon sources [29]. These transcriptional profiles suggest that non-classical class X receptors may be involved in wheat infection.

### Screening *Fusarium graminearum* GPCR mutants for reduced virulence on wheat

Two independent null mutants were generated for each receptor. This included 7 classical class I-V receptors, 19 class X receptors with the highest fold transcriptional induction, or absolute expression, during wheat infection, and 7 class X PTH11-like receptors that contained the extracellular CFEM domain. The absence of individual class I-V receptors had no impact on virulence. In contrast, mutants of two class X receptors (FGRRES_07792 and FGRRES_16536) and three PTH11-like CFEM-containing class X receptors (FGRRES_16221, FGRRES_02155 and FGRRES_07839) showed reduced FHB causing abilities on wheat (**S2 Fig**), highlighting the potential importance of this receptor class to disease. Two of the receptor mutants that showed reduced virulence were FGRRES_07792, which had the highest absolute level of expression during symptomatic infection, and FGRRES_16221, which had the highest transcriptional induction during symptomless infection. Subsequently these two receptors were selected for further investigation.

### Class X receptors FGRRES_07792 and FGRRES_16221 are involved in virulence, but are not essential for DON production

The progression of FHB symptoms on wheat was monitored for 15 days. Disease progression of the FGRRES_07792 and FGRRES_16221 mutants consistently lagged behind that of the parental PH-1 strain, ultimately reducing the final number of diseased spikelets (**Fig 1E–1G**). The FGRRES_07792 and FGRRES_16221 mutants also showed reduced virulence on the floral tissue of non-host plant *Arabidopsis thaliana* (**S3 Fig**). Reduced wheat disease symptoms correlated with a reduction in fungal burden, which was most pronounced for the FGRRES_16221 mutants (**Fig 1H**). DON was detected despite the reduced disease symptoms produced by the FGRRES_07792 and FGRRES_16221 mutants. DON accumulation was reduced in wheat heads infected by the FGRRES_07792 and FGRRES_16221 mutants (**Fig 1I**). However, normalisation of DON levels according to fungal burden revealed the FGRRES_16221 mutant produced more DON per fungal unit. Therefore, the FGRRES_07792 and FGRRES_16221 receptors are required for full virulence on wheat, but are not essential for DON production.

### Class X receptors FGRRES_07792 and FGRRES_16221 are not required for growth on plant-derived carbon or sexual reproduction

The FGRRES_07792 and FGRRES_16221 receptors did not influence fungal growth on nutrient-rich or nutrient-poor media (**S4A Fig**). Additionally, these receptors did not influence growth on a range of plant-derived carbon sources, including simple saccharides fructose, xylose and cellobiose, plus complex polysaccharides, carboxymethyl-cellulose, inulin, pectin and xylan (**S4B Fig**). This suggests that the absence of these receptors does not significantly alter the pathogen’s capacity to deconstruct and/or utilise complex plant-derived carbon. This contrasts the function of PTH11-like receptors in saprophytes *N*. *crassa* and *T*. *reesei*, which contribute to lignocellulose utilisation [31,32]. The FGRRES_07792 and FGRRES_16221 receptors did not influence the pathogen’s ability to undergo homothallic perithecial development and the subsequent release of ascospores (**S4C Fig**). The absence of *in vitro* growth and sexual development phenotypes suggests that the FGRRES_07792 and FGRRES_16221 receptors primarily function during interactions with the wheat head, in accordance with their elevated plant infection expression profiles.

### Class X receptors FGRRES_07792 and FGRRES_16221 show limited phylogenetic conservation in fungi

**Fig 2:**
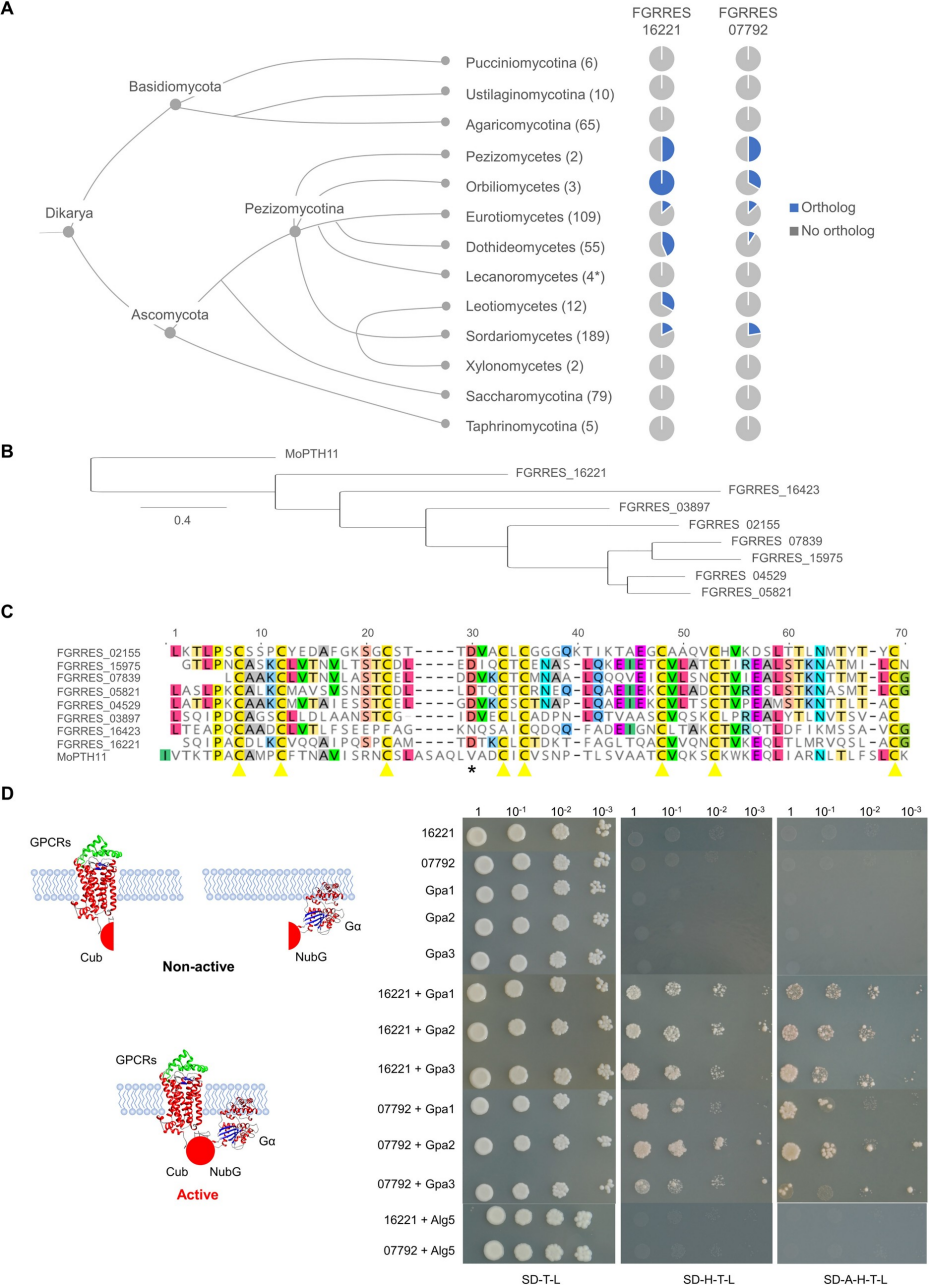
The discontinuous distribution of bona fide *Fusarium graminearum* non-classical G-protein coupled receptors. **A)** Phylogenetics of putative *F*. *graminearum* GPCR encoding genes. The identification of FGRRES_16221 and FGRRES_07792 orthologues shows a discontinuous distribution throughout the fungal tree of life. Diagrammatic representation of the fungal tree of life (not indicative of evolutionary time) adapted from the JGI MycoCosm [52]. Pie charts show the number of species genomes within each taxonomic class with at least one FGRRES_16221 or FGRRES_07792 orthologue as predicted by PhytoPath [51]. The number of species genomes present in each group analysed is shown in brackets. * No Lecanoromycete genomes were represented in the PhytoPath dataset. Therefore, BlastP analyses using the mature FGRRES_07792 and FGRRES_16221 protein sequences were performed, at the expected (e-value) cut-off thresholds of 1x10^-50^ and 1x10^-100^, on the predicted proteomes of four Lecanoromycetes presented on the JGI Mycocosm portal [52]. **B)** A phylogenetic tree based on the alignment of the eight *F*. *graminearum* class X receptors, which possess the extracellular CFEM domain, with the original fungal class X receptor, PTH11 from *Magnaporthae oryzae*, labelled MoPTH11. **C)** Alignment of the CFEM domain from the eight *F*. *graminearum* class X GPCRs and MoPTH11, showing the conservation of the cysteine residues. * denotes Asp residue conserved in heme-binding CFEM proteins [34]. **D)** Left panel—Schematic depicts the yeast split ubiquitin approach for identification of receptor-G-protein interactions at the cell membrane. Right panel—Assay demonstrates class X non-classical (FGRRES_07792 and FGRRES_16221) receptors specifically interact with multiple Gα-proteins at the cell membrane. Yeast serial dilutions (1:1, 1:10, 1:100, 1:1000) were grown on non-selective SD media lacking tryptophan (T) and leucine (L), plus selective media also lacking histidine (H) and adenine (A). The Alg5 membrane protein is a non-GPCR interacting control.

Orthologues of the FGRRES_07792 and FGRRES_16221 were identified across 436 fungal genomes, representing the 13 different taxonomic classes and subphyla within Dikarya (**Fig 2A**). FGRRES_07792 and FGRRES_16221 orthologues were restricted to Pezizomycotina, excluding the Lecanoromycetes and Xylonomycetes. Additionally, FGRRES_07792 had no orthologues among the Leotiomycetes. Within Pezizomycotina, FGRRES_16221 showed the greater number of orthologues. Among the eight *F*. *graminearum* PTH11-like receptors with a CFEM domain, FGRRES_16221 showed the highest similarity to the founding member of the class X receptors, PTH11 from *M*. *oryzae* [33]. Both FGRRES_16221 and MoPTH11 are predicted to possess N-terminal signal peptides and an extracellular CFEM domain, seven transmembrane domains and a C-terminal cytoplasmic tail. In *F*. *graminearum*, these eight PTH11-like receptors with a CFEM domain showed the conservation of the eight cysteine residues, and seven receptors possessed an aspartic acid residue conserved in heme-binding CFEM proteins that is not present in MoPTH11 [34] (**Fig 2C**).

### Class X receptors FGRRES_07792 and FGRRES_16221 interact with Gα-proteins at the yeast cell membrane

Class X receptors in *F*. *graminearum* are putatively classified as GPCRs [26,27]. The yeast split ubiquitin assay was used to determine if these non-classical receptors were G-protein interactors. The classical Ste2 and Ste3 sex pheromone receptors, plus the non-classical FGRRES_07792 and FGRRES_16221 receptors (bait) were fused with the C-terminal ubiquitin (Cub) fragment and the LexA-VP16 reporter. The three *F*. *graminearum* Gα-proteins (prey: GzGPA1, GzGPA2 and GzGPA3) and the Alg5 membrane protein (non-GPCR interacting protein) were fused with the N-terminal ubiquitin (NubG) fragment. Reconstitution of ubiquitin, as the result of a receptor-Gα-protein interaction at the cell membrane, released the LexA-VP16 reporter to promote growth on selective media. Yeast strains individually carrying receptors or Gα-proteins, or the receptors plus Alg5, did not grow on selective media, excluding the possibility of auto-activation or non-specific interactions. Yeast strains harbouring the classical pheromone receptors, or the non-classical FGRRES_07792 and FGRRES_16221 receptors, in conjunction with any of the three Gα-proteins grew on selective media (**Fig 2D; S5 Fig**). This shows that when overexpressed in a heterologous system, both classical and non-classical receptors have the capacity to interact with multiple Gα-proteins at the cell membrane, and proves that the class X receptors, FGRRES_07792 and FGRRES_16221, are G-protein interactors. However, it remains unknown if that FGRRES_07792 and FGRRES_16221 are activators or repressors of G-protein signalling.

### The FGRRES_16221 CFEM domain and cytoplasmic terminus contribute to receptor function and virulence

**Fig 3:**
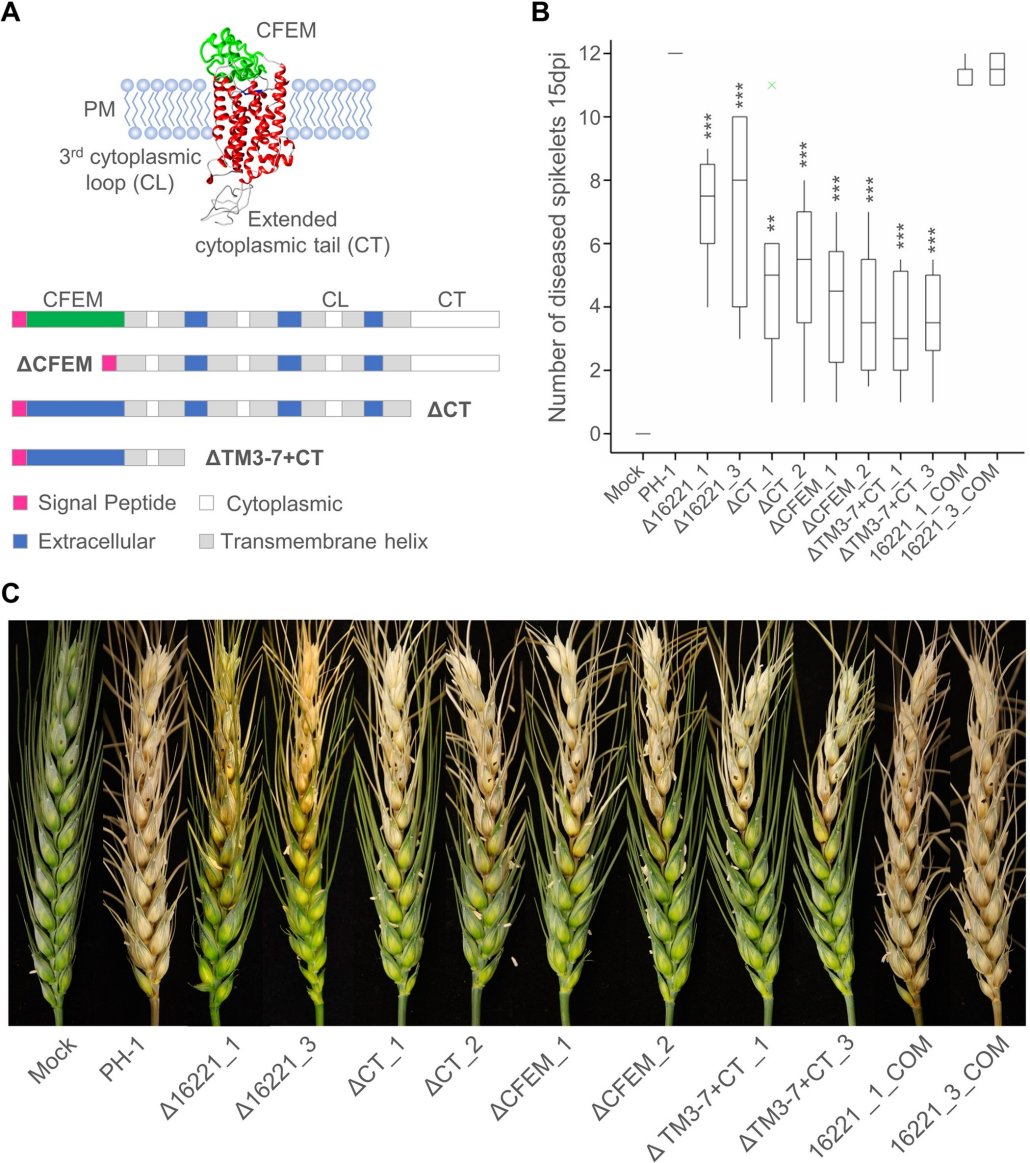
FGRRES_16221 receptor sub-domains are important to virulence. **A)** The putative protein structure of GPCR FGRRES_16221, as predicted by the Phyre2 software [61], showing the presence of a cysteine-rich extracellular CFEM domain, plus the 3^rd^ cytoplasmic loop (CL) and extended cytoplasmic tail (CT) potentially involved in interactions with downstream Gα-proteins. Schematic initially depicts the organisation of the native FGRRES_16221 protein and then shows the three FGRRES_16221 truncations generated. This includes ΔCFEM lacking the CFEM domain, ΔCT lacking the cytoplasmic tail and ΔTM3-7+CT lacking the majority of the protein (containing the 3^rd^ cytoplasmic loop and cytoplasmic tail), leaving the extracellular CFEM domain tethered to the plasma membrane. In addition, the Δ16221_1 and Δ16221_3 mutants were both complemented with the native FGRRES_16221 gene. **B)** A boxplot showing the number of diseased spikelets at 15 days. The three types of FGRRES_16221 truncations all showed reduced disease symptoms on wheat at 15 days post infection (dpi). Truncations had a more severe impact on fungal virulence than the entire FGRRES_16221 gene deletion. Complementation of the Δ16221_1 and Δ16221_3 mutants (labelled—COM) restored fungal virulence to level equivalent to the parental PH-1 strain. *** = *p*<0.001, ** = *p*<0.01. **C)** The appearance of wheat disease symptoms at 15 dpi. Presented are two independent *F*. *graminearum* mutants of the deletion, truncation and complementation of the FGRRES_16221 receptor, compared to the parental PH-1 strain and the mock non-infected controls.

The CFEM domains found in a subset of class X receptors are proposed to function in the recognition of, and adhesion to, the host, while the cytoplasmic carboxyl-terminal domain could interact with G-proteins [30,35]. The PTH11-CFEM domain in *M*. *oryzae* is required for appressorium development and pathogenicity [36]. To elucidate the contribution of specific sub-domains to virulence, *F*. *graminearum* strains harbouring three different types of FGRRES_16221 receptor truncations were generated (**Fig 3A**). These included strains lacking the CFEM domain (ΔCFEM) or the cytoplasmic tail (ΔCT), in addition to a strain possessing the membrane tethered CFEM domain, but lacking the majority of the receptor (**Fig 3A**). The FGRRES_16221 truncations were driven by the native promoter and therefore their transcriptional regulation should be unaffected. All of the FGRRES_16221 truncations caused reduced virulence (**Fig 3B and 3C**). Therefore, both the extracellular CFEM domain and the cytoplasmic tail contribute to receptor function and virulence, while the presence of the CFEM domain alone is not sufficient to cause disease. Interestingly, each truncation had a more severe impact on the establishment of FHB than the absence of the entire receptor. This suggests that the lack of this receptor triggered a compensatory mechanism, which was not activated in the fungal strains carrying receptor truncations. Finally, complementation of the two FGRRES_16221 null mutants with the native FGRRES_16221 gene restored virulence to parental PH-1 levels (**Fig 3B and 3C**). Collectively, this data confirms that FGRRES_16221 and the CFEM domain are required for the establishment of FHB on wheat.

### Class X receptors FGRRES_07792 and FGRRES_16221 are involved in wheat floral colonisation, but not essential for penetration

**Fig 4:**
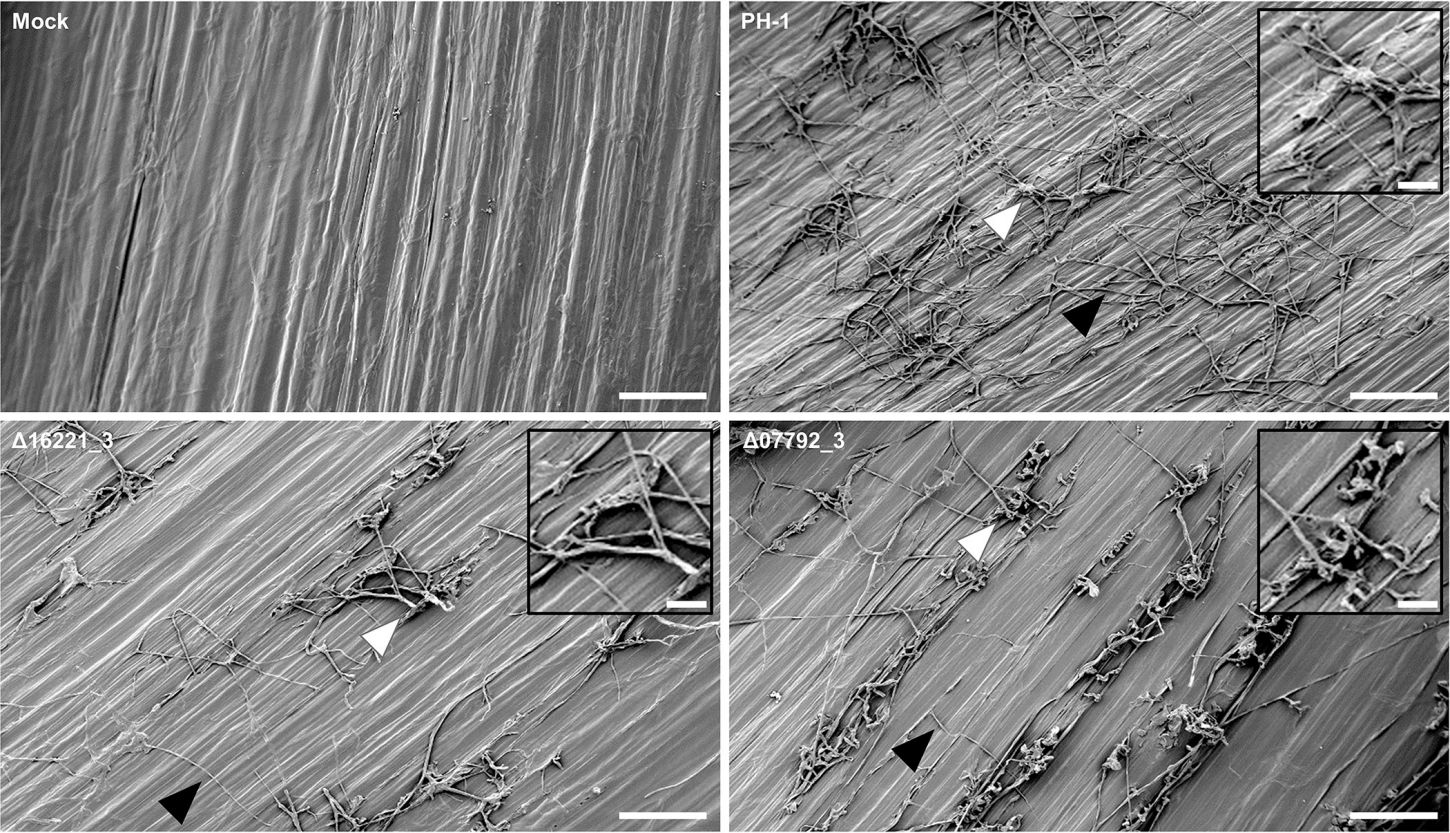
The *Fusarium graminearum* FGRRES_07792 and FGRRES_16221 mutants show reduced floral colonisation, but can penetrate the surface. Presented are scanning electron micrographs of the inner surface of the palea for the non-infected (mock) wheat tissue, the parental PH-1 strain, plus the Δ16221_3 and Δ07792_3 mutants 2 days post infection. Inserts in each panel are higher magnification images of infection structures indicated. White arrow = infection structures. Black arrow = runner hyphae. Bar = 100 μm.

The individual absence of the FGRRES_07792 and FGRRES_16221 receptors caused a reduction in FHB symptoms and fungal burden, but why infection progress was impaired remained unclear. The initial colonisation of the surface of the wheat floret was assessed by scanning electron microscopy at 2-days post infection (dpi). This revealed a reduction in the colonisation of the floral tissues in the absence of either receptor (**Fig 4**). Nonetheless, the mutants lacking individual receptors developed penetration structures on the inner-surface of the palea, resulting in subcuticular infection. However, fewer runner hyphae provided connections between infection structures, and less fungal biomass was observed in comparison to the parental PH-1 strain. This is distinct to the function of PTH11 in *M*. *oryzae*, which is required for appressoria formation and host penetration [33]. Therefore, FGRRES_07792 and FGRRES_16221 both appear to contribute to the effectiveness of initial floral surface colonisation, but not wheat penetration.

### Class X receptor FGRRES_16221 promotes symptomless infection and disease progression

**Fig 5:**
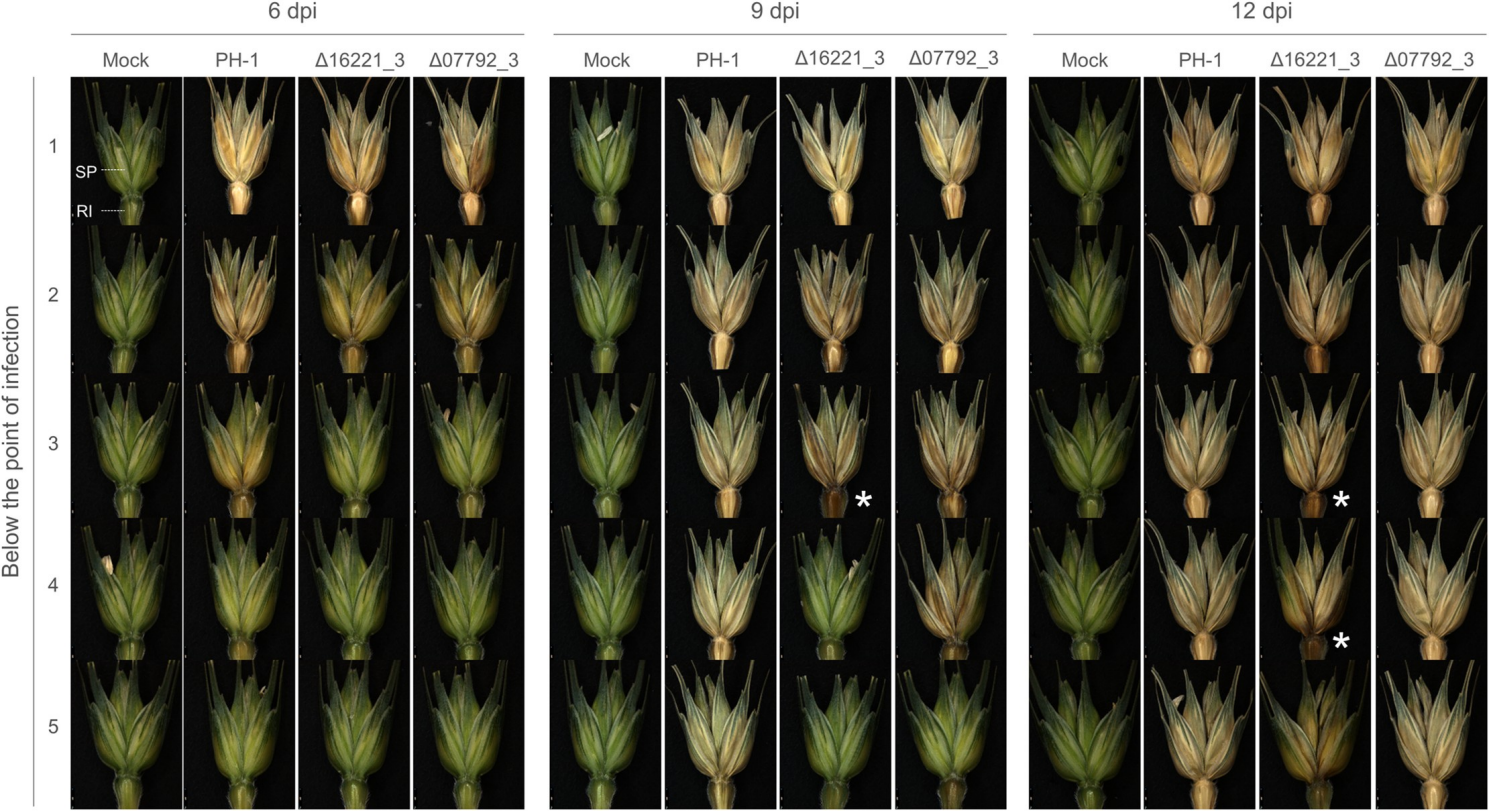
The *Fusarium graminearum* mutants lacking FGRRES_07792 or FGRRES_16221 show a delay in disease progression. Images depict the macro-dissection of individual spikelets (SP) and the associated rachis internode (RI) below the point of inoculation at 6, 9 and 12 days post infection (dpi), for the non-infected (mock) wheat tissue, the parental PH-1 strain, plus the mutant Δ07792_3 and Δ16221_3 strains. * denotes the increased browning of the rachis ahead of the last symptomatic spikelet in the absence of FGRRES_16221.

Assessment of wheat disease progression below the point of infection confirmed that the absence of the FGRRES_16221 receptor caused a delay in the development of FHB symptoms (**Fig 5**). After 9 dpi the rachis ahead of the last visibly diseased spikelet appeared dark brown, a characteristic not observed during PH-1 infection. This is reminiscent of the barrier zones confining infection by the *F*. *graminearum* Δ*tri5* (a DON non-producer) and Δ*fgl1* (an extracellular lipase deficient) strains [5,8]. Importantly, *TRI5* expression is highly induced during, and required for, rachis colonisation [37], while FGL1 inhibits callose formation and promotes rachis colonisation [7].

**Fig 6:**
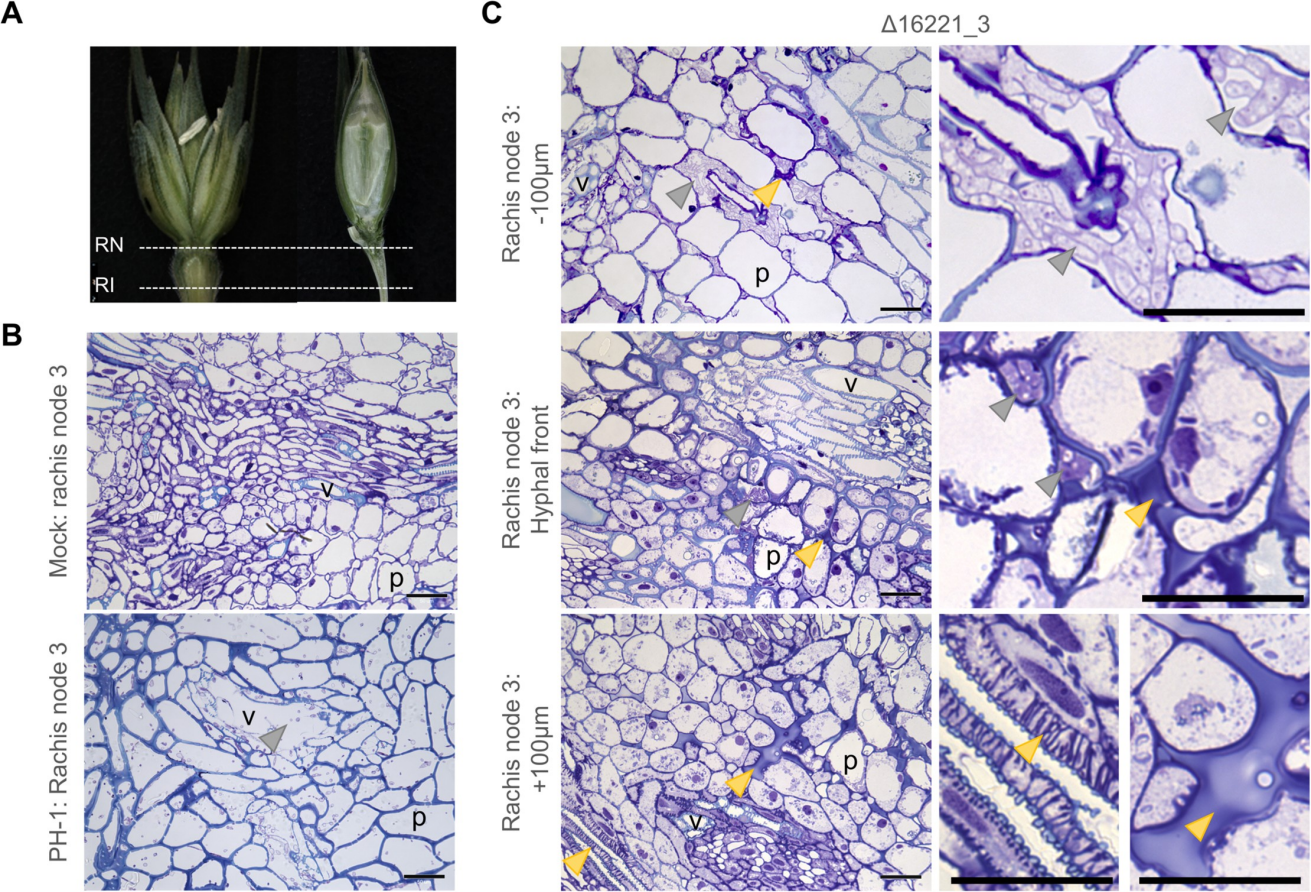
The *Fusarium graminearum* FGRRES_16221 mutant shows defects in establishing symptomless rachis node infection, resulting in apoplastic occlusions. **A)** Images depict non-infected wheat describing the anatomy of the wheat spikelet (SP), rachis node (RN) and internode (RI) tissues. **B)** Light micrographs of transverse sections through the rachis node of the mock and PH-1 infected tissues at 9 days post infection. The healthy mock tissue shows the branching of the vasculature within the rachis node. The fully symptomatic PH-1 infected tissue shows the destruction of the wheat vasculature, the absence of live plant cells and the presence of both inter- and intra-cellular fungal hyphae. Note that PH-1 infection has already advanced multiple rachis nodes ahead. **C)** Light micrographs of transverse sections of the Δ16221_3 infected 3^rd^ rachis node revealing the appearance of infection at the advancing hyphal front, plus ahead (+100 μm) and behind (-100 μm) the hyphal front. Behind the hyphal front, Δ16221_3 accumulates within intercellular spaces between a mixture of live and dead plant cells. At the hyphal front, a limited amount of intercellular hyphae are surrounded by active live plant cells. In advance of the hyphal front, the plant cells are responding to infection resulting in cell fortifications and the occlusion of the intercellular spaces. Arrows: grey = fungal hyphae, yellow = wheat cells responding to infection. Bar = 50 μm. P = parenchyma. V = vasculature.

The rachis node represents a route for fungal infection to progress into the neighbouring spikelets and beyond into the next rachis internode. Within the third rachis node after 9 dpi, the parental PH-1 strain had colonised all wheat cell-types, causing widespread cell death and the destruction of the vasculature (**Fig 6**). No host defensive responses were observed during infection by the parental PH-1 strain. In contrast, in response to infection by the FGRRES_16221 mutant, apoplastic occlusions were abundant throughout the tissues, between live, metabolically active and reinforced wheat cells (**Fig 6**). These host defences appear to restrict the spread of infection, causing the accumulation of hyphae within the apoplast behind the defensive barrier.

**Fig 7:**
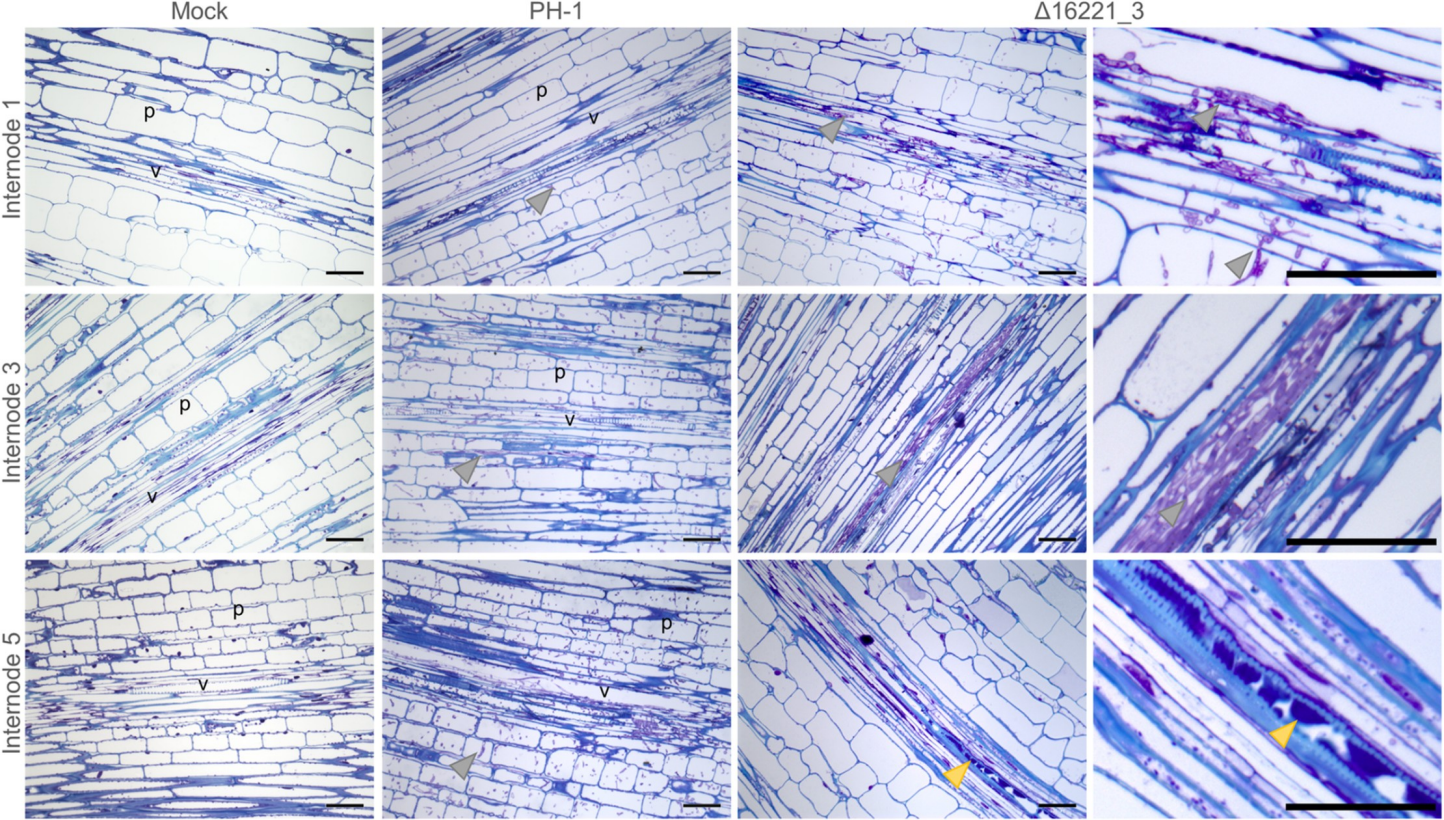
The *Fusarium graminearum* mutant lacking FGRRES_16221 shows delayed rachis internode colonisation and vascular occlusions. The delay in disease progression by the *F*. *graminearum* FGRRES_16221 mutant, in comparison to the parental PH-1 strain at 9 days post infection. Light micrographs of longitudinal sections of the 1^st^, 3^rd^ and 5^th^ rachis internode below the point of infection. In the 1^st^ rachis internode, Δ16221_3 has established both inter- and intra-cellular infection, resulting in plant cell death and the destruction of the vasculature. In the 3^rd^ rachis internode, Δ16221_3 has accumulated within the xylem vessel. This is the only infection beyond the apoplastic occlusion in the 3^rd^ rachis node. In the 5^th^ rachis internode, occlusions in the xylem vessels are observed ahead of infection. In contrast, the fully symptomatic PH-1 infected tissue shows the destruction of the wheat vasculature, the absence of live plant cells, and the presence of both inter- and intra-cellular fungal hyphae throughout the 1^st^, 3^rd^ and 5^th^ rachis internodes. Note rachis infection by the PH-1 strain has already progressed multiple rachis internodes ahead of infection by Δ16221_3. Arrows: grey = fungal hyphae, yellow = occlusions in the xylem vessel. Bar = 100 μm.

Histological examination of sequential rachis internodes, below the respective rachis nodes, also revealed the delayed progression of infection, and reduced fungal burden, by the FGRRES_16221 mutant (**Fig 7**). After 9 dpi, the parental PH-1 strain had inter- and intra-cellularly colonised all wheat cell-types, causing widespread cell death and the destruction of the vasculature. However, infection by the FGRRES_16221 mutant did not progress beyond the 3^rd^ rachis internode, where a mass of fungal hyphae appeared to have accumulated in, and be restricted to, the vasculature (**Fig 7**). This vascular infection represented the only infection beyond the apoplastic occlusions observed in the 3^rd^ rachis node preceding this tissue. Furthermore, ahead of infection by the FGRRES_16221 mutant, occlusions were observed in the xylem vessels of the 5^th^ rachis internode, which is reminiscent of the increased vascular callose depositions in the rachis ahead of Δ*fgl1* infection [7].

### Absence of FGRRES_16221 dysregulates fungal signalling, metabolism and virulence factors

**Fig 8:**
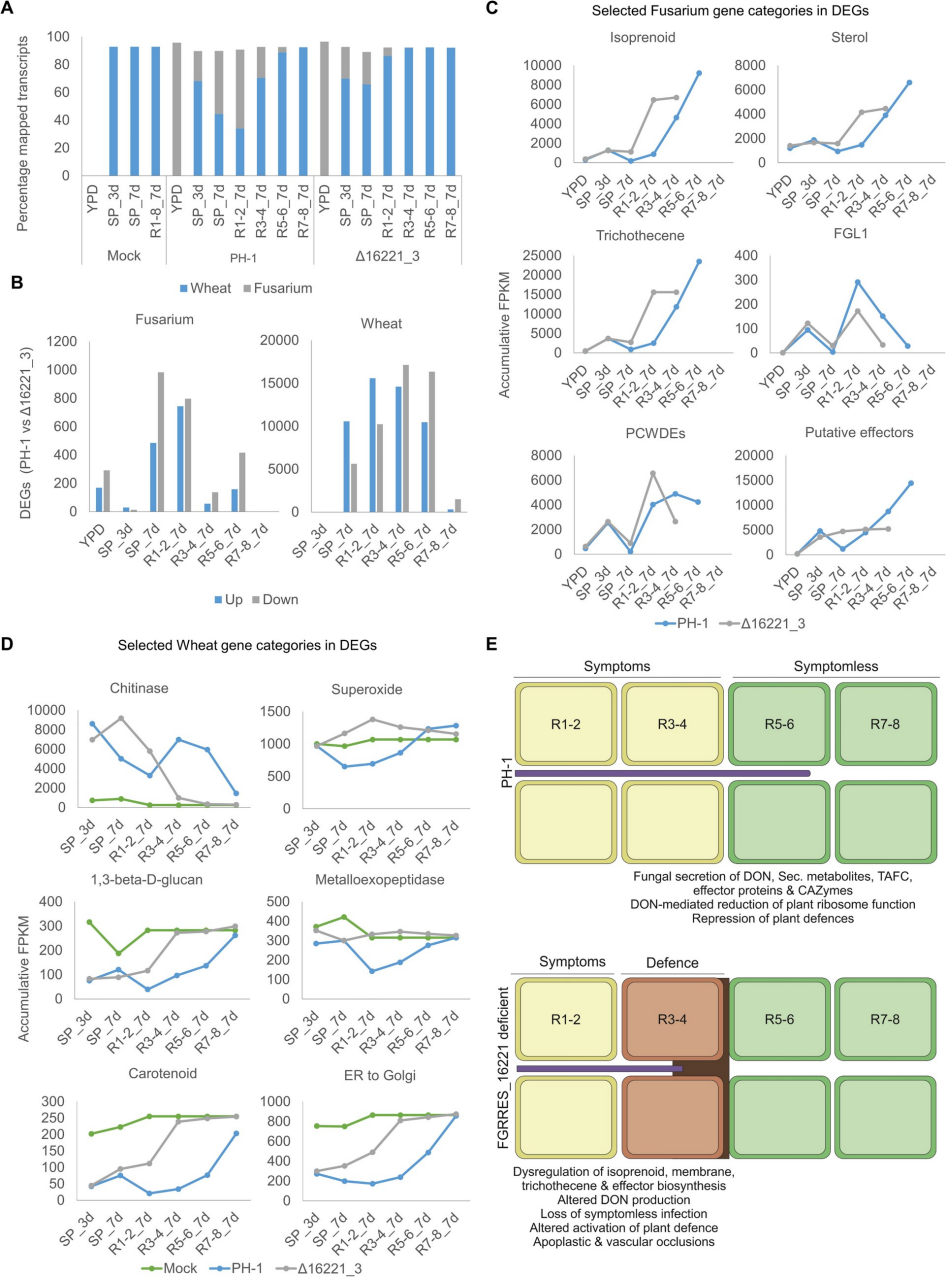
RNA-sequencing reveals the role of FGRRES_16221 in coordinating fungal metabolism and virulence factors to promote infection without activating inhibitory wheat defences. Wheat tissues sampled for RNA-sequencing to evaluate transcriptional differences in the pathogen and host during the infection establishment (spikelet, 3 day post infection; dpi) and the progression of infection (spikelet and sequential rachis internode pairs at 7 dpi). **A)** The ratio of wheat-to-fungal transcripts reflected differences in fungal burden, confirming the reduced progression of infection by the FGRRES_16221 mutant (Δ16221_3). **B)** The number of differentially expressed *F*. *graminearum* and wheat genes (DEGs; FDR <0.05, ±1log2 fold change in expression) identified in pairwise analyses between the parental PH-1 and Δ16221_3 during axenic culture and wheat infection. Overrepresented gene ontologies (GO) were identified within the DEGs and specific gene categories associated with pathogenesis (**S1–S3 Files**). Accumulative FPKM expression values for selected gene categories from *F*. *graminearum* (**C**) and wheat (**D**). Note absolute FPKM values presented for single *FGL1* gene. Legend: YPD = axenic culture in YPD, SP_3d = spikelet 3 dpi, SP_7d = spikelet 7 dpi, R1-8_7d = pooled pairs of rachis internodes below inoculated spikelet at 7 dpi. **E**) Schematic representation of wheat infection by the parental PH-1 strain and the dysregulation of virulence mechanisms in the absence of FGRRES_16221. Green = live wheat cells. Yellow = Dead wheat cells. Brown = Wheat defence response including cell reinforcements, plus apoplastic and vascular occlusions. Purple = Invading *F*. *graminearum* hyphae.

RNA-sequencing was used to evaluate transcriptional differences between the *F*. *graminearum* parental PH-1 and FGRRES_16221 mutant (Δ16221_3) strains in axenic culture (72 h in yeast peptone dextrose; YPD) and during the establishment and progression of wheat infection at 3 and 7 dpi. The inoculated wheat spikelets (SP) and sequential rachis segments below the point of inoculation were harvested (**S9 Fig**). Pairs of rachis segments, which were phenotypically similar in the parental PH-1 infection, were combined, representing the transcriptionally distinct fully symptomatic (R1-2), intermediate (R3-4) and symptomless (R5-6) infection phases [2,4]. The ratio of wheat-to-fungal transcripts was used as a measure of differences in fungal burden, confirming the reduced progression of infection by the FGRRES_16221 mutant (Δ16221_3) (**Fig 8A**). Pairwise analyses between PH-1 and Δ16221_3 infected wheat identified differentially expressed genes (DEGs), overrepresented gene ontologies (GO terms), and the modulation of genes specifically involved in G-protein signalling and virulence.

In *F*. *graminearum*, few DEGs and no overrepresented GO terms were identified during axenic culture, or the early establishment of infection at 3dpi, supporting the concept that FGRRESS_16221 functions during the progression of infection. At 7 dpi significantly more DEGs were identified in the spikelet and first rachis internodes (**Fig 8B; S1 File**). Genes with higher expression in Δ16221_3 corresponded to an overrepresentation of the isoprenoid pathway and downstream virulence traits, including trichothecene and sterol biosynthesis both known to be important to virulence [5,38] (**Fig 8C**). DEGs resided within 65 of the 68 predicted secondary metabolite gene clusters [39]. The expression of many clusters, including those required for DON, remained high in Δ16221_3 behind the infection front, which contrasted the initially higher but then sharp decline in expression observed in PH-1 (**S6 Fig**). This was reflected in the elevated DON accumulation in wheat per fungal unit. Siderophore biosynthesis, which also requires intermediates of the isoprenoid pathway and contributes to virulence [10], showed altered expression during infection. Accordingly, the absence of FGRRES_16221 impacted on fungal growth in the presence of the iron chelator 2–2’-dipyridyl and therefore fungal iron homeostasis (**S7 Fig**). This implies that FGRRES_16221, which contains the putative iron-binding CFEM domain motif, may influence the biosynthesis of toxic and non-toxic secondary metabolites involved in virulence.

The expression of 41 putative secreted effectors [12,40] was altered in Δ16221_3, including the *FGL1* lipase, which inhibits host callose formation to promote infection [7] (**Fig 8C**). Accordingly, in the absence of FGRRES_16221, lipase secretion was reduced when grown on wheat germ oil (**S8 Fig**). This suggests that FGRRES_16221 influences lipase secretion and wheat lipid utilisation. Plant cell wall degrading xylanases and galactosidases showed reduced expression at the Δ16221_3 infection front, but ultimately resulted in elevated expression. Finally, during the progression of spikelet infection from 3 and 7 dpi, PH-1 modulated the expression of 3734 genes, while Δ16221_3 only altered the expression of 363 genes. Therefore, in the absence of FGRRES_16221 the transcription of known and putative virulence factors is dysregulated and fails to reflect the correct transition from symptomless-to-symptomatic infection as observed in the parental PH-1 strain. However, it remains unknown whether FGRRES_16221-mediated G-protein signalling directly, or indirectly, influences the regulation of these metabolite or proteinaceous virulence factors.

Fungal GPCRs do not commonly directly regulate the transcription of G-protein, cAMP-PKA and MAPK signalling components. However, the indirect impact of the absence of FGRRES_16221 on the expression of genes involved in potential down-stream signalling pathways were inspected. This included other GPCRs, G-proteins, cAMP-PKA and MAPK signalling. In the absence of FGRRES_16221, other class X GPCRs, the Gα and β-proteins, GzGpa2 and GzGpb1, RGS (repressors of G-protein signalling), plus the filamentous growth Map1 MAPK and associated Ste12 transcription factor were differentially regulated in comparison to those in the parental PH-1 strain. The differential *MAP1* and *STE12* expression patterns were reminiscent of the differential regulation of trichothecene and sterol biosynthetic genes during Δ16221_3 infections of wheat (**S2 File**). This suggests that the absence of FGRRES_16221 may have an indirect influence on the transcriptional regulation of components of the G-protein signalling and the Map1-Ste12 pathways during wheat infection, pathways known to regulate invasive growth, plus DON and Fgl1 production [5,41,42]. Additionally, in Δ16221_3 there was an up-regulation of components of the cell wall stress MAPK pathway in the 1^st^ rachis internode during infection, reflecting the increased exposure of Δ16221_3 to an enhanced plant defensive response, as observed in the histological examination of rachis infection (**Figs 6 and 7**).

### Absence of FGRRES_16221 permits wheat to mount an enhanced defensive response

The previously described RNA-sequencing study simultaneously permitted the evaluation of the wheat host transcriptional response to infection by the *F*. *graminearum* parental PH-1 and FGRRES_16221 mutant (Δ16221_3) strains at 3 and 7 dpi. In wheat, limited differences were observed during the establishment of spikelet infection at 3 dpi or in the non-infected rachis ahead of infection at 7dpi. However, significant transcriptional difference occurred in the spikelet and infected rachis at 7 dpi (**Fig 8B; S3 File**). Overrepresented GO terms among these wheat DEGs included the elevated expression of genes involved in the response to fungal infection, superoxide metabolism and chitinases (**Fig 8D**), representing the enhanced immune response to Δ16221_3 infection. In contrast, PH-1 infection showed a greater capacity to repress the expression of genes involved in plant cell wall polysaccharide, carotenoid and metalloexopeptidase biosynthesis, in addition to ER-to-golgi transport (**Fig 8D**), processes central to plant defence. Collectively, this reflects wheat’s enhanced defensive response to Δ16221_3 infection, resulting in the occlusions of the apoplast and vasculature that impede rachis infection, potentially caused by the defective regulation of fungal virulence traits in the absence of the FGRRES_16221 receptor (**Fig 8E**).

## Discussion

The expansion of class X receptors in plant interacting Pezizomycota [27], and the identification of multiple class X receptors that contribute to FHB, reflects their function in pathogenesis. The first functionally characterised class X receptor, PTH11 in *M*. *oryzae*, senses hydrophobic plant surfaces, regulating appressoria development and host penetration [33]. FGRRES_16621-mediated signalling however, contributes to floral colonisation, but is not essential for host penetration. FGRRES_16621 is highly expressed at the advancing infection front, promoting the establishment of symptomless infection without activating host defences. Infection in the absence of FGRRES_16221 leads to rachis browning, elevated antifungal chitinases and plant polysaccharide biosynthetic gene expression, plus the appearance of apoplastic and vascular occlusions behind which intercellular hyphae accumulate. This shows the importance of the rachis to host defences and the outcome of *F*. *graminearum* infection.

An inability to secrete DON results in a floral lesion surrounded by a brown halo that confines infection to the spikelet [5]. Whereas the disruption of Fgl1 lipase secretion results in browning of the rachis and vascular callose depositions that impede infection [7,8]. This is despite the Fgl1 mutant exhibiting enhanced DON production [40]. Therefore, within the rachis, where *TRI* gene expression is at its highest [37], the secretion of DON and the Fgl1 effector are both required for infection to progress throughout the wheat head [5,7]. Additionally, the disruption of the capacity to secrete iron scavenging siderophores, triacetyl fusarinine C and malonichrome, increased sensitivity to reactive oxygen species and iron-depletion, stresses encountered during plant infection, resulting in reduced virulence on wheat [9–11].

The absence of FGRRES_16221 also caused elevated DON production and the browning of the rachis during wheat infection, while lipase secretion during growth on wheat germ oil was reduced, reminiscent of the Fgl1 mutant, and sensitivity to iron-depletion was increased. FGRRES_16221 disruption impacted on fungal transcription during the progression of rachis infection, resulting in the dysregulation of DON, siderophore and Fgl1 biosynthesis, in addition to other secondary metabolites and effector proteins putatively associated with virulence. How FGRRES_16221-mediated signalling directly, or indirectly, influences the biosynthesis of these secreted virulence traits remains unknown. Nonetheless, this disruption impedes the progression of rachis infection, due to the loss of symptomless infection and the activation of an enhanced host response. The elevated transcriptional induction of the isoprenoid pathway, ergosterol biosynthesis and cell wall stress signalling in the FGRRES_16221 deficient hyphae accumulated behind apoplastic and vascular occlusions, may reflect a fungal response to the hostile environment, resulting in the observed increase in DON production.

Extracellular CFEM domains are present in a subset of class X receptors and are proposed to function in host recognition, signal transduction, adhesion and virulence [30,33,36,43]. In *F*. *graminearum*, the CFEM domain of FGRRES_16221 contributed to the establishment of symptomless infection. The CFEM domain of PTH11 in *M*. *oryzae* is also required for receptor function, appressoria formation, virulence and redox regulation [36]. The commensal and opportunistic human pathogen *Candida albicans* encodes three heme-binding secreted, cell wall and cell envelope localised CFEM proteins, namely, Rbt5, Pga7 and Csa2, which are highly expressed in low iron environments, including host infection [34,44,45]. The novel helical-basket fold consisting of six α-helices is stabilized by disulphide bonds formed from the eight cysteine residues. The Asp80, which residue serves as the axial ligand conferring oxidation-specific Fe^3+^ heme binding properties of the *C*. *albicans* proteins [34], is absent from PTH11 in *M*. *oryzae*. These structural traits are conserved in the CFEM domain of the FGRRES_16221 receptor. Therefore, during *F*. *graminearum* infections of wheat the environmental iron and oxidation-state of the apoplast may play a role in the activation of FGRRES_16221-mediated signalling and the coordination of virulence. The discovery of a fungal GPCR and specific extracellular domains that influence sterol membrane and mycotoxin biosynthesis, while contributing to virulence, opens new avenues for biotechnology to minimise diseases in crop species.

## Materials and methods

### Fungal propagation

*Fusarium graminearum* strains were cultured and conidia prepared as previously described [2]. Fungal strains were grown for 3 and 5 days on nutrient-rich potato dextrose agar (PDA), nutrient-poor synthetic nutrient agar (SNA; 0.1% KH_2_PO4, 0.1% KNO_3_, 0.1% MgSO_4_·7H2O, 0.05% KCL, 0.02% glucose, 0.02% sucrose and 2% agar) and SNA minus carbon supplemented with 1% of an alternative carbon source (i.e. fructose, xylose, cellobiose, CMC, inulin, pectin or xylan). Fungal strains were grown on SNA in the presence of the iron chelator 2–2’-dipyridyl, at concentrations ranging from 50–200 μM, for 3 days. Fungal growth was photographed on a Nikon D40X under natural light. The ability to undergo homothallic perithecial development and the release of ascospores was assessed on carrot agar as previously described [20]. Perithecia were imaged on a Lecia MZFL11 stereomicroscope under bright field light.

### Plant host and non-host floral infections

The susceptible spring wheat (*Triticum aestivum*) cultivar, Bobwhite, was grown as previously described [2]. At anthesis, 5 μl droplets of 5x10^4^ conidia were placed in the floral cavity of the outer florets of the 5^th^ and 6^th^ spikelet from the top of the wheat head. The inoculated plants were kept at high humidity for 48 h, of which the first 24 h were dark [2]. Disease symptoms were scored by counting the number of symptomatic spikelets below the point of inoculation every three days until day 15. *Arabidopsis thaliana* ecotype Landsberg *erecta* (L*er*-0) was grown and floral spray inoculated, when at least two open flowers and no more than three siliques present on their primary bolts, and scored using the Fusarium Disease Index as previously described [46].

### Fungal genetic manipulation

Split marker-mediated transformation targeted fungal genes for replacement with the hygromycin, or geneticin, selectable markers by homologous recombination [47]. The NEBuilder Assembly tool was used for primer design (**S1 Table**). The Gibson Assembly Cloning kits (New England Biolabs) were used to amplify the respective DNA sequences, which were ligated into the pGEMT-easy plasmid (Promega) and transformed into 5α Competent *Escherichia coli*. For each targeted gene replacement, two plasmids contained either the 5’ flanking region plus the first hygromycin/geneticin fragment, or the second hygromycin/geneticin fragment plus the 3’ flanking region. For FGRRES_16221 truncations, the two plasmids contained either the 5’ flanking region plus first hygromycin fragment, or the second hygromycin fragment plus the truncated FGRRES_16221 gene (the CFEM: 75–370 nt, CT: 1380–1620 nt and TM3-7+CT: 730–1620 nt) and the 3’ flanking region. Plasmids were recovered using GenElute Plasmid Miniprep Kit (Sigma Aldrich). Transformation cassettes were PCR amplified using HotStarTaq DNA Polymerase (Qiagen) and purified by phenol(25):chloroform(24):isoamyl-alcohol(1) (Sigma Aldrich) precipitation. The *F*. *graminearum* parental PH-1, Δ16221_1 and Δ16221_3 strains were polyethyleneglycol (PEG)-mediated protoplast transformed as previously described [45]. Positive gene disruption transformants were selected with hygromycin (75 μg/ml; Calbiochem), and complemented transformants were selected with geneticin (75 μg/ml; Sigma Aldrich). Mycelia was collected from 2-day old yeast extract peptone dextrose (YPD) cultures grown at 25°C, 180 rpm. Fungal DNA was extracted as previously described [48]. Homologous integration was confirmed by PCR using external 5’ and 3’ primers paired with primers internal to the split marker fragments. Two or three independent transformants were selected for biological evaluation.

### Fungal burden and DON quantification

Wheat heads were frozen and freeze dried at 15 dpi, then ground into a powder under liquid nitrogen. A 0.1 g aliquot was removed and total DNA extracted as previously described [48]. Fungal and plant DNA within the sample was determined by absolute qPCR quantification, against a standard curve of genomic DNA of known concentration, using Sybr Green (Sigma Aldrich) with the *F*. *graminearum* specific *TRI5* (5’-GATGAGCGGAAGCATTTCC-3’ and 5’-CGCTCATCGTCGAATTC-3’) and the wheat Cdc48 (5’-GTCCTCCTGGCTGTGGTAAAAC-3’ and 5’-AGCAGCTCAGGTCCCTTGATAC) gene primers. Thermocycle parameters were [95°C, 3 min; (95°C 30 sec, 62°C, 30 sec; 72°C, 40 sec) x40] and the dissociation curve [95°C, 15 sec; 60°C, 1 min; 95°C, 15 sec]. Fungal burden was represented by the ratio of fungal-plant DNA. DON was extracted in 5 ml H_2_O from 0.3 g ground wheat heads for 30 mins 180 rpm at 30°C. The soluble fraction was diluted 1:6 in 50 mM Tris pH8, and subsequently 1:10 in H_2_O. DON was quantified by ELISA (Beacon Analytical Systems) per the manufactures instructions. The fungal burden and DON contamination for each treatment was calculated from two technical replicates and the analysis of 10–12 biological replicates.

### Yeast split ubiquitin

The split-ubiquitin system (Dualsystems Biotech) was used to investigate interaction between four *F*. *graminearum* GPCRs (FGRRES_02655, FGRRES_07270, FGRRES_07792 and FGRRES_16221) and Gα-proteins (Gpa1, Gpa2, and Gpa3). Full length cDNAs of the GPCRs were cloned into the pTMBV4 vector (the C-terminal half of ubiquitin plus the LexA::VP16 reporter [Cub] fused to the C-terminus of each receptor) and the *GPA1*, *GPA2* and *GPA3* cDNAs were cloned into pDL2XN (the N-terminal half of ubiquitin [NubG] was fused to the C-terminus of each G-protein). The non-GPCR interacting Alg5 membrane protein was fused with NubG. The *S*. *cerevisiae* strain NMY32 was transformed using the LiAc method. Briefly, an overnight culture of NMY32 was diluted to OD_600_ 0.3, in a final volume of 50 ml YPD broth, and incubated at 30°C to an OD_600_ of 0.6–1.0. Cells were harvested by centrifugation, washed in water, and resuspended in 100 mM LiAc in Tris-EDTA (10 mM Tris, 1 mM EDTA pH 7.4). 0.2 μg plasmid DNA was then incubated for 30 mins at 30°C with 100 μl competent cells, 10 μl freshly denatured ssDNA (10mg/ml), and 600 μl PEG-LiAc solution [100 μl 1M LiAc (pH 7.4), 100 μl H2O, 800 μl 50% (w/v) polyethylene glycol 3350 per ml]. 70ul DMSO was added and the transformation mixture was heat shocked for 15 mins at 42°C, then kept on ice for two mins. The yeast cells were washed, then resuspended, with water and plated on selective media (SD-leucine for Cub containing strains, and SD-tryptophan for NubG containing strains). Following confirmation of protein expression by Western blotting, GPCR-containing strains were co-transformed with each of the three Gα proteins by the same method. Interaction was determined by the growth of yeast transformants on a medium lacking histidine and/or adenine.

### Bioinformatics

The genomic localisation of the putative GPCRs was displayed on the four *F*. *graminearum* chromosomes [49] and aligned to regions associated with high frequency recombination [28] using Omnimap [50]. Orthologues of FGRRES_07792 and FGRRES_16221 were identified in 436 fungal genomes, representing the 12 of the 13 different taxonomic classes or subphyla within Dikarya, using the genomic resource PhytoPath (www.PhytoPathdb.org) [51]. No genomes for Lecanoromycetes were represented. Therefore, BlastP analyses using the mature FGRRES_07792 and FGRRES_16221 protein sequences were performed, at the expected (e-value) cut-off thresholds of 1x10^-50^ and 1x10^-100^, on the predicted proteomes of four Lecanoromycetes presented on the JGI Mycocosm portal [52]. The mature protein sequence, and the CFEM domains, of the eight class X CFEM-containing *F*. *graminearum* GPCRs and PTH11 from *M*. *oryzae* were aligned using ClustalW and a phylogenetic neighbour-joining PHYML tree was constructed.

### Bioimaging

The macroscopic appearance of representative diseased plants at 3–15 dpi was photographed on a Nikon D40X under natural light, while the excised inoculated spikelets and the rachis internodes below were imaged on a Lecia M205 stereomicroscope under bright field light. SEM was used to assess the establishment of floral colonisation. The wheat palea of the inoculated spikelet was excised at 2 dpi and mounted onto a cryo stub using a 50:50 mixture of OCT compound (Sakura FineTek) and colloidal graphite (TAAB) and plunge frozen in liquid nitrogen. Samples were transferred under vacuum to the GATAN ALTO 2100 cryo chamber stage maintained at −180°C. Paleae were etched for 1 min at −95°C prior to gold coating, and transferred to the cold stage of JEOL LV6360 SEM maintained at −150°C for histological examination of the progression of infection. Rachis internodes were fixed for 24 h with 4% paraformaldehyde, 2.5% glutaraldehyde in 0.05 M Sorensen’s phosphate buffer (NaH_2_PO_4_:Na_2_HPO_4_, pH7.2), then washed 3× with 0.05 M Sorensen’s buffer. Fixed rachis internodes were dehydrated in a graded ethanol series, embedded in medium grade LR white resin (TAAB) and polymerized at 60°C for 16h. Semi-thin 1 μm sections were cut on an ultramicrotome (Reichert-Jung, Ultracut) and collected on glass slides. After staining with aqueous 0.1% toluidine blue O (TBO) in 1% sodium tetraborate pH9, sections were mounted in DPX (Sigma Aldrich), then examined and imaged using a Zeiss Axiophot light microscope.

### RNA sequencing

Fungi were grown in yeast extract peptone dextrose (YPD; Sigma-Aldrich) broth for 3 days at 25°C and 180 rpm. Mycelia was collected by vacuum filtration, washed with sterile water and frozen in liquid nitrogen. Wheat spikelets and the sequential rachis segments below the inoculated spikelet were dissected and frozen in liquid nitrogen at 3 and 7 dpi. Ten plants were combined for each biological sample, and experiments were performed in triplicate. Total RNA was extracted from frozen mycelia and wheat tissues with Quick-RNA MiniPrep™ kits (Zymoresearch). RNA integrity was confirmed using the Bioanalyzer Nano kit (Agilent technologies) and the Agilent Bioanalyzer 2100. RNA sequencing libraries were polyA-based mRNA enriched and sequenced (non-strand specific paired-end reads) on the Illumina HiSeq4000 platform by BGI Tech Solutions (Hong Kong, China). Low quality reads were filtered using SOAPnuke [53]. To calculate expression values, clean reads were mapped to reference *F*. *graminearum* [49] and wheat (http://plants.ensembl.org/Triticum_aestivum/Info/Index/) genomes using HISAT2 [54]. StringTie [55] was used to reconstruct transcripts and novel transcripts identified using Cuffcompare [56]. Reference and novel transcripts were combined and mapped onto the reference genomes using Bowtie2 [57]. Gene expression values (FPMK) were calculated using RSEM [58]. Pearson correlation identified outliers were removed from the analysis. DEGs (FDR <0.05, ±1log2 fold change in expression) were identified using read counts in EdgeR [59] and overrepresented GO terms (FDR <0.05 and <0.01) were identified using Blast2GO [60].

### Secreted lipase quantification

Fungal strains (10 μl 1x10^6^ conidia) were grown in liquid SNA media with 2% wheat germ oil (Invitrogen) as the sole carbon source for 3 days at 25°C and 180 rpm. Media was supplemented with 25 μl Rhodamine B solution (0.001% w/v). Fungal cultures were photographed on a Nikon D40X under natural light. Lipase secretion was detected by measuring the fluorescence (Ex 540 nm, Em 600 nm) of the supernatant on a SpectraMax i3 (Molecular Devices).

## Supporting information

S1 Fig The expression of the selected putative GPCRs in *Fusarium graminearum* during growth in axenic culture or during the progression of wheat head infection. **A)** The absolute expression profiles reveal FGRRES_07792 to be the most highly expressed putative GPCR encoding gene during wheat infection, in particular during symptomatic wheat infection. **B)** The fold change in GPCR encoding gene expression during wheat infection when compared to axenic culture in nutrient-rich complete media, reveals FGRRES_16221 and FGRRES_03151 to have the highest level of transcriptional induction during the establishment of symptomless wheat infection. Presented are selected fungal classes I-V and X (with or without a cysteine-rich extracellular CFEM domain) GPCRs. Axenic culture represents CM = complete media, MM-C = carbon starvation, MM-N = nitrogen starvation. Wheat infection is represented by the distinct infection phases at 7 days post infection, namely symptomless rachis infection, intermediate rachis infection, symptomatic rachis and symptomatic spikelet infection. * denotes three putative GPCR encoding genes with either the highest absolute expression during plant infection or the highest fold change in gene induction during the establishment of symptomless infection.
(TIF)

S2 Fig The identification of *Fusarium graminearum* GPCRs that contribute to virulence. Initial schematic depicts the split marker-mediated strategy for generating fungal mutants lacking individual GPCR encoding genes. The hygromycin gene (HYG) was inserted in the opposite orientation to the GPCR encoding gene. The impact of single GPCR encoding gene deletions on wheat infection was assessed by determining the number of diseased spikelets below the point of inoculation at 15 days post infection (dpi). Histograms show the absence of individual classical class I-V receptors had no significant impact on virulence, while the absence of several non-classical class X receptors (with or without a CFEM domain) resulted in an attenuation of virulence. Later schematics and images confirm the generation of two independent *F*. *graminearum* mutants for the deletion, truncation or complementation strains, and the subsequent assessment of the impact of these mutations on virulence, compared to the parental PH-1 strain and the mock non-infected controls. Fusarium Head Blight symptoms are presented at 15 dpi.
(PDF)

S3 Fig The *Fusarium graminearum* mutants lacking FGRRES_16221 or FGRRES_07792 show reduced disease causing abilities on a non-host plant species. **A)** The appearance of reduced disease symptoms on the model non-host *Arabidopsis thaliana* floral system, and **B)** a boxplot showing the moderate reduction in the Fusarium-Arabidopsis disease index combining flower and silique infection at 7 days post infection (dpi).
(TIF)

S4 Fig The *Fusarium graminearum* mutants lacking FGRRES_16221 or FGRRES_07792 show no defects during *in vitro* growth or sexual development. **A)** Fungal growth on nutrient-rich (PDA) and nutrient-poor (SNA) media, after 5 and 3 days incubation, respectively. **B)** Fungal growth on a range of simple and complex plant-derived carbon sources. Images represent 3 days radial growth on SNA-C media supplemented with 1% of the respective carbon source. CMC = carboxymethyl cellulose. **C)** Sexual perithecial development after 14 days incubation on carrot agar. Presented are two independent *F*. *graminearum* mutants lacking either FGRRES_16221 or FGRRES_07792, compared to the parental PH-1 strain.
(TIF)

S5 Fig Yeast split ubiquitin assay shows *Fusarium graminearum* sex pheromone receptors interact with g-proteins. **A)** Schematic depicts the yeast split ubiquitin approach for identification of receptor-G-protein interactions at the cell membrane. **B)** Assay demonstrates classical class I and II pheromone receptors physically interact with multiple Gα-proteins at the cell membrane. Yeast serial dilutions (1:1, 1:10, 1:100, 1:1000) were grown on non-selective SD media lacking tryptophan (T) and leucine (L), plus selective media also lacking histidine (H) and adenine (A). The Alg5 membrane protein is a non-GPCR interacting control.
(TIF)

S6 Fig Secondary metabolite biosynthetic gene clusters expression. Accumulative FPKM expression values for *Fusarium graminearum* secondary metabolite biosynthetic gene clusters during axenic culture and wheat infection by the PH-1 and Δ16221_3 strains. Presented are clusters which produce characterised metabolites and clusters which are associated with virulence. Legend: YPD = axenic culture in YPD, SP_3d = spikelet 3 dpi, SP_7d = spikelet 7 dpi, R1-8_7d = pooled pairs of rachis internodes below inoculated spikelet at 7 dpi.
(TIF)

S7 Fig The *Fusarium graminearum* mutants lacking FGRRES_16221 are defective in iron homeostasis. **A)** Four days radial growth on SNA with and without iron chelator 2–2’-dipyridyl (2DP) shows the Δ16221 mutants are increasingly sensitive to iron stress. **B)** Accumulative FPKM expression values for siderophore biosynthetic gene clusters during axenic culture and wheat infection by the PH-1 and Δ16221_3 strains. This shows the differential modulation of iron scavenging siderophore during wheat infection. Legend: YPD = axenic culture in YPD, SP_3d = spikelet 3 dpi, SP_7d = spikelet 7 dpi, R1-8_7d = pooled pairs of rachis internodes below inoculated spikelet at 7 dpi.
(TIF)

S8 Fig The *Fusarium graminearum* mutants lacking FGRRES_16221 show reduced lipase secretion. The respective fungal strains were grown in 50 ml SNA media with 2% wheat germ oil as the sole carbon source, plus 0.0001% rhodamine B, for 4 days at 25°C 180, rpm. **A)** Representative cultures of the three fungal strains. **B)** In the absence of FGRRES_16221 the mean fluorescence of the culture is decreased, showing a reduction in secreted lipase activity. ** = *p*<0.01.
(TIF)

S9 Fig The sampling wheat rachis tissues for the RNA-sequencing investigation. The sequential rachis internodes below the inoculated spikelets at 7 day post infection. This includes wheat rachis internodes from healthy non-infected, and *Fusarium graminearum* infected (either the parental PH-1 strain or Δ16221_3 mutant) plants. SP = inoculated spikelet. R1-6 = rachis internodes below inoculated spikelet. Pairs of rachis segments, which were phenotypically similar in the parental PH-1 infection, were combined, representing the transcriptionally distinct fully symptomatic (R1-2), intermediate (R3-4) and symptomless (R5-6) infection phases [2,4].
(TIF)

S1 Table The primers used in this study. (XLSX)

S1 File Pairwise analyses of transcriptional differences in the *Fusarium graminearum* PH-1 and δ16221_3 strains during the establishment and progression of wheat infection. Lists of *F*. *graminearum* genes, functional annotation, mean FPKM gene expression values, identified differentially expressed genes (DEGs; FDR <0.05, ±1log2 fold change in expression) and the overrepresented gene ontologies (GO terms; FDR <0.05 and <0.01).
(XLSX)

S2 File Expression profiles for components of GPCR signalling. The mean FPKM expression profiles of components of GPCR, G-protein and MAPK signalling pathways in the *Fusarium graminearum* PH-1 and Δ16221_3 strains during in vitro culture and the progression of wheat infection.
(XLSX)

S3 File Pairwise analyses of transcriptional differences in wheat during the establishment and progression of infection by the *Fusarium graminearum* PH-1 and δ16221_3 strains. Lists of wheat genes, functional annotation, mean FPKM gene expression values, identified differentially expressed genes (DEGs; FDR <0.05, ±1log2 fold change in expression) and the overrepresented gene ontologies (GO terms; FDR <0.01).
(XLSX)
