# Supplementary figures and images for: Non-canonical fungal G-protein coupled receptors promote Fusarium head blight on wheat
Source: PLoS Pathog. 2019 Apr 1;15(4):e1007666. doi: 10.1371/journal.ppat.1007666 (PMC6459559; doi:10.1371/journal.ppat.1007666)

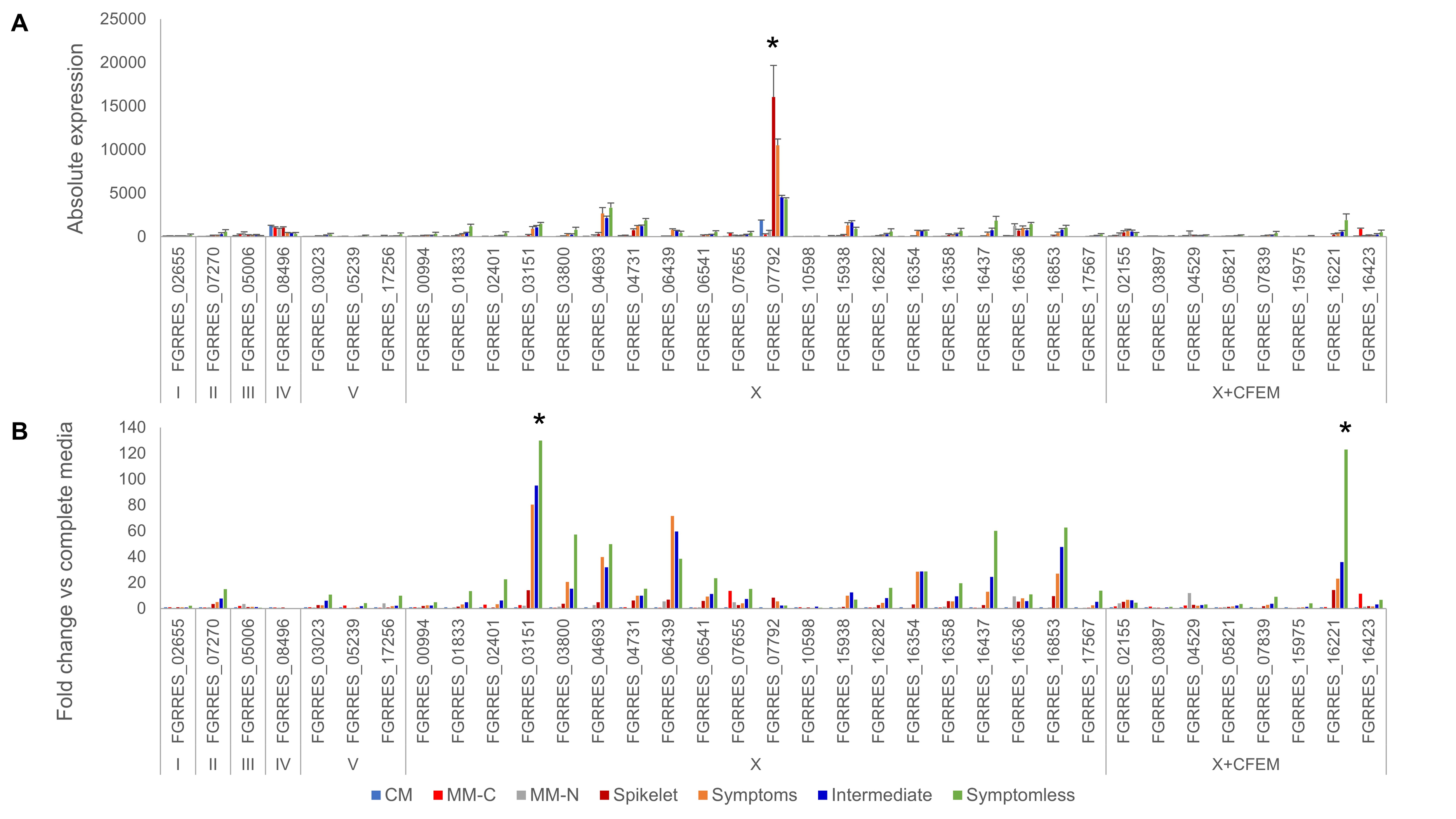

Supplement: S1 Fig — A) The absolute expression profiles reveal FGRRES_07792 to be the most highly expressed putative GPCR encoding gene during wheat infection, in particular during symptomatic wheat infection. B) The fold change in GPCR encoding gene expression during wheat infection when compared to axenic culture in nutrient-rich complete media, reveals FGRRES_16221 and FGRRES_03151 to have the highest level of transcriptional induction during the establishment of symptomless wheat infection. Presented are selected fungal classes I-V and X (with or without a cysteine-rich extracellular CFEM domain) GPCRs. Axenic culture represents CM = complete media, MM-C = carbon starvation, MM-N = nitrogen starvation. Wheat infection is represented by the distinct infection phases at 7 days post infection, namely symptomless rachis infection, intermediate rachis infection, symptomatic rachis and symptomatic spikelet infection. * denotes three putative GPCR encoding genes with either the highest absolute expression during plant infection or the highest fold change in gene induction during the establishment of symptomless infection. (TIF) [file ppat.1007666.s001.tif]

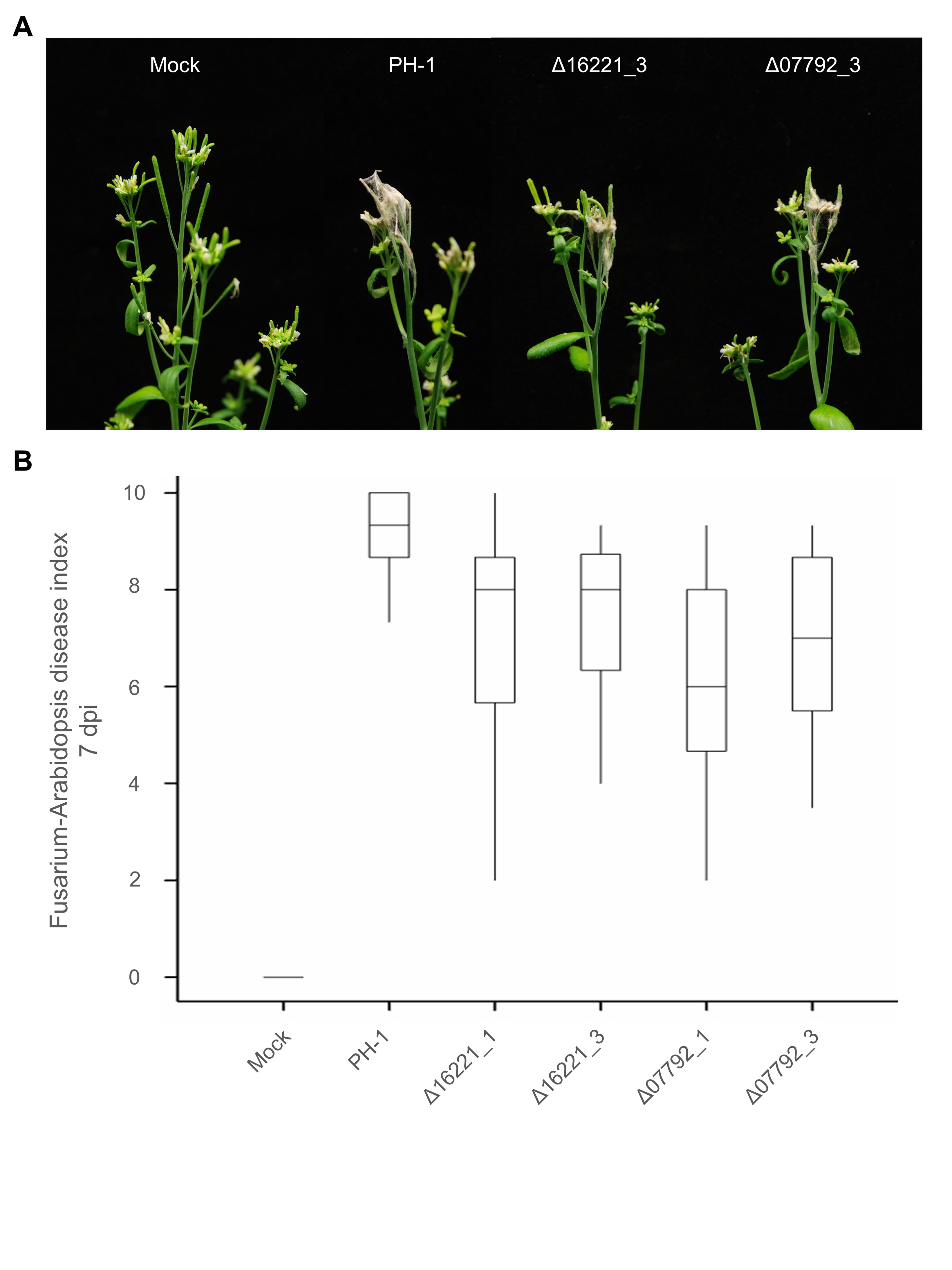

Supplement: S3 Fig — A) The appearance of reduced disease symptoms on the model non-host Arabidopsis thaliana floral system, and B) a boxplot showing the moderate reduction in the Fusarium-Arabidopsis disease index combining flower and silique infection at 7 days post infection (dpi). (TIF) [file ppat.1007666.s003.tif]

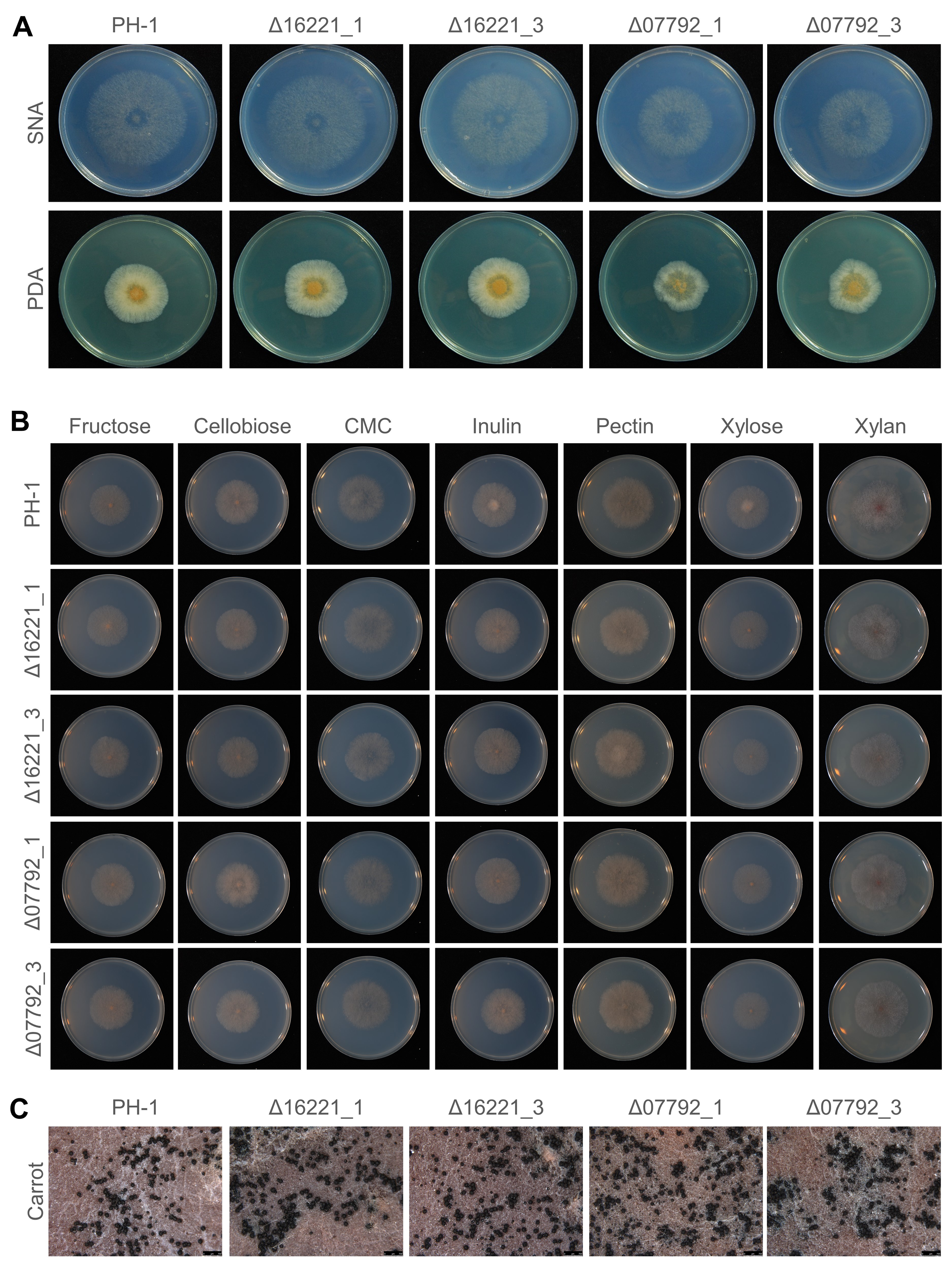

Supplement: S4 Fig — A) Fungal growth on nutrient-rich (PDA) and nutrient-poor (SNA) media, after 5 and 3 days incubation, respectively. B) Fungal growth on a range of simple and complex plant-derived carbon sources. Images represent 3 days radial growth on SNA-C media supplemented with 1% of the respective carbon source. CMC = carboxymethyl cellulose. C) Sexual perithecial development after 14 days incubation on carrot agar. Presented are two independent F. graminearum mutants lacking either FGRRES_16221 or FGRRES_07792, compared to the parental PH-1 strain. (TIF) [file ppat.1007666.s004.tif]

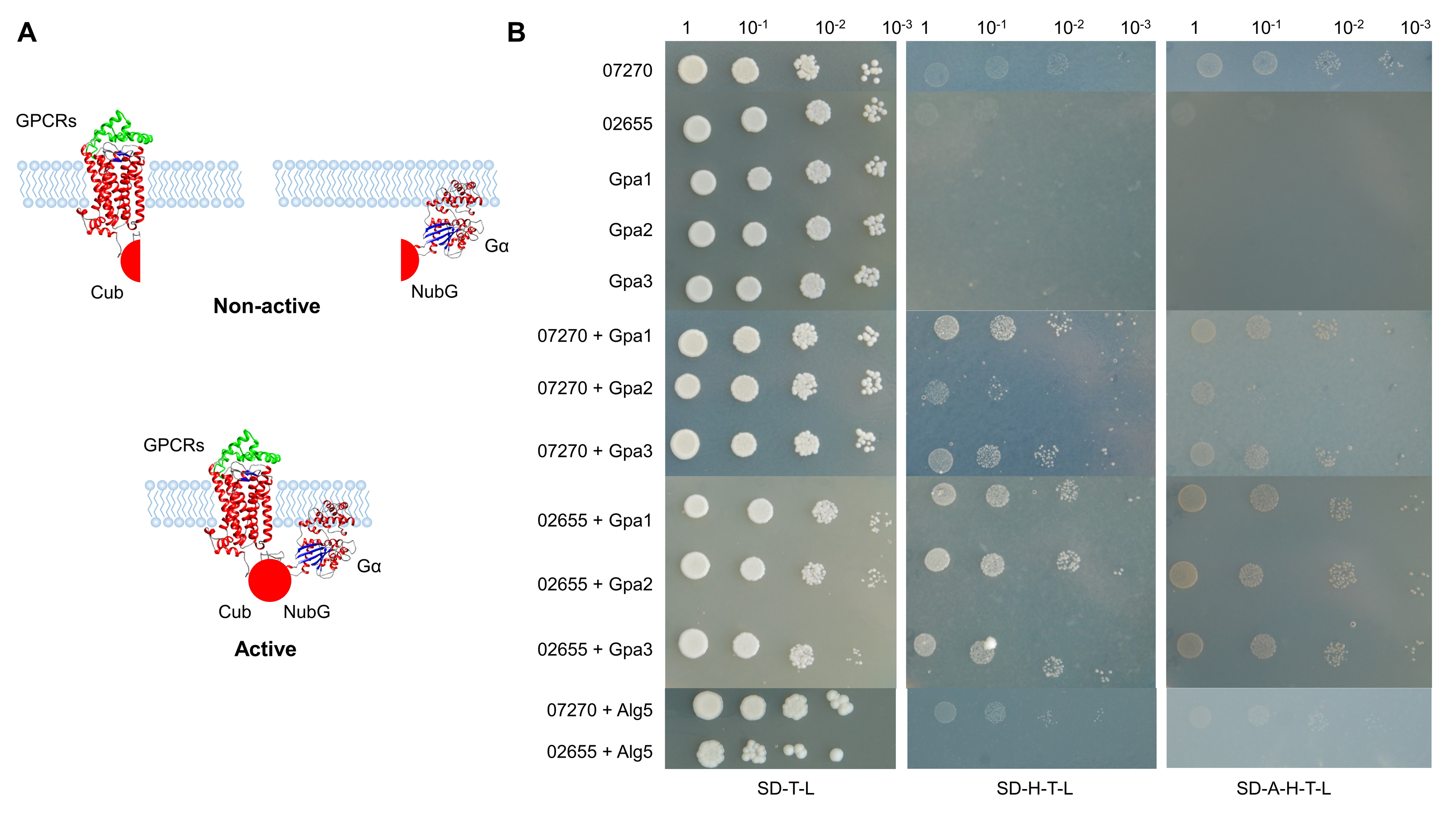

Supplement: S5 Fig — A) Schematic depicts the yeast split ubiquitin approach for identification of receptor-G-protein interactions at the cell membrane. B) Assay demonstrates classical class I and II pheromone receptors physically interact with multiple Gα-proteins at the cell membrane. Yeast serial dilutions (1:1, 1:10, 1:100, 1:1000) were grown on non-selective SD media lacking tryptophan (T) and leucine (L), plus selective media also lacking histidine (H) and adenine (A). The Alg5 membrane protein is a non-GPCR interacting control. (TIF) [file ppat.1007666.s005.tif]

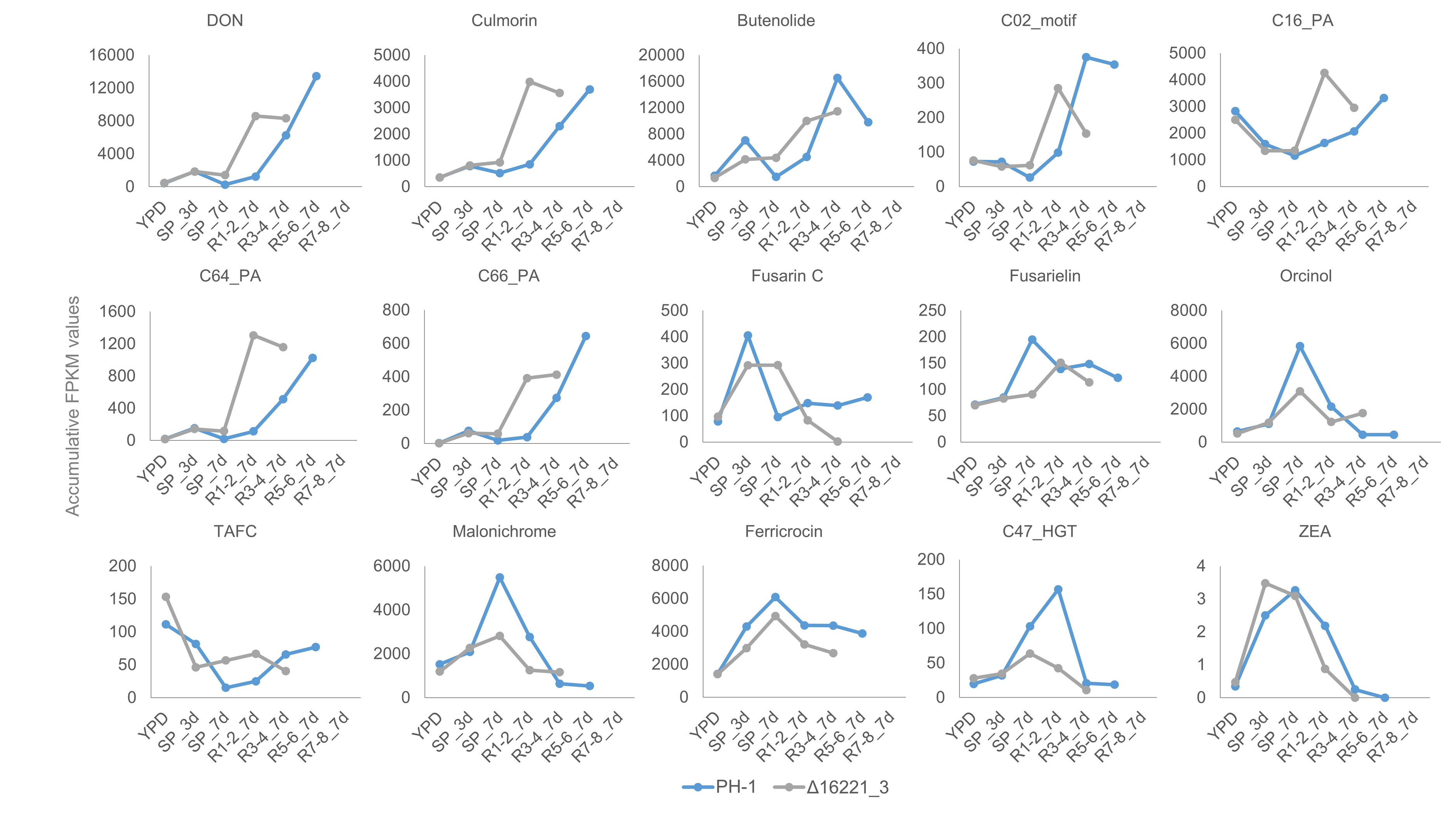

Supplement: S6 Fig — Accumulative FPKM expression values for Fusarium graminearum secondary metabolite biosynthetic gene clusters during axenic culture and wheat infection by the PH-1 and Δ16221_3 strains. Presented are clusters which produce characterised metabolites and clusters which are associated with virulence. Legend: YPD = axenic culture in YPD, SP_3d = spikelet 3 dpi, SP_7d = spikelet 7 dpi, R1-8_7d = pooled pairs of rachis internodes below inoculated spikelet at 7 dpi. (TIF) [file ppat.1007666.s006.tif]

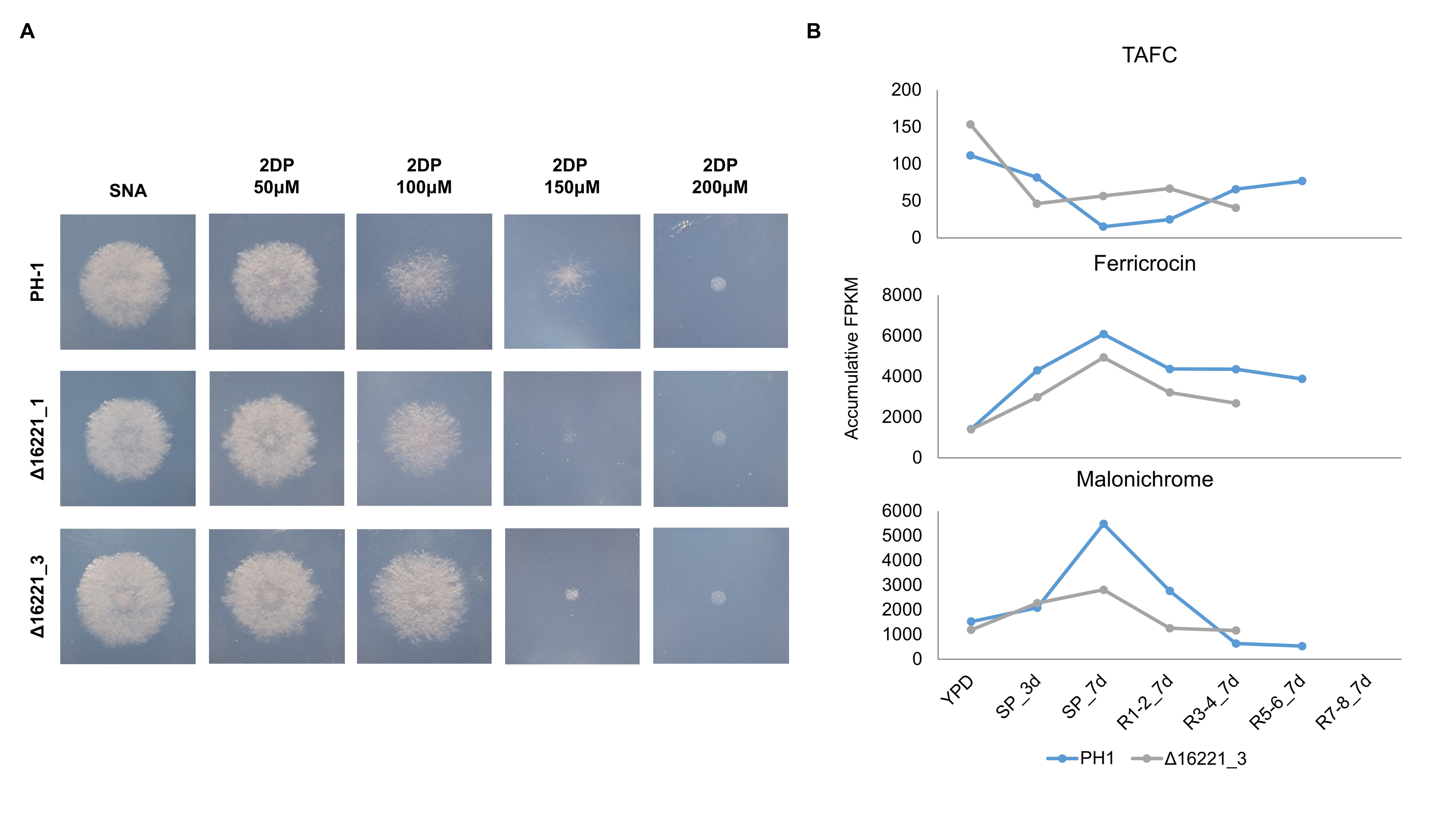

Supplement: S7 Fig — A) Four days radial growth on SNA with and without iron chelator 2–2’-dipyridyl (2DP) shows the Δ16221 mutants are increasingly sensitive to iron stress. B) Accumulative FPKM expression values for siderophore biosynthetic gene clusters during axenic culture and wheat infection by the PH-1 and Δ16221_3 strains. This shows the differential modulation of iron scavenging siderophore during wheat infection. Legend: YPD = axenic culture in YPD, SP_3d = spikelet 3 dpi, SP_7d = spikelet 7 dpi, R1-8_7d = pooled pairs of rachis internodes below inoculated spikelet at 7 dpi. (TIF) [file ppat.1007666.s007.tif]

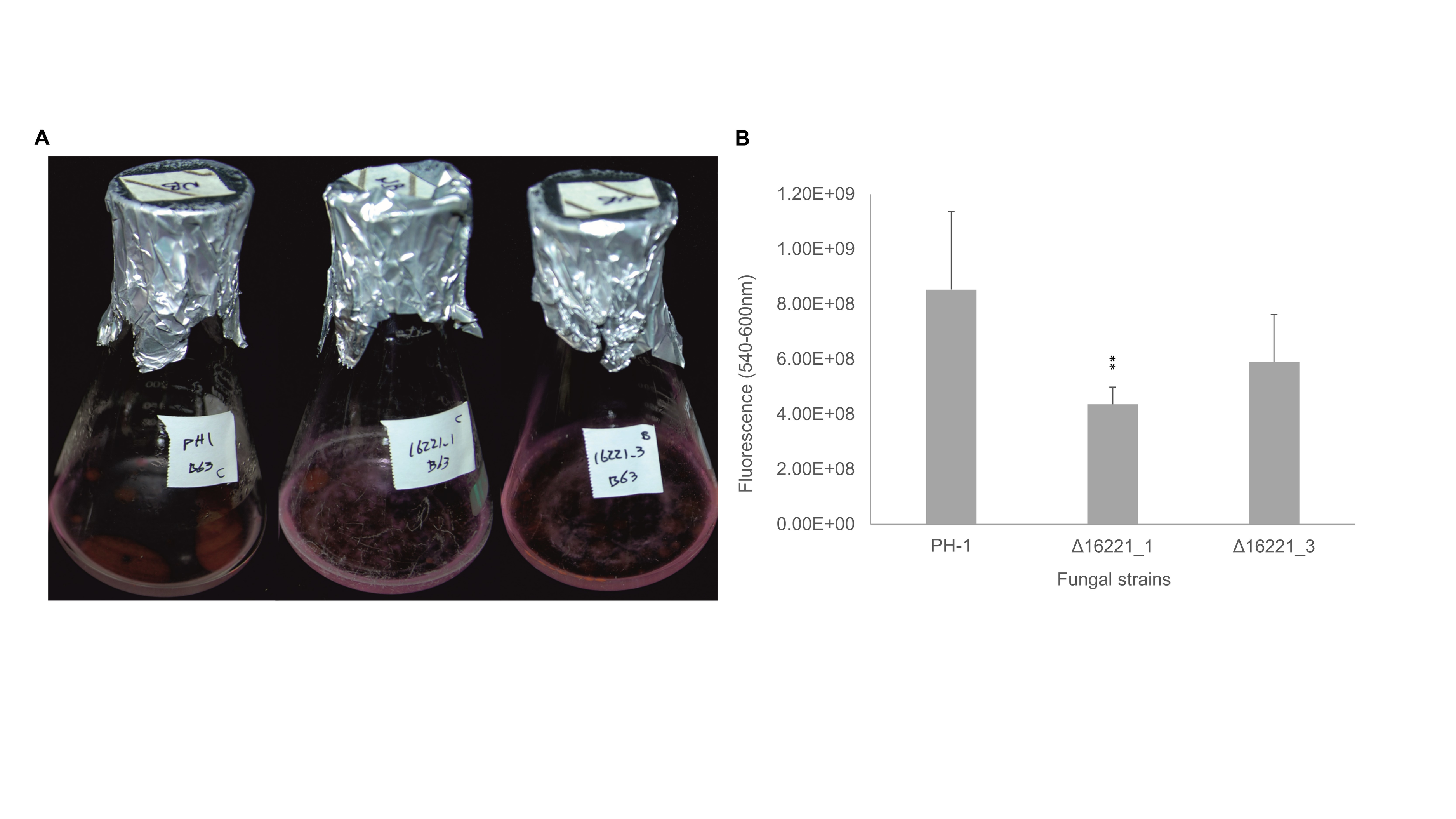

Supplement: S8 Fig — The respective fungal strains were grown in 50 ml SNA media with 2% wheat germ oil as the sole carbon source, plus 0.0001% rhodamine B, for 4 days at 25°C 180, rpm. A) Representative cultures of the three fungal strains. B) In the absence of FGRRES_16221 the mean fluorescence of the culture is decreased, showing a reduction in secreted lipase activity. ** = p<0.01. (TIF) [file ppat.1007666.s008.tif]

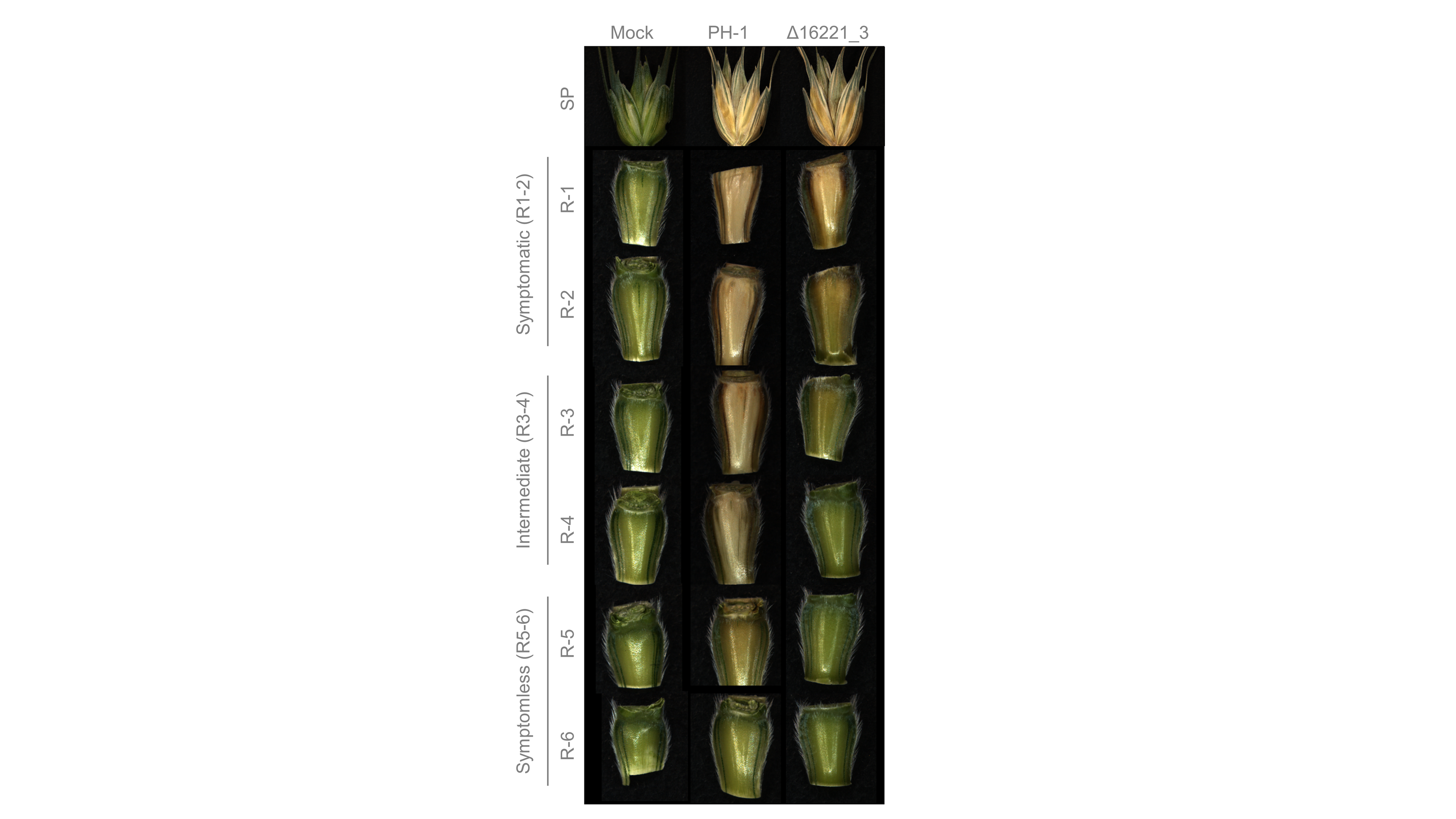

Supplement: S9 Fig — The sequential rachis internodes below the inoculated spikelets at 7 day post infection. This includes wheat rachis internodes from healthy non-infected, and Fusarium graminearum infected (either the parental PH-1 strain or Δ16221_3 mutant) plants. SP = inoculated spikelet. R1-6 = rachis internodes below inoculated spikelet. Pairs of rachis segments, which were phenotypically similar in the parental PH-1 infection, were combined, representing the transcriptionally distinct fully symptomatic (R1-2), intermediate (R3-4) and symptomless (R5-6) infection phases [2,4]. (TIF) [file ppat.1007666.s009.tif]
