# Supplementary material for: Non-canonical fungal G-protein coupled receptors promote Fusarium head blight on wheat
Source: PLoS Pathog. 2019 Apr 1;15(4):e1007666. doi: 10.1371/journal.ppat.1007666 (PMC6459559; doi:10.1371/journal.ppat.1007666)
Supplement: S2 Fig — Initial schematic depicts the split marker-mediated strategy for generating fungal mutants lacking individual GPCR encoding genes. The hygromycin gene (HYG) was inserted in the opposite orientation to the GPCR encoding gene. The impact of single GPCR encoding gene deletions on wheat infection was assessed by determining the number of diseased spikelets below the point of inoculation at 15 days post infection (dpi). Histograms show the absence of individual classical class I-V receptors had no significant impact on virulence, while the absence of several non-classical class X receptors (with or without a CFEM domain) resulted in an attenuation of virulence. Later schematics and images confirm the generation of two independent F. graminearum mutants for the deletion, truncation or complementation strains, and the subsequent assessment of the impact of these mutations on virulence, compared to the parental PH-1 strain and the mock non-infected controls. Fusarium Head Blight symptoms are presented at 15 dpi. (PDF) [file ppat.1007666.s002.pdf]

Split marker mediated gene replacement

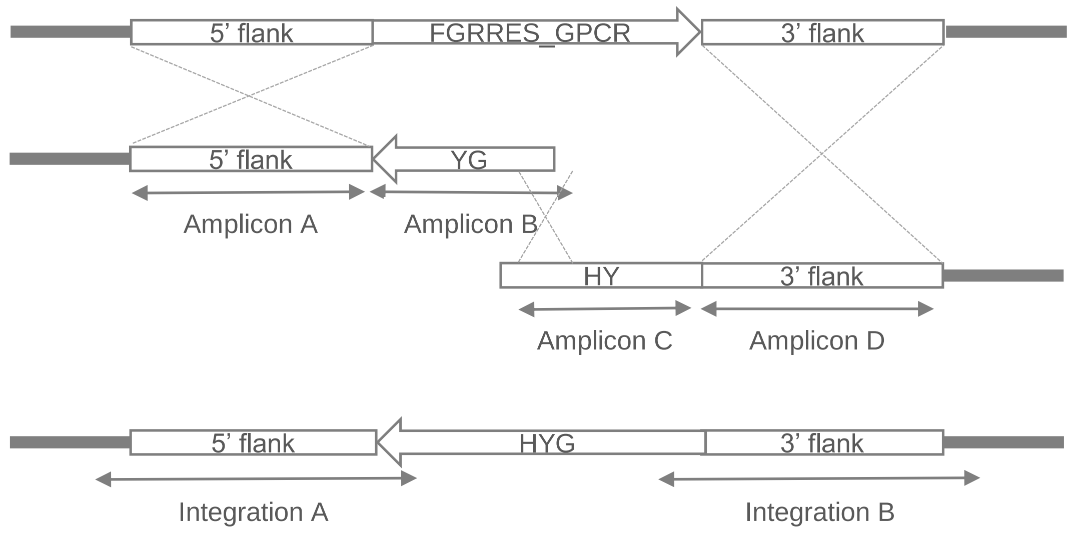

Impact of classical GPCR mutants on FHB symptoms

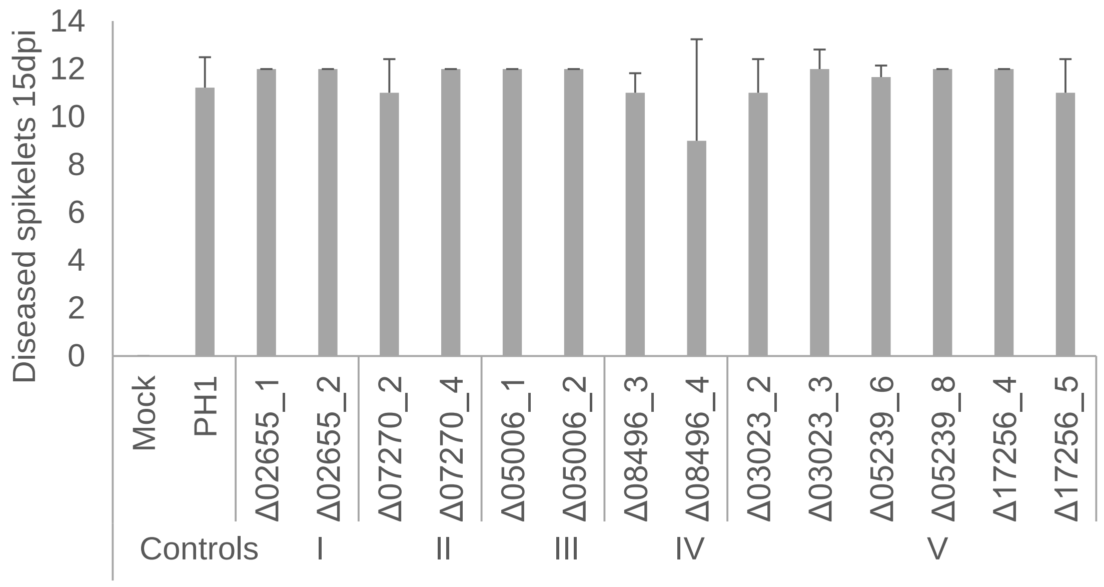

Impact of non-classical class X GPCR mutants on FHB symptoms

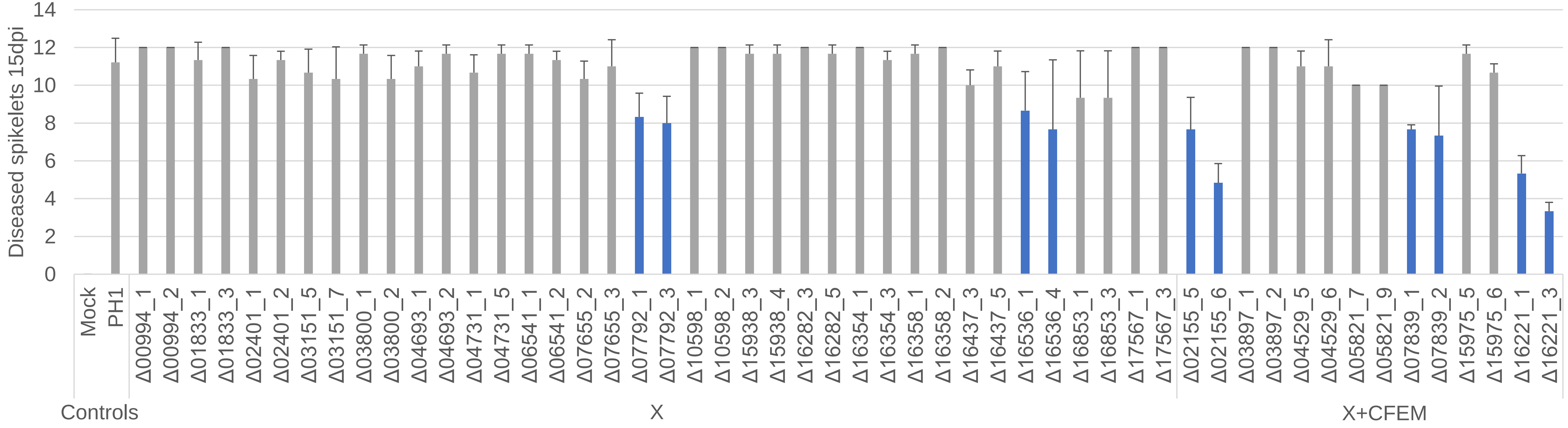

Class I: FGRRES_02655

Split marker mediated gene replacement

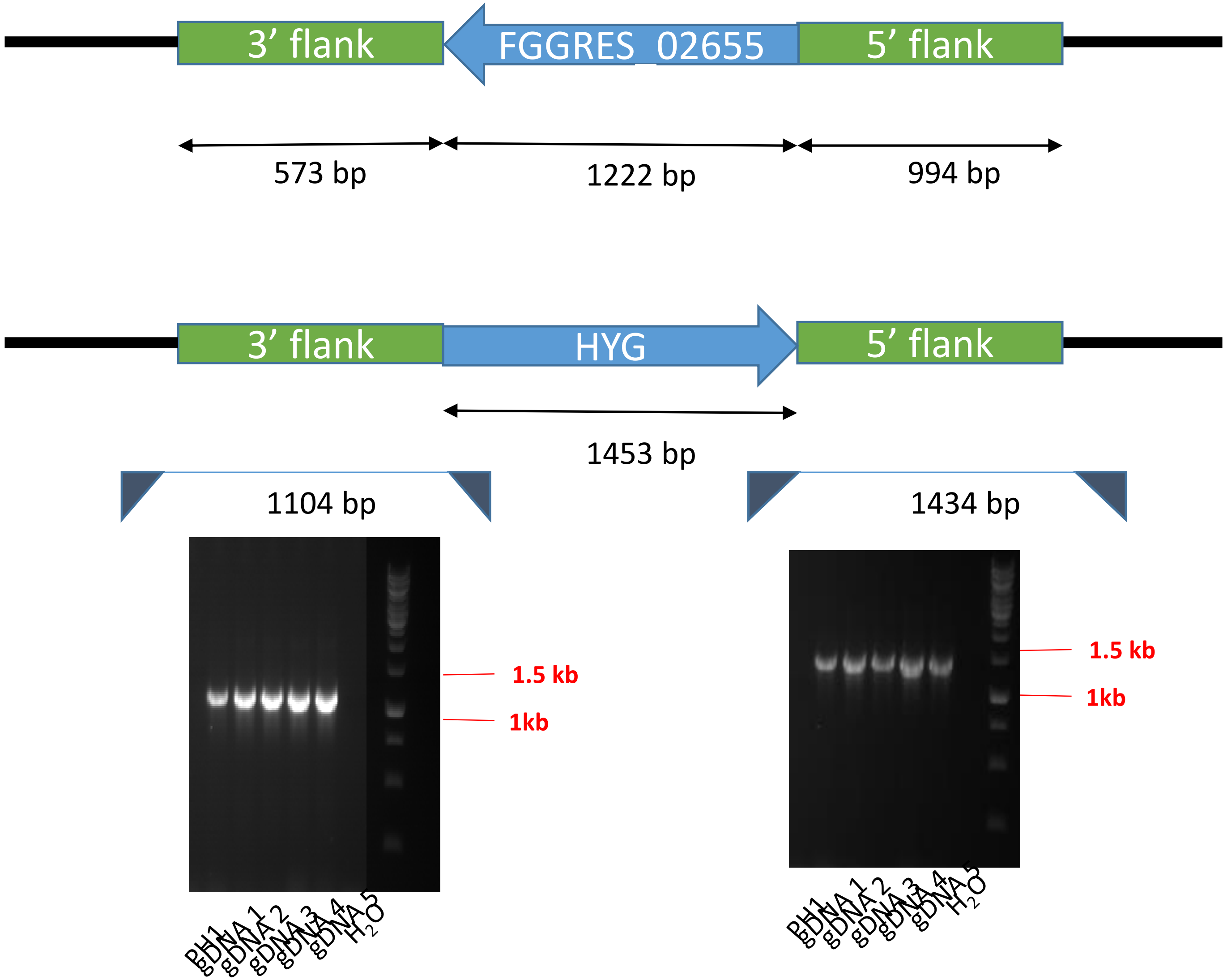

FHB symptoms on wheat 15 days post infection

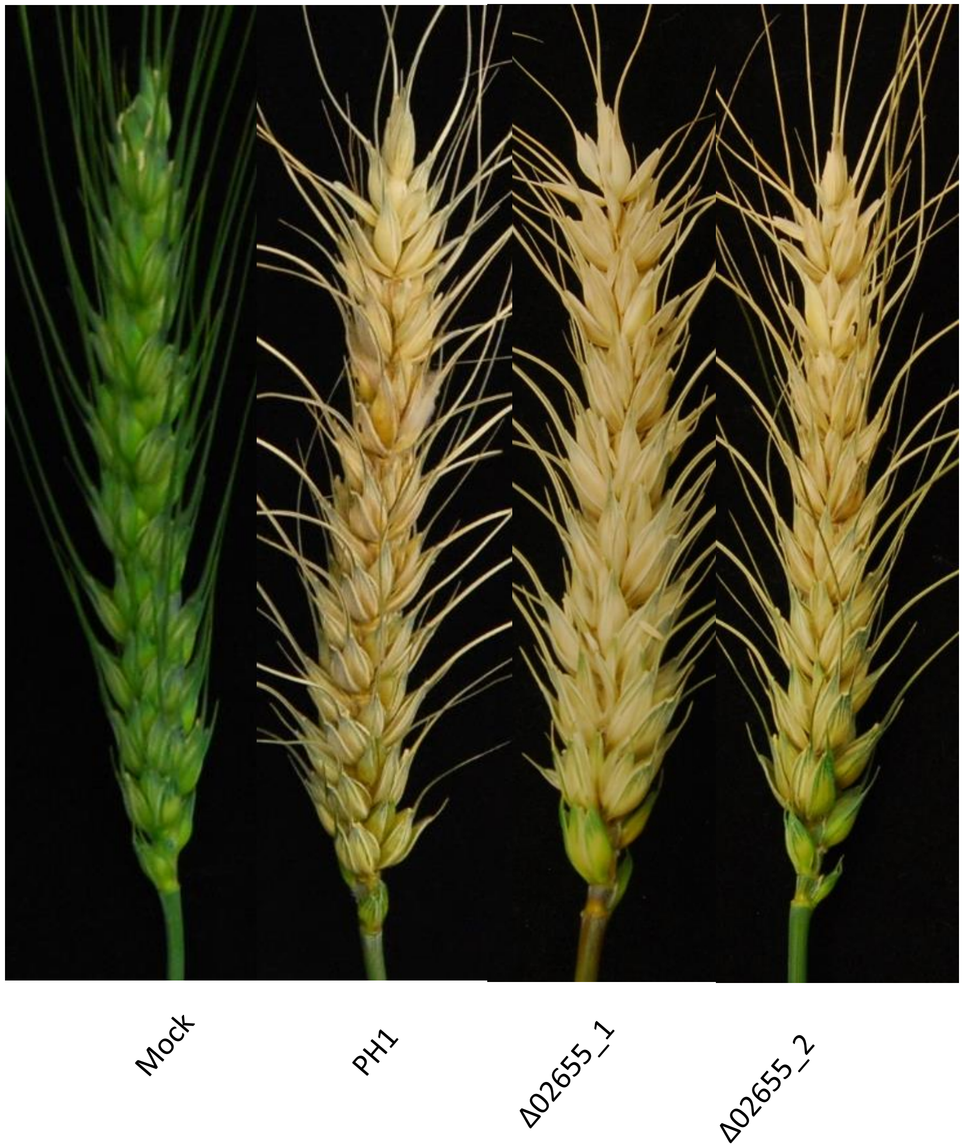

Class II: FGRRES_07270

Split marker mediated gene replacement

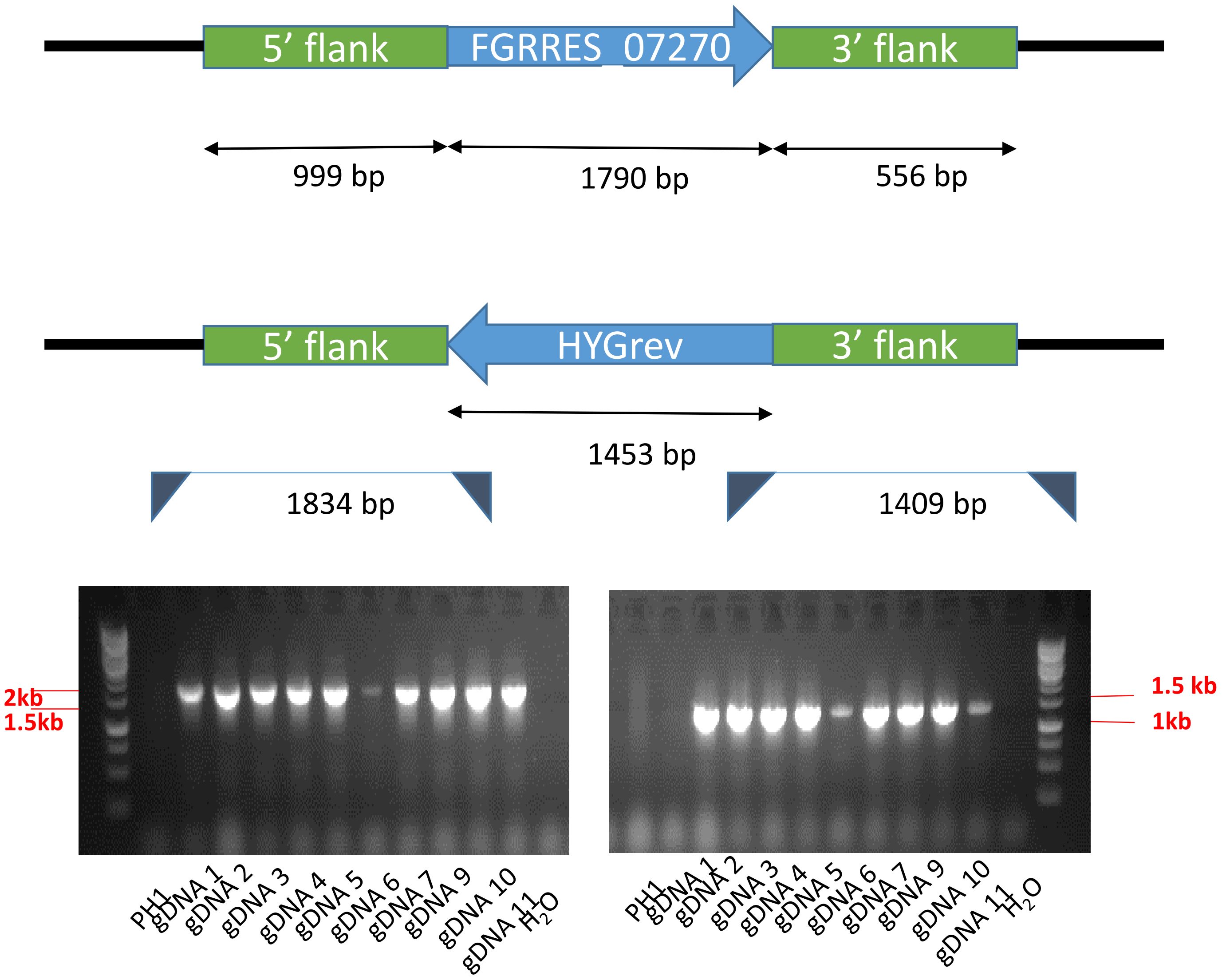

FHB symptoms on wheat 15 days post infection

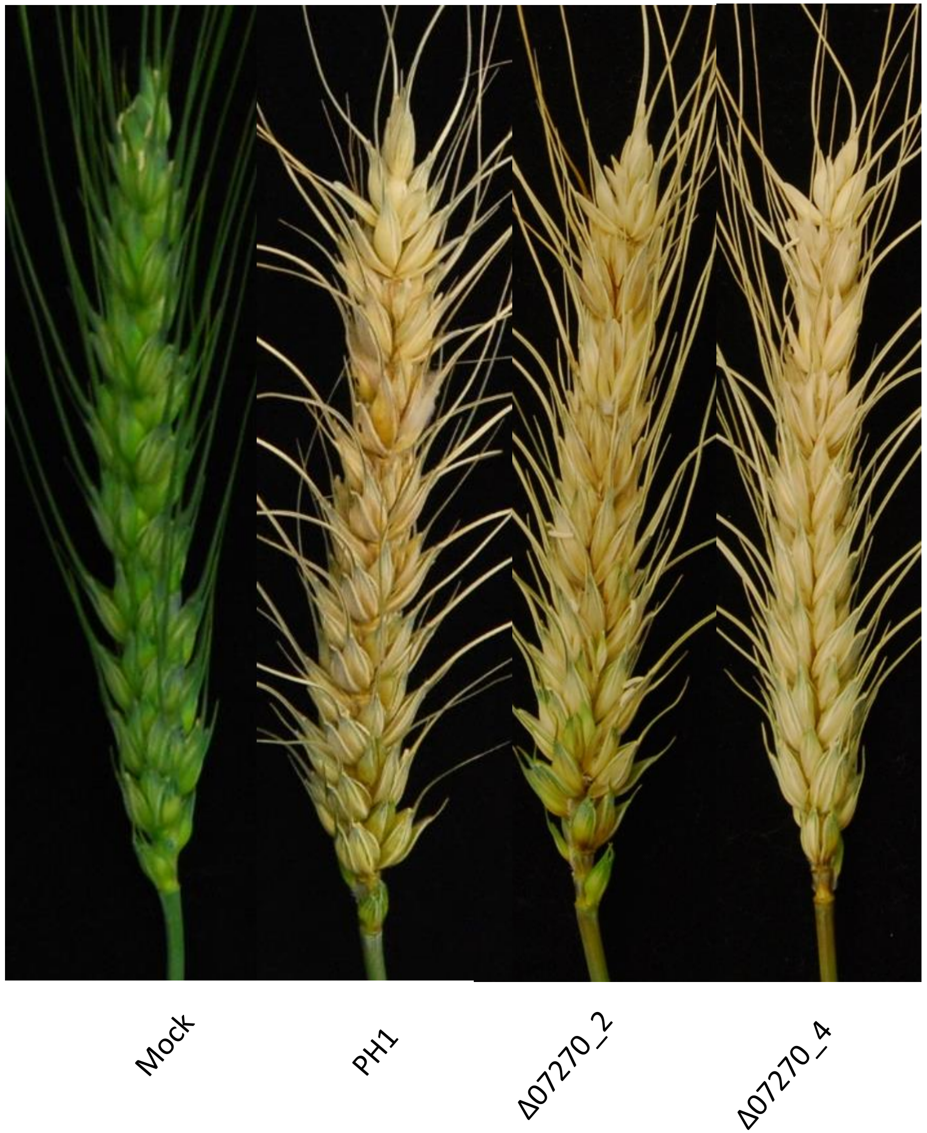

Class III: FGRRES_05006

Split marker mediated gene replacement

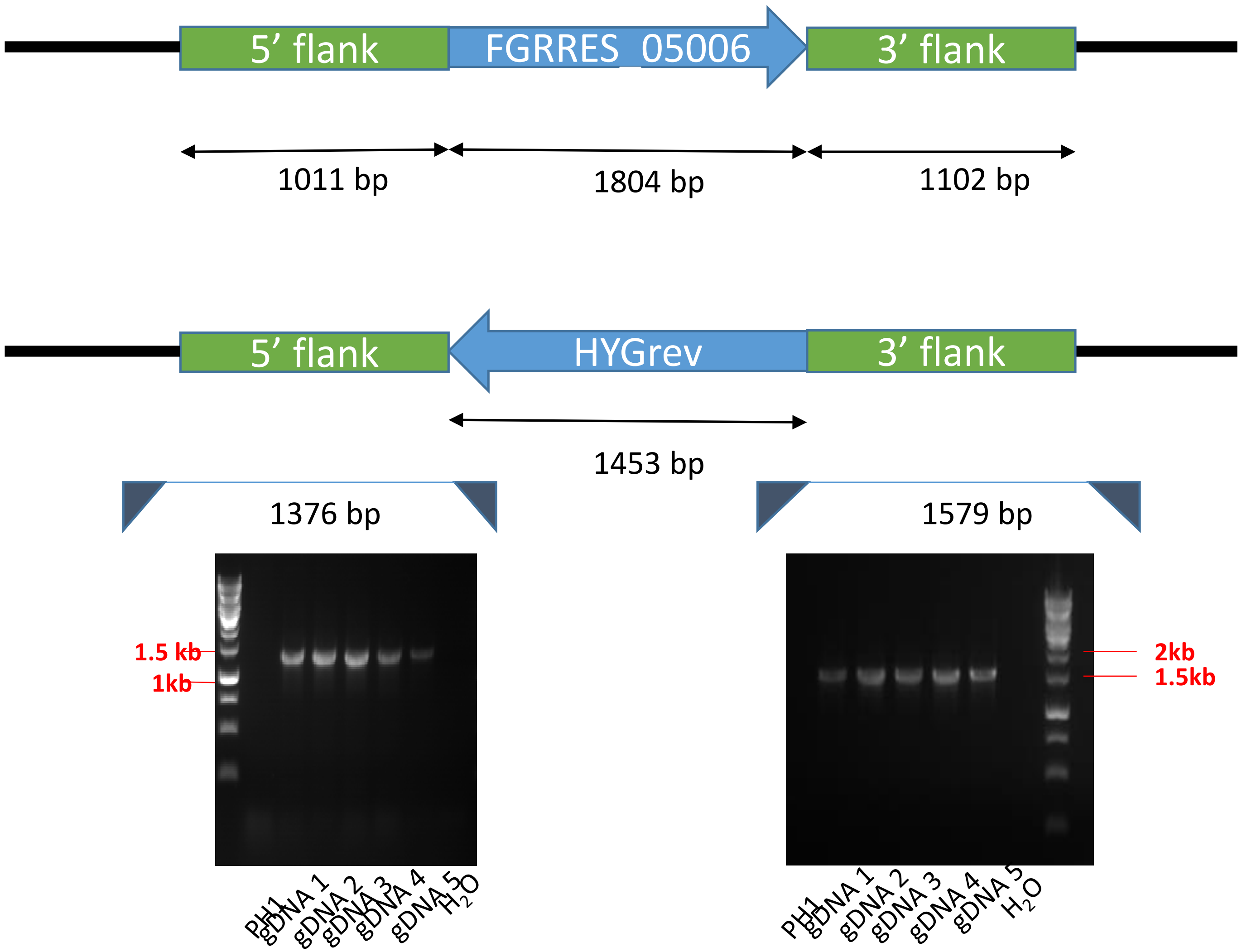

FHB symptoms on wheat 15 days post infection

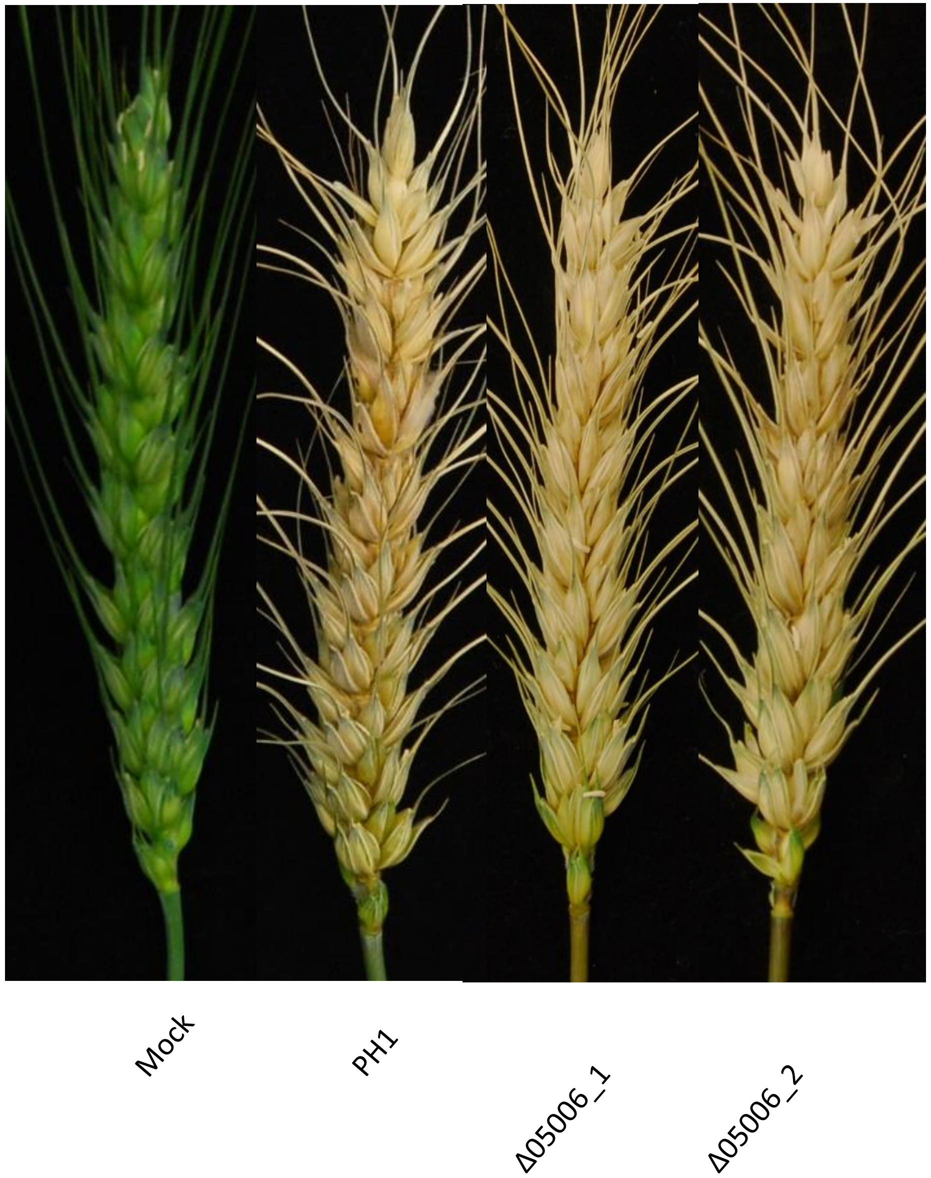

Class IV: FGRRES_08496

Split marker mediated gene replacement

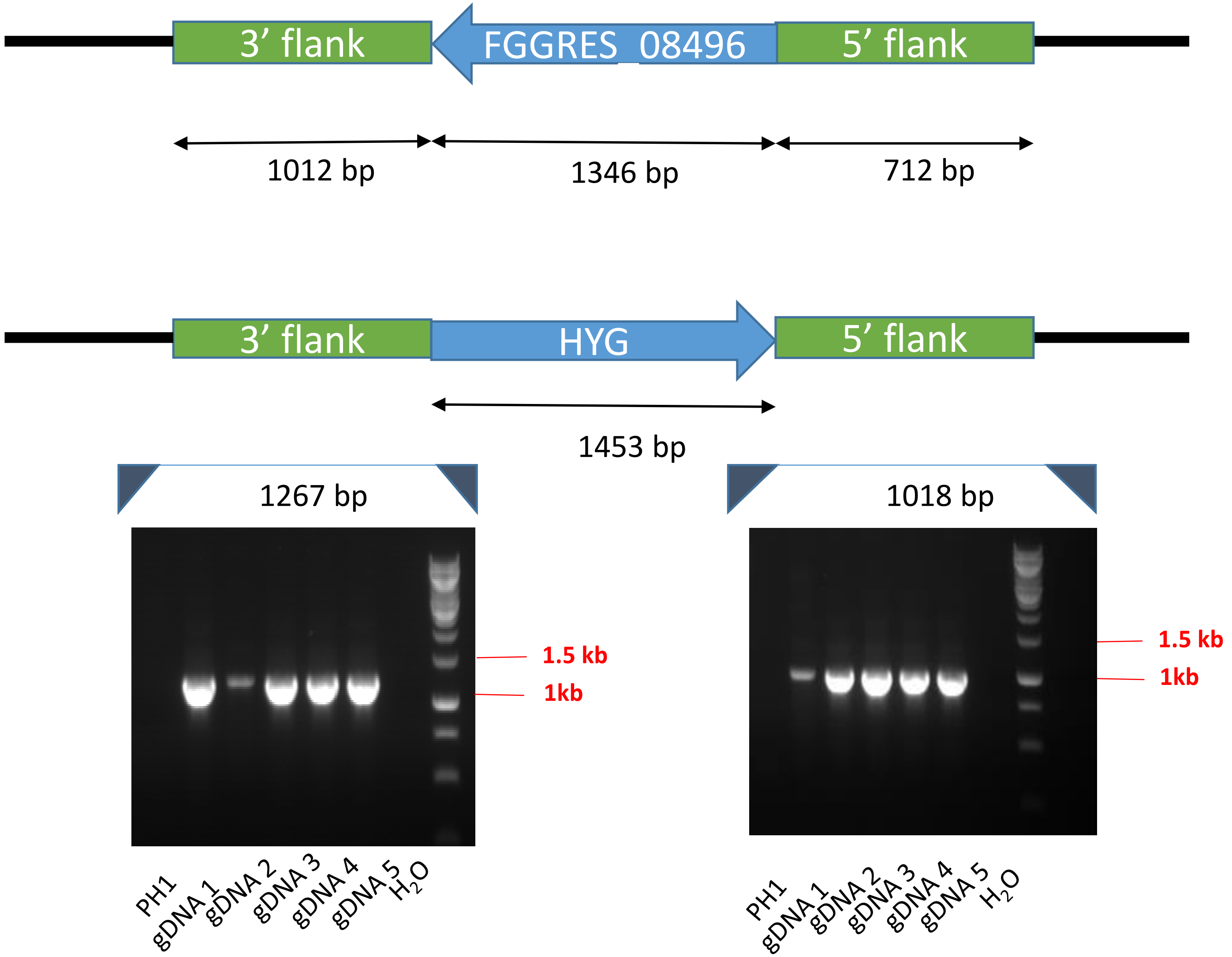

FHB symptoms on wheat 15 days post infection

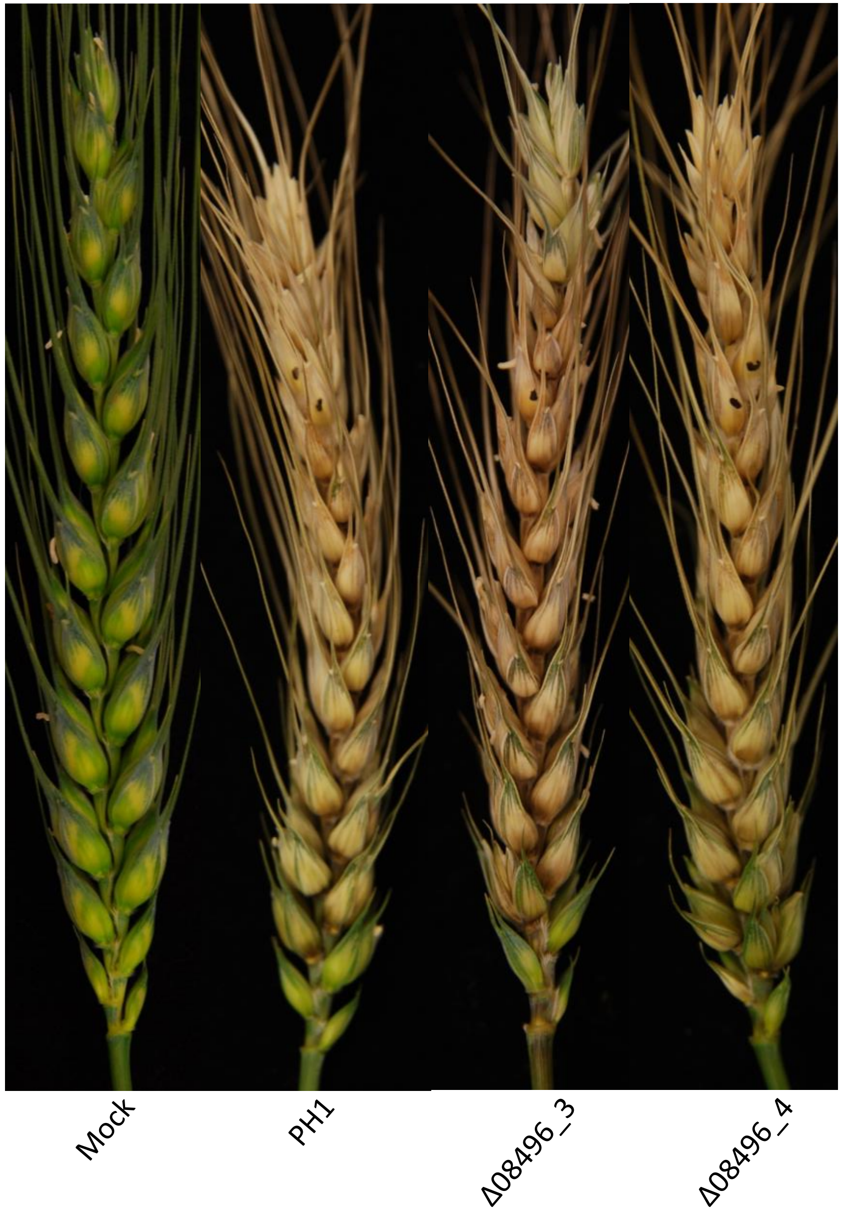

Class V: FGRRES_03023

Split marker mediated gene replacement

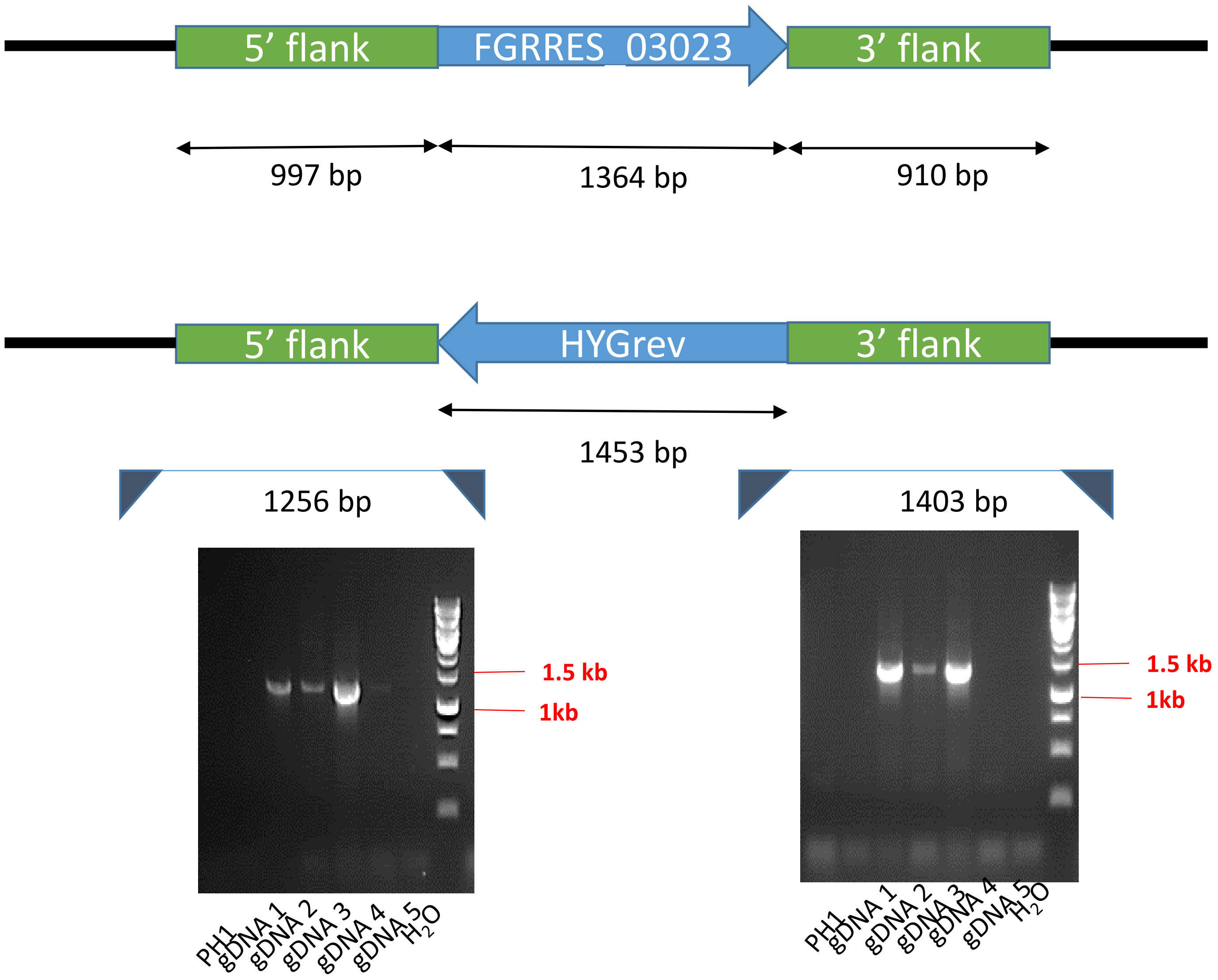

FHB symptoms on wheat 15 days post infection

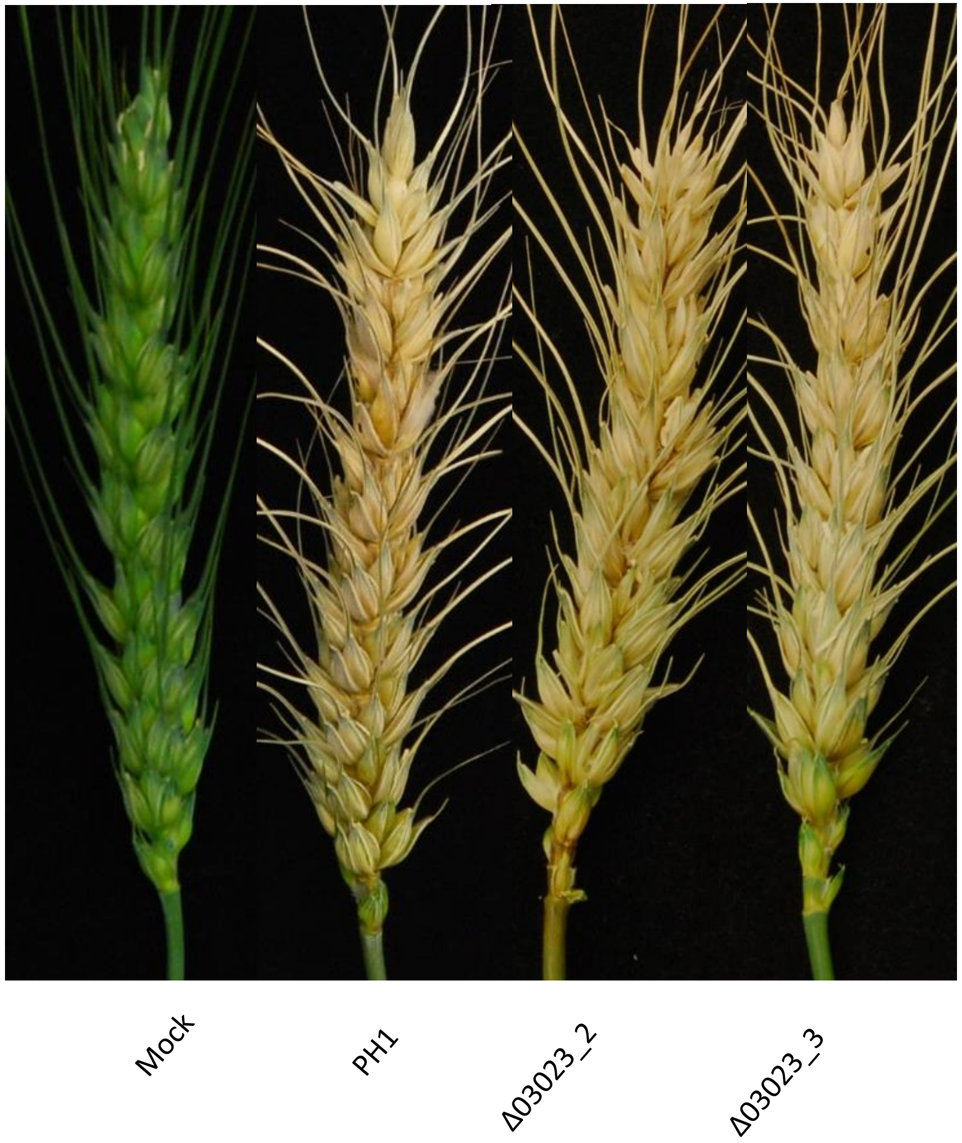

Class V: FGRRES_05239

Split marker mediated gene replacement

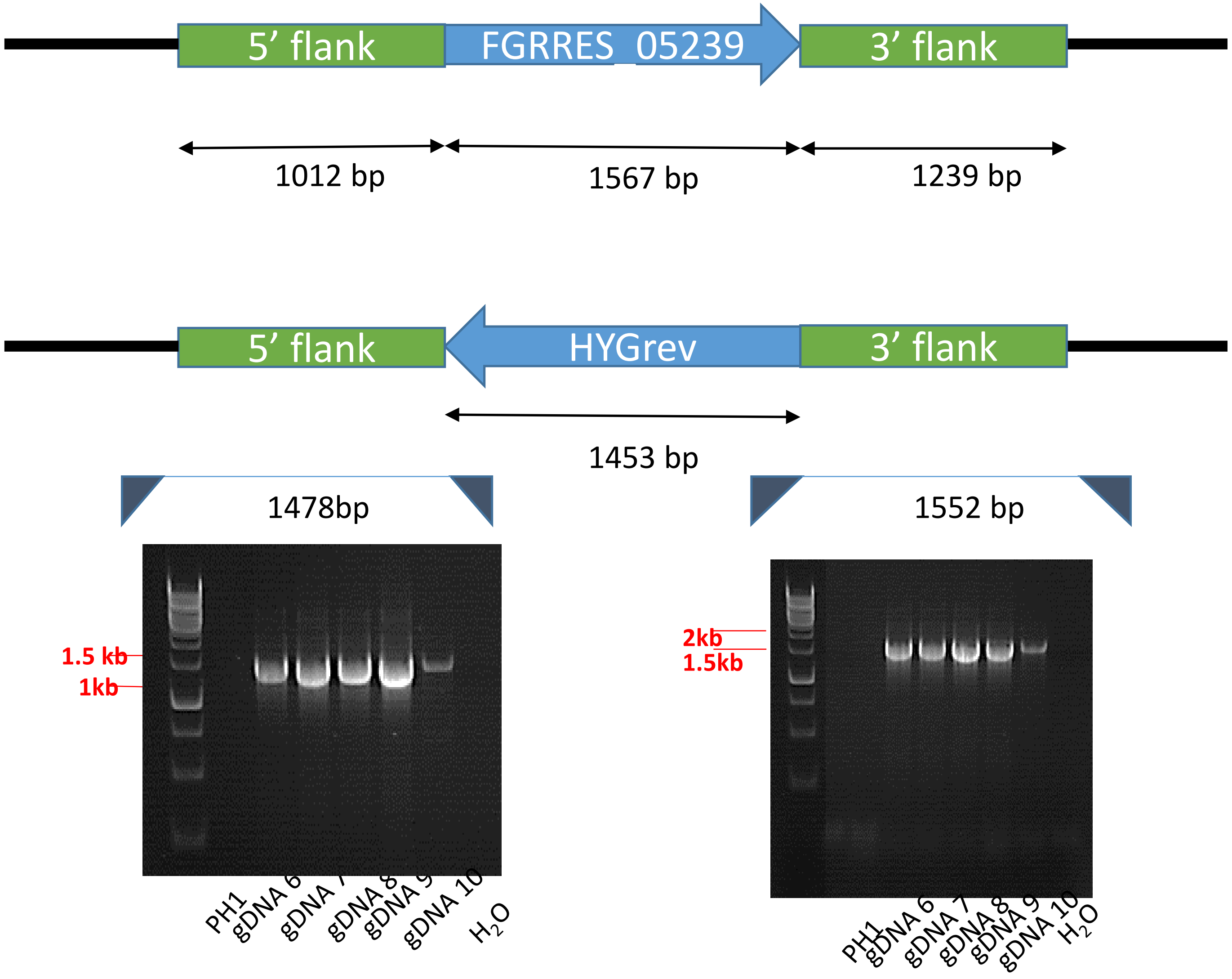

FHB symptoms on wheat 15 days post infection

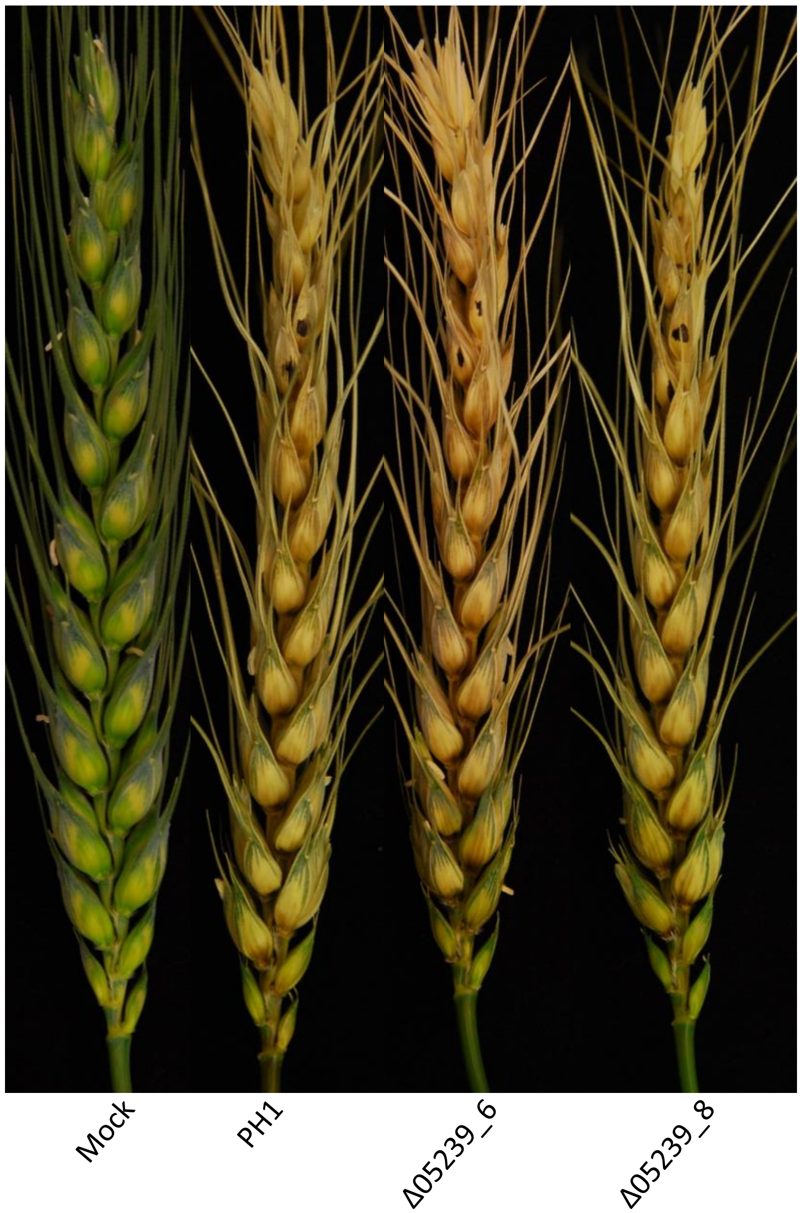

Class V: FGRRES_17256

Split marker mediated gene replacement

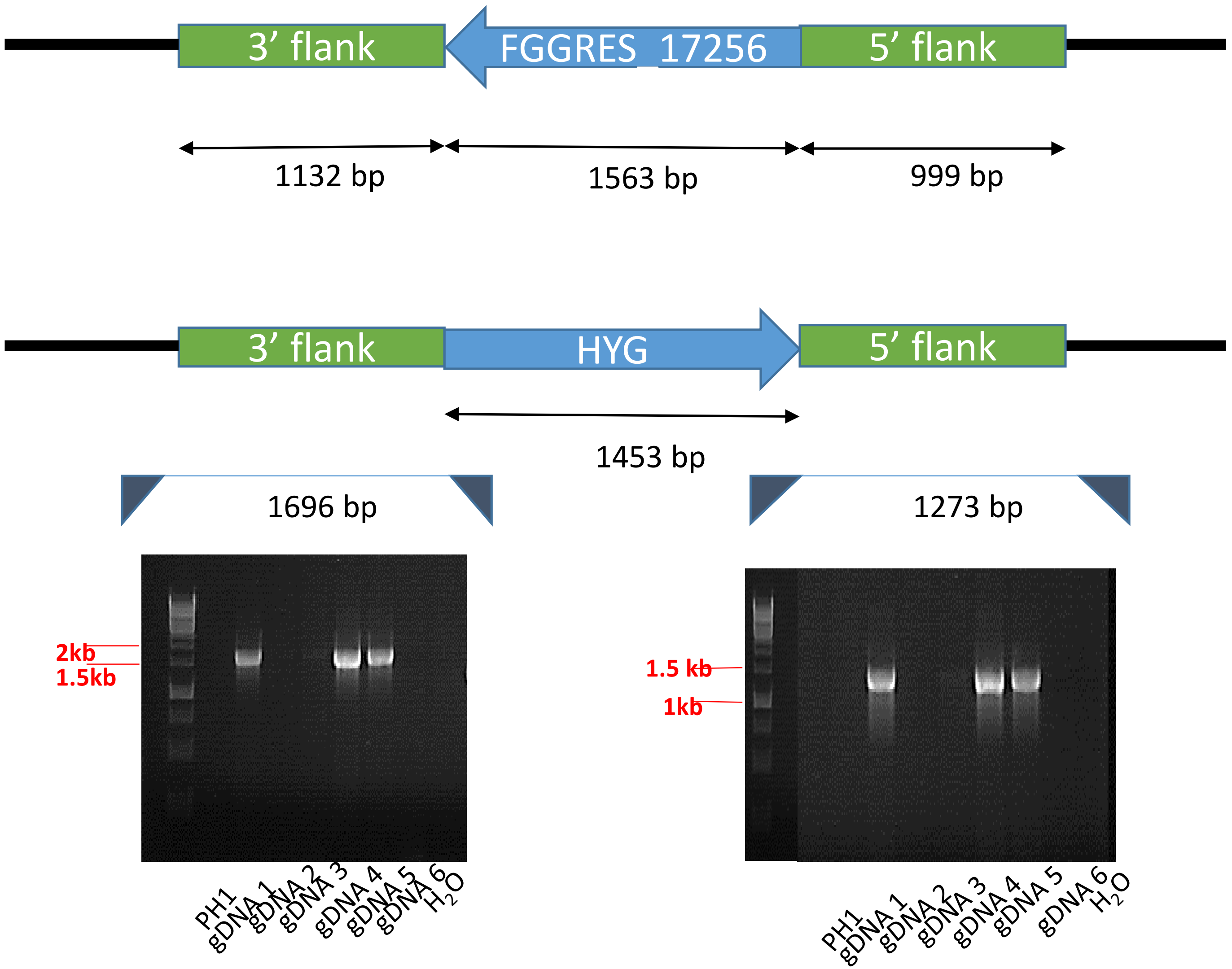

FHB symptoms on wheat 15 days post infection

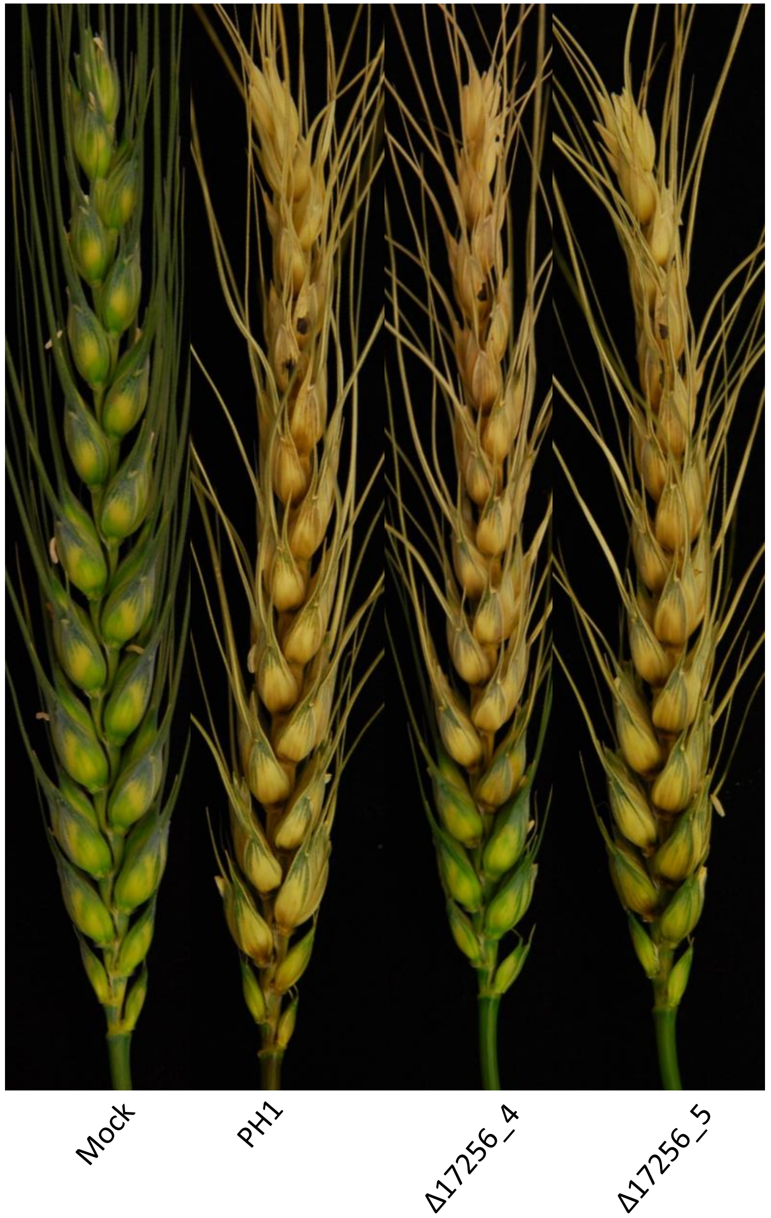

Class X: FGRRES_00994

Split marker mediated gene replacement

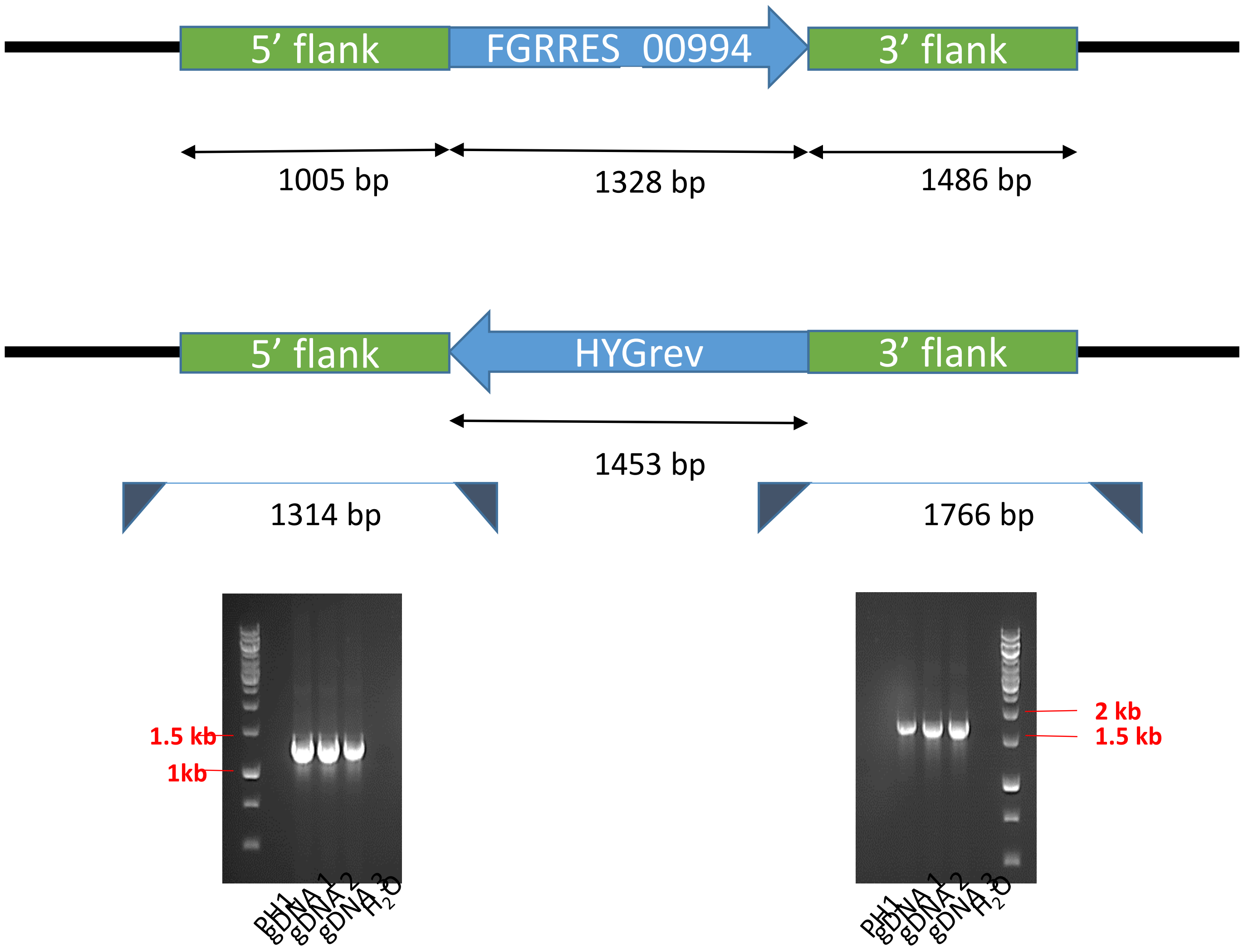

FHB symptoms on wheat 15 days post infection

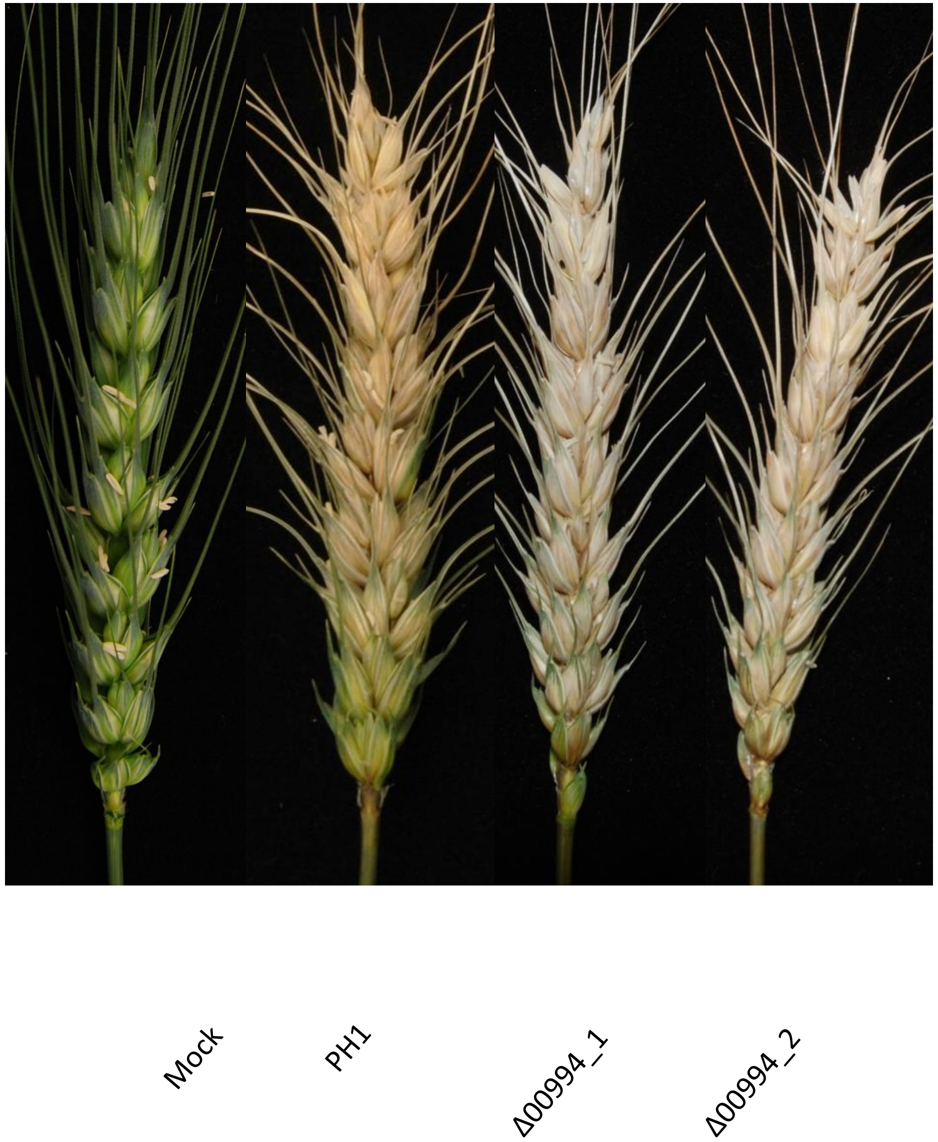

Class X: FGRRES_ 01833

Split marker mediated gene replacement

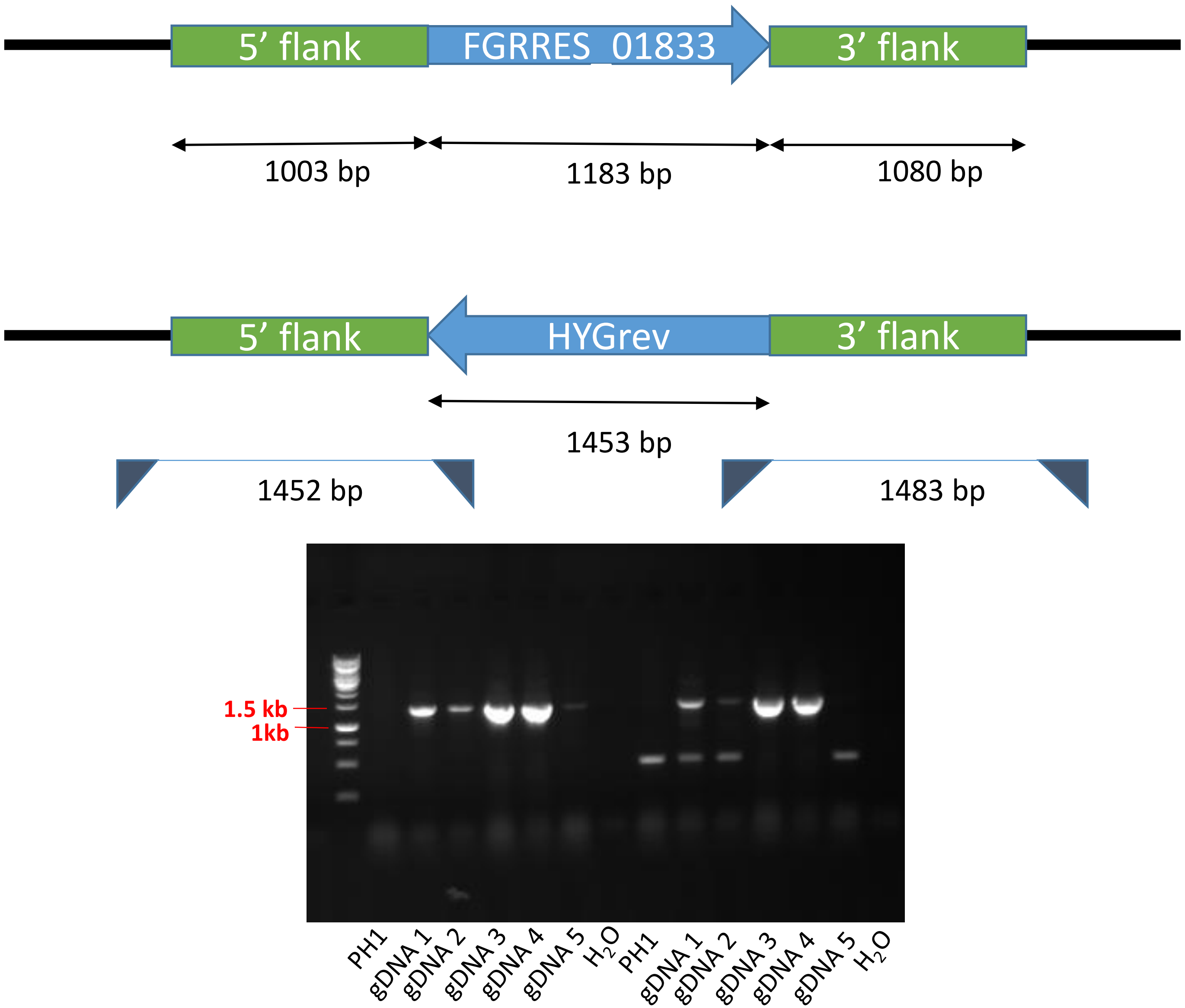

FHB symptoms on wheat 15 days post infection

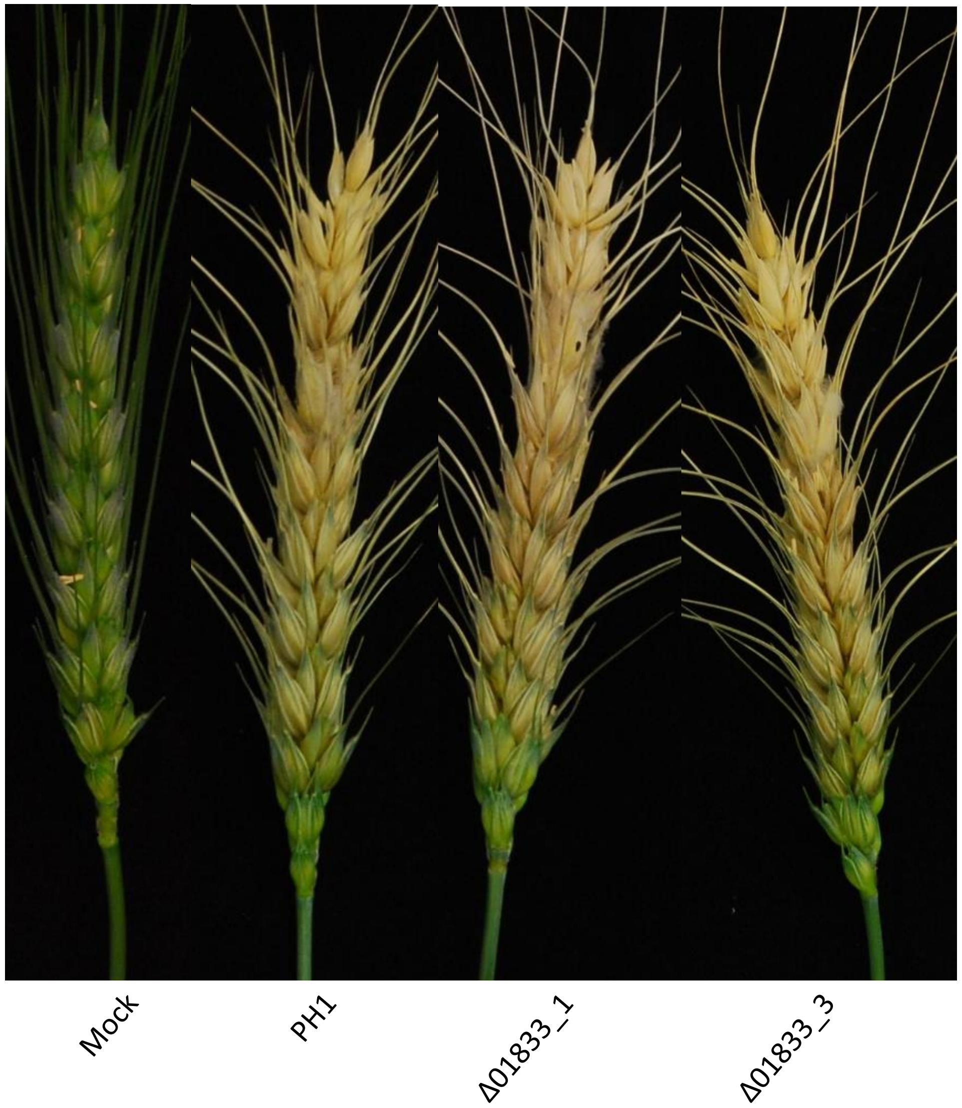

Class X: FGRRES_ 02401

Split marker mediated gene replacement

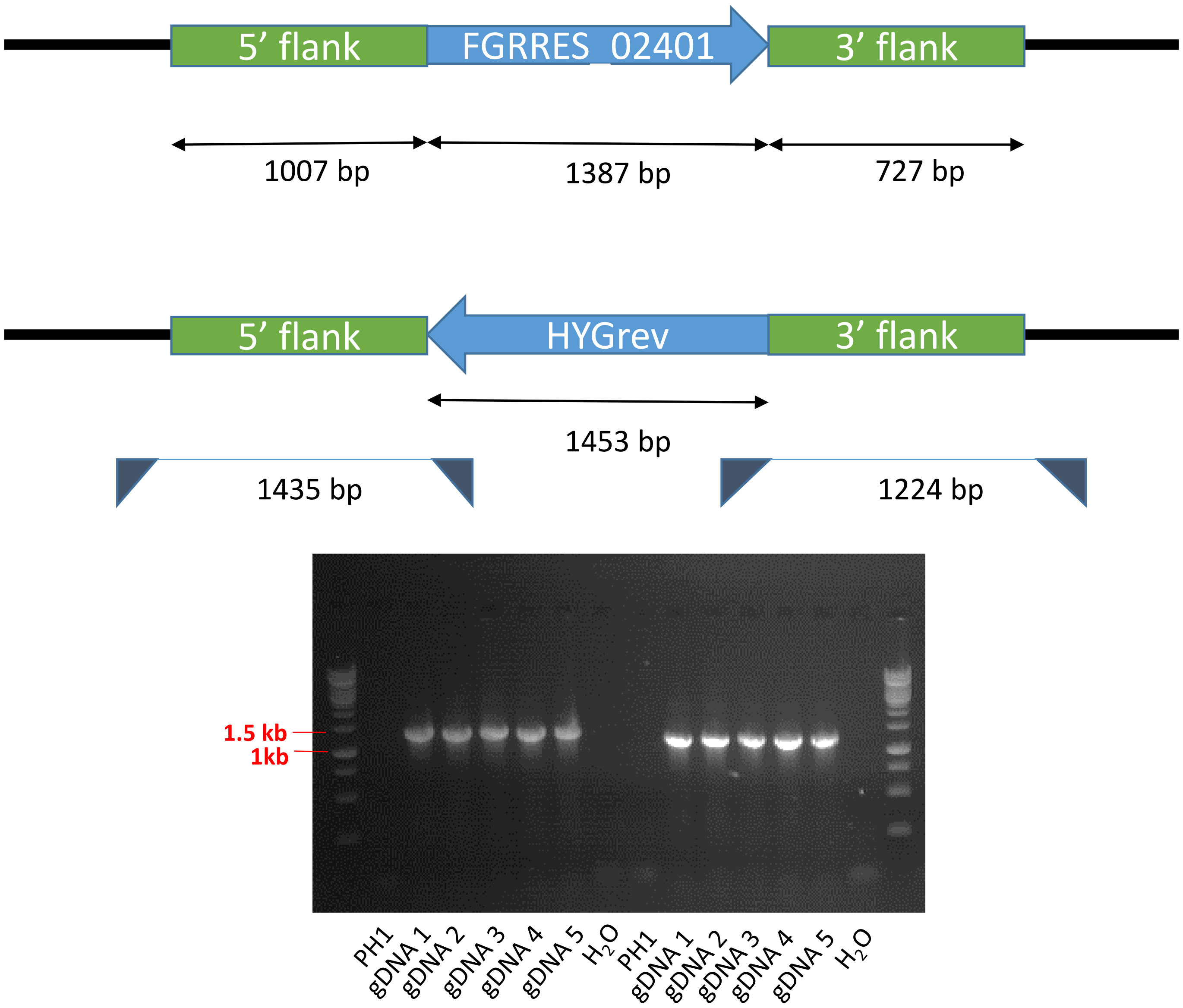

FHB symptoms on wheat 15 days post infection

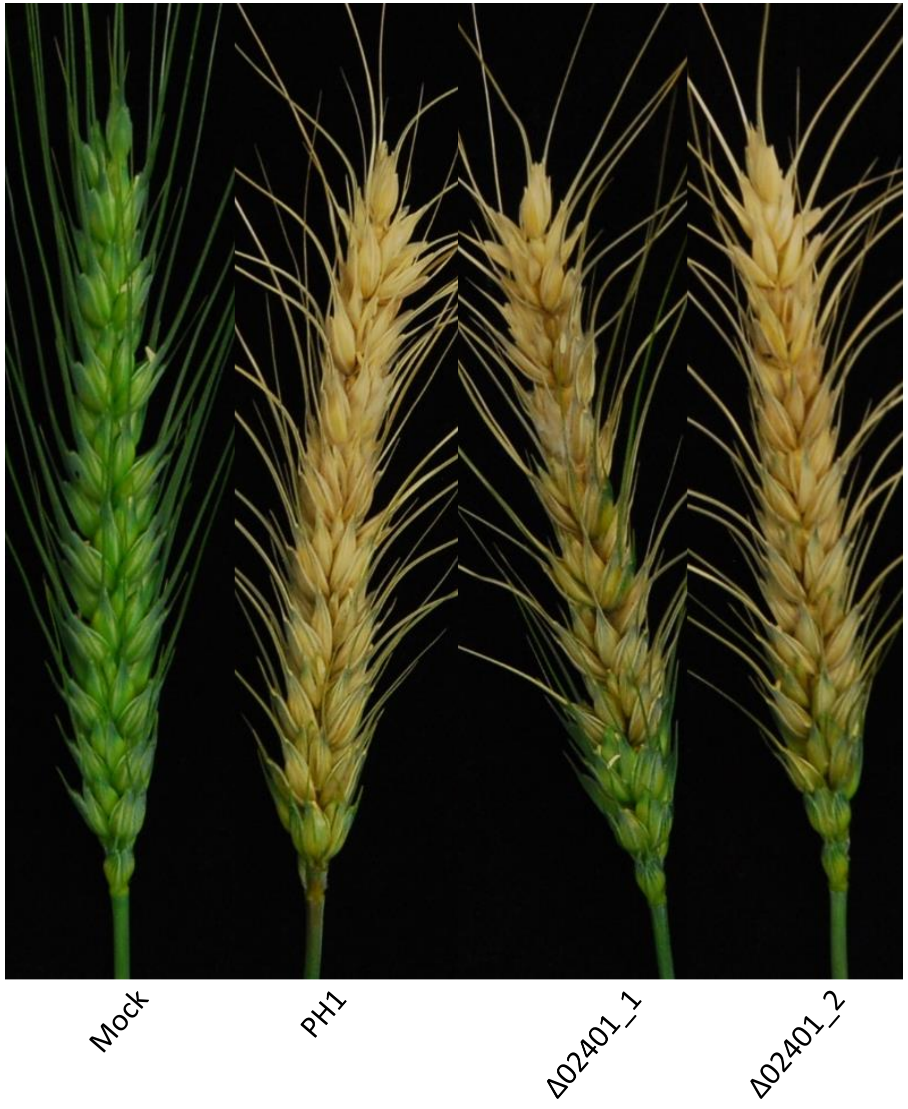

Class X: FGRRES_ 03151

Split marker mediated gene replacement

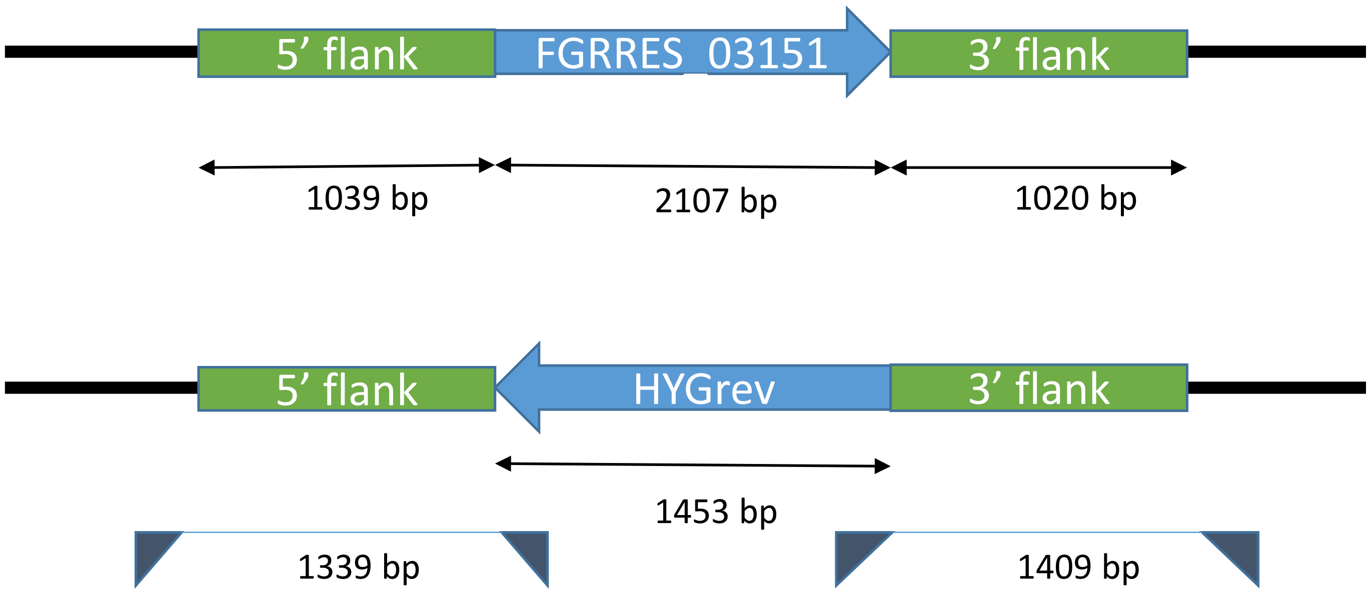

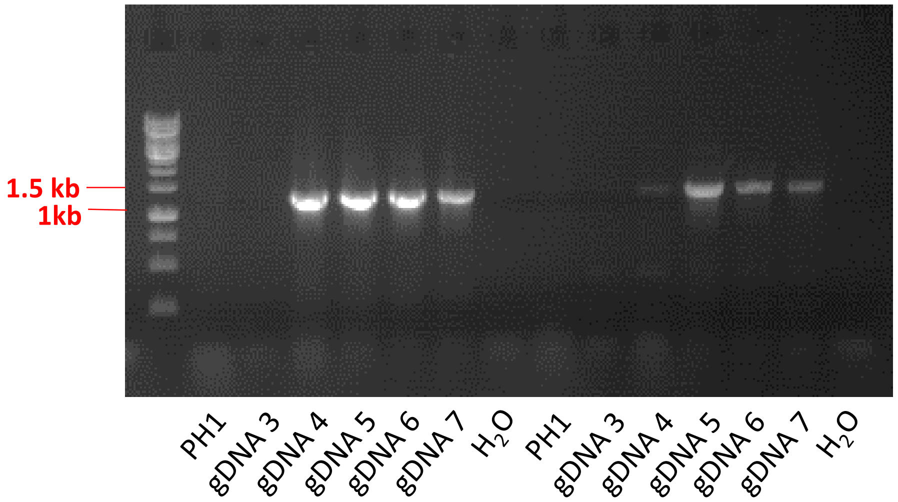

FHB symptoms on wheat 15 days post infection

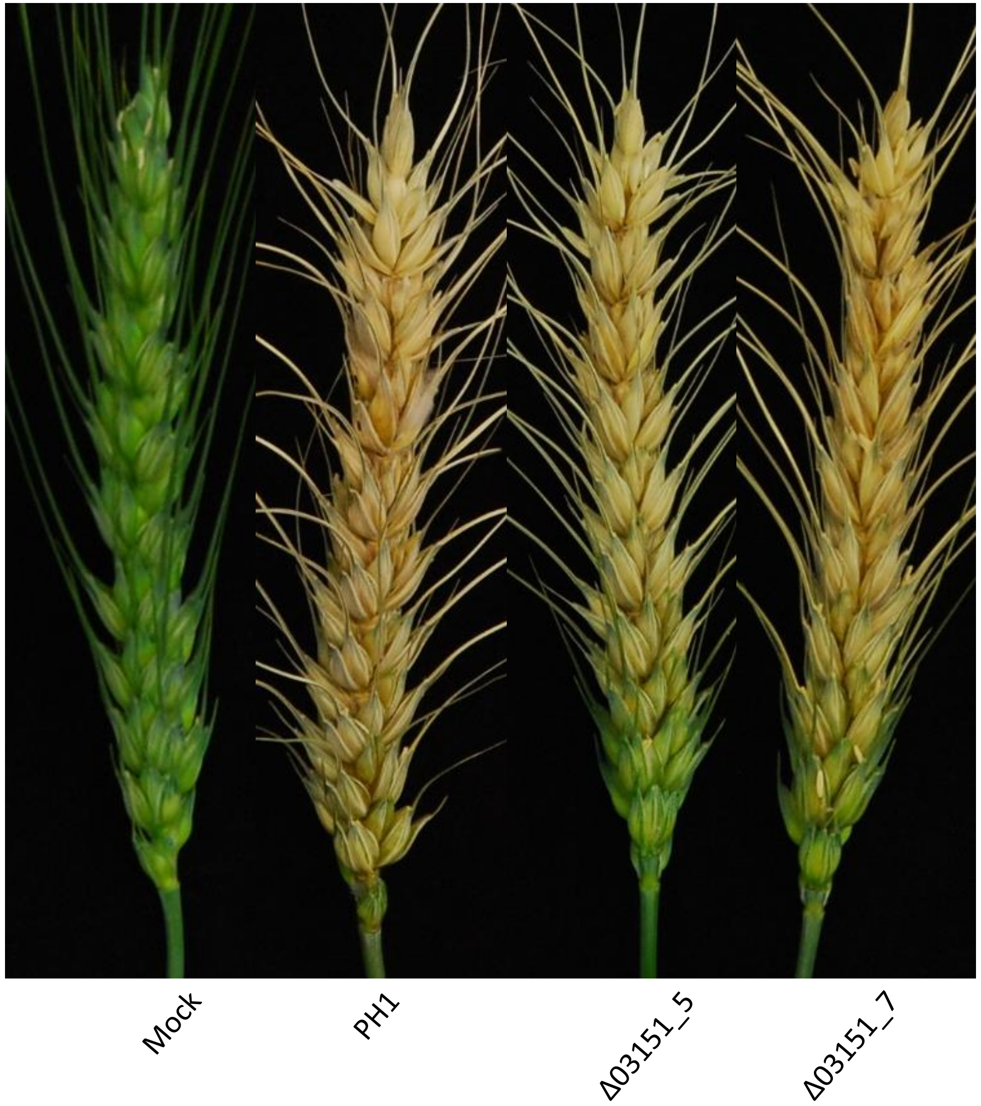

Class X: FGRRES_ 03800

Split marker mediated gene replacement

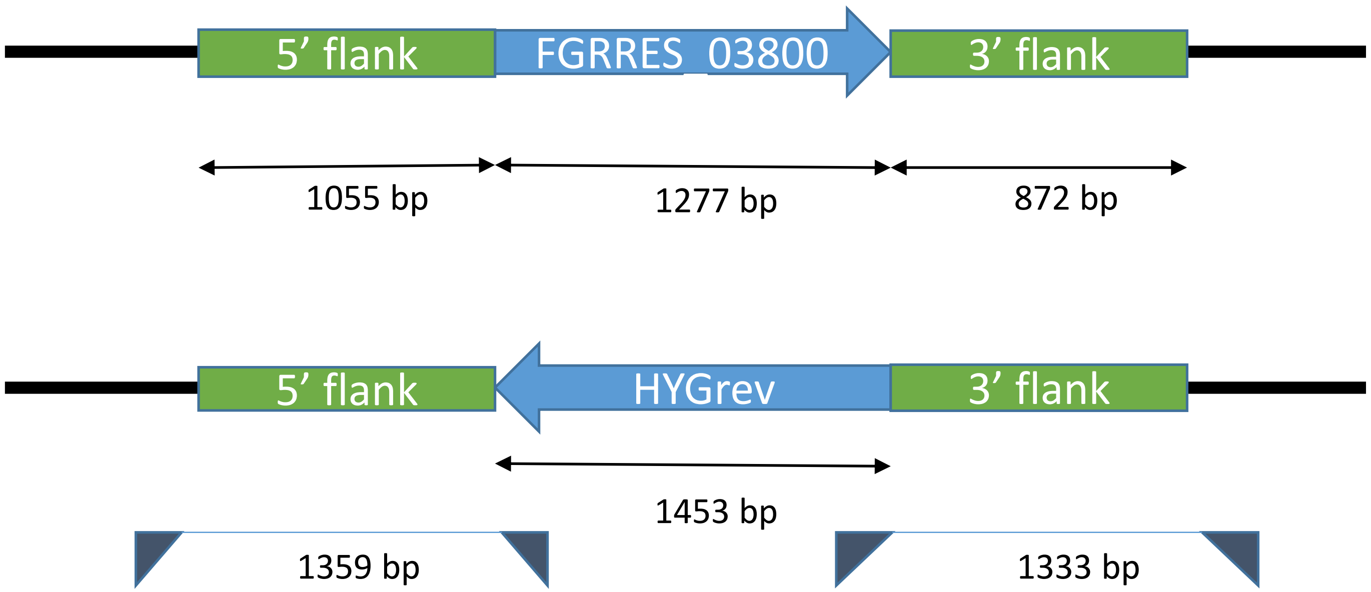

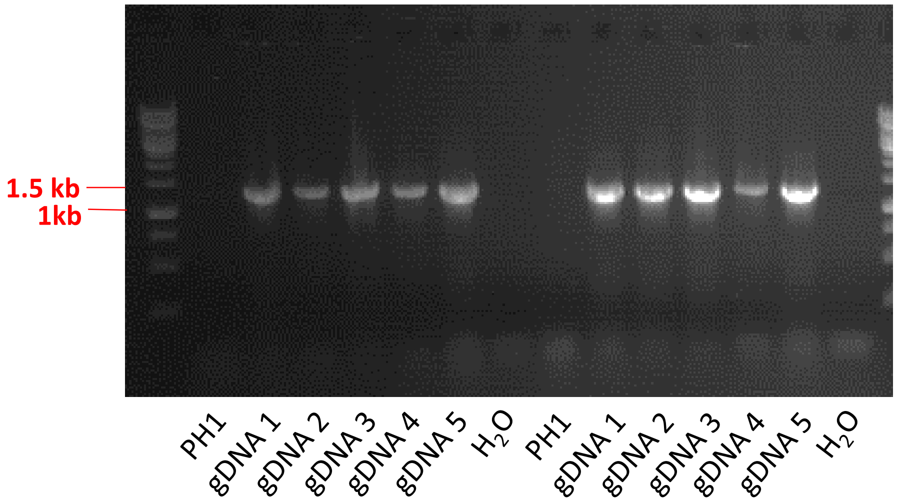

FHB symptoms on wheat 15 days post infection

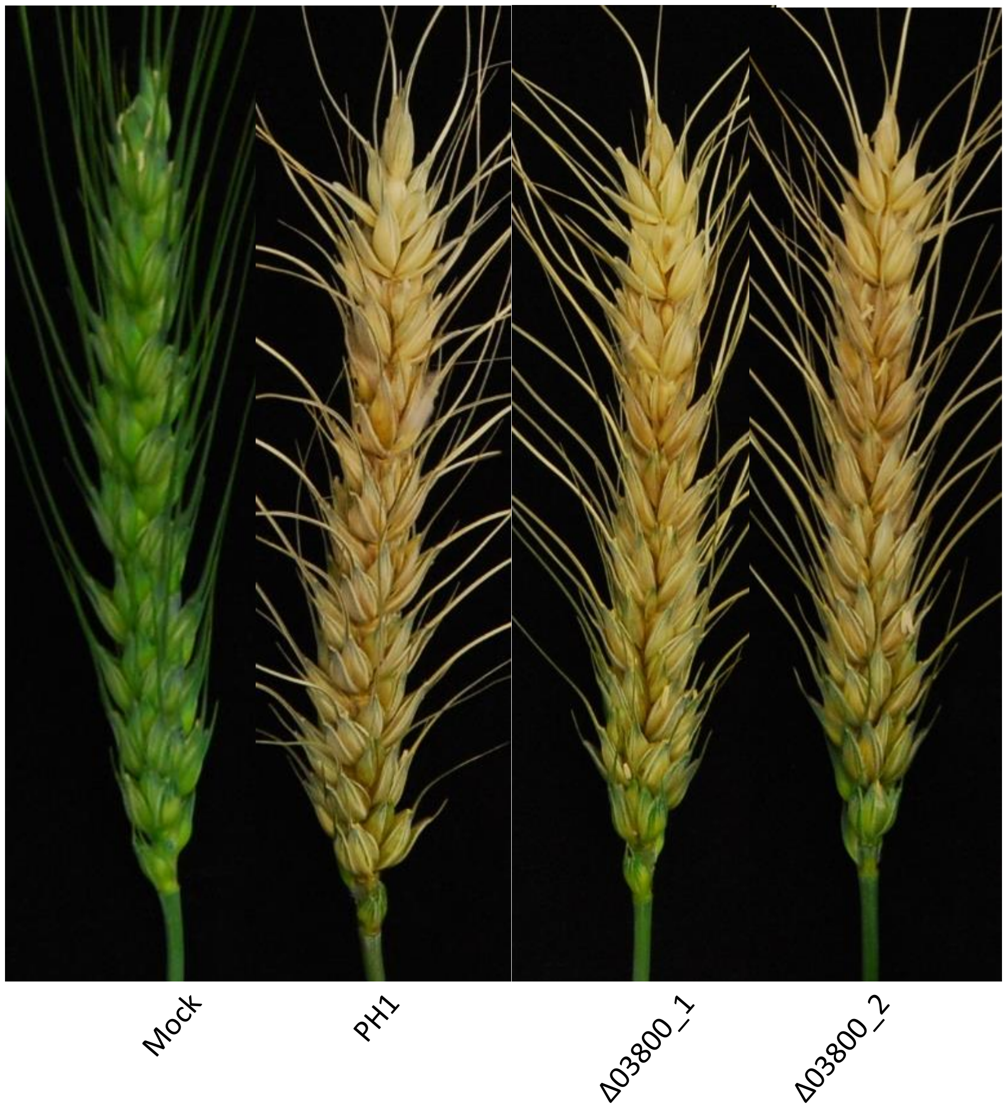

Class X: FGRRES_ 04693

Split marker mediated gene replacement

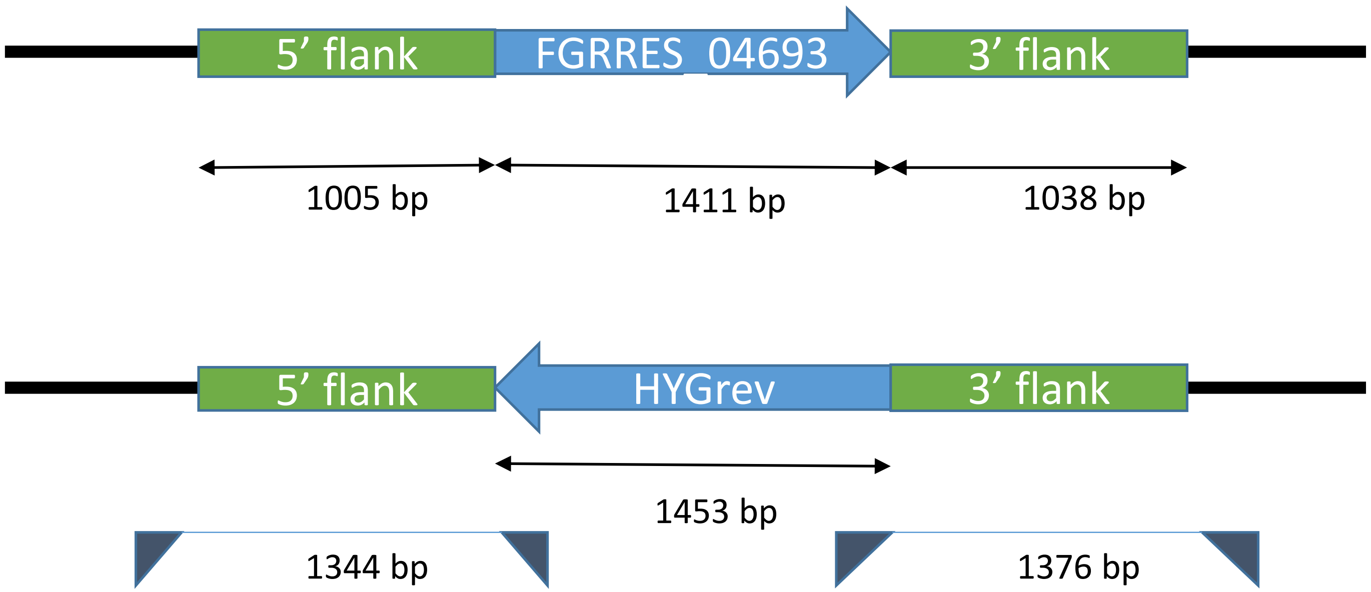

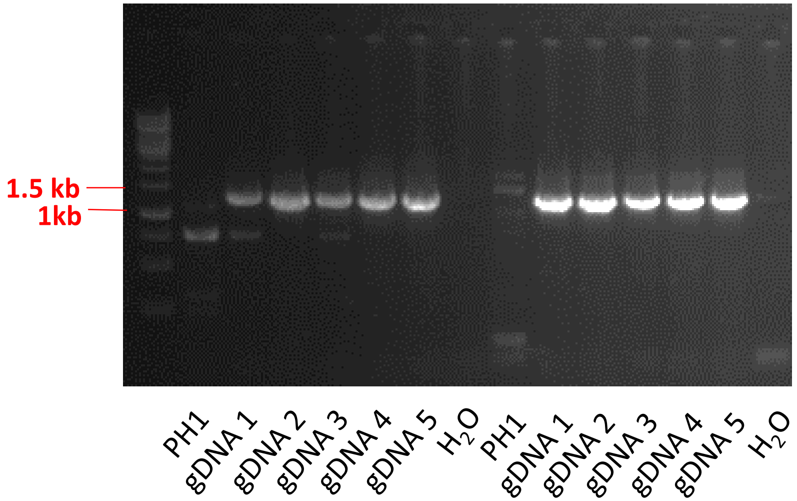

FHB symptoms on wheat 15 days post infection

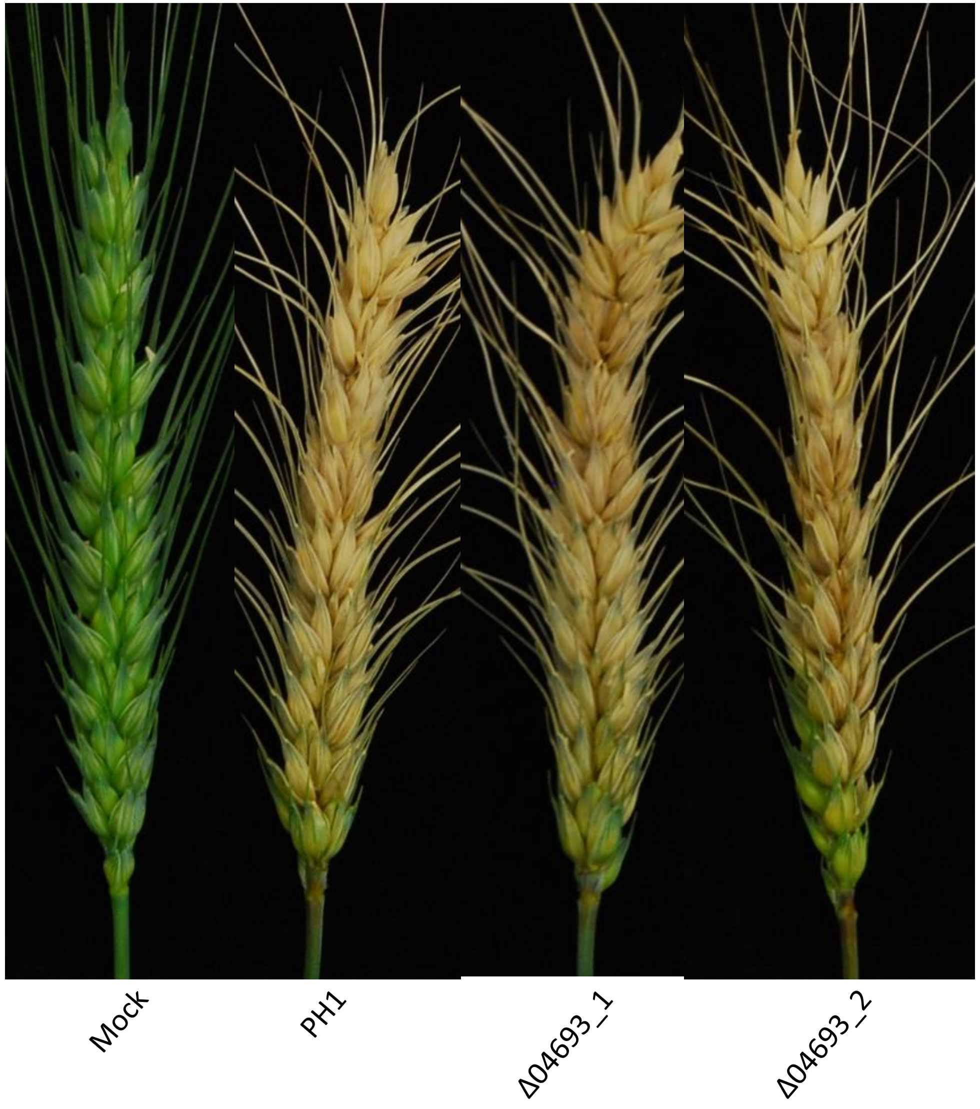

Class X: FGRRES_ 04731

Split marker mediated gene replacement

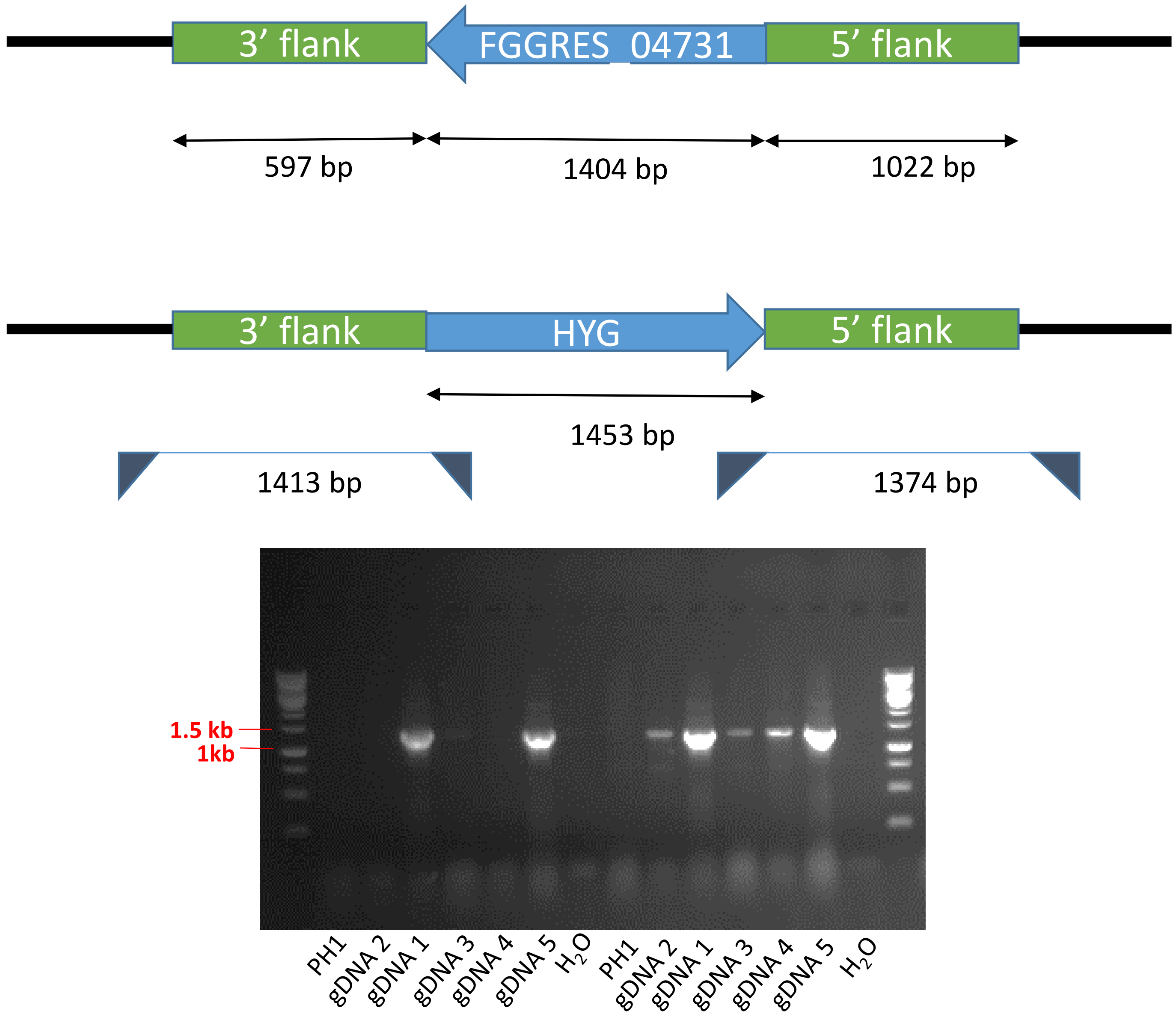

FHB symptoms on wheat 15 days post infection

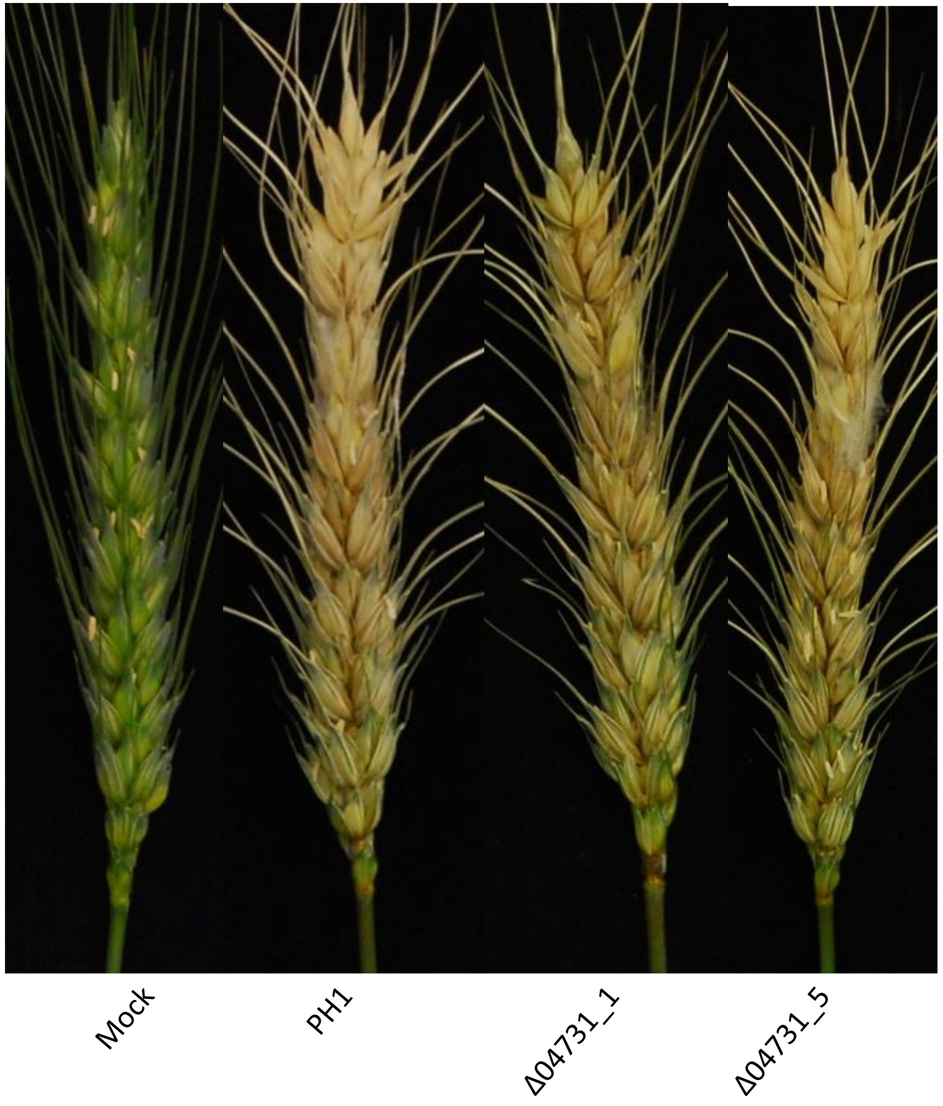

Class X: FGRRES_ 06541

Split marker mediated gene replacement

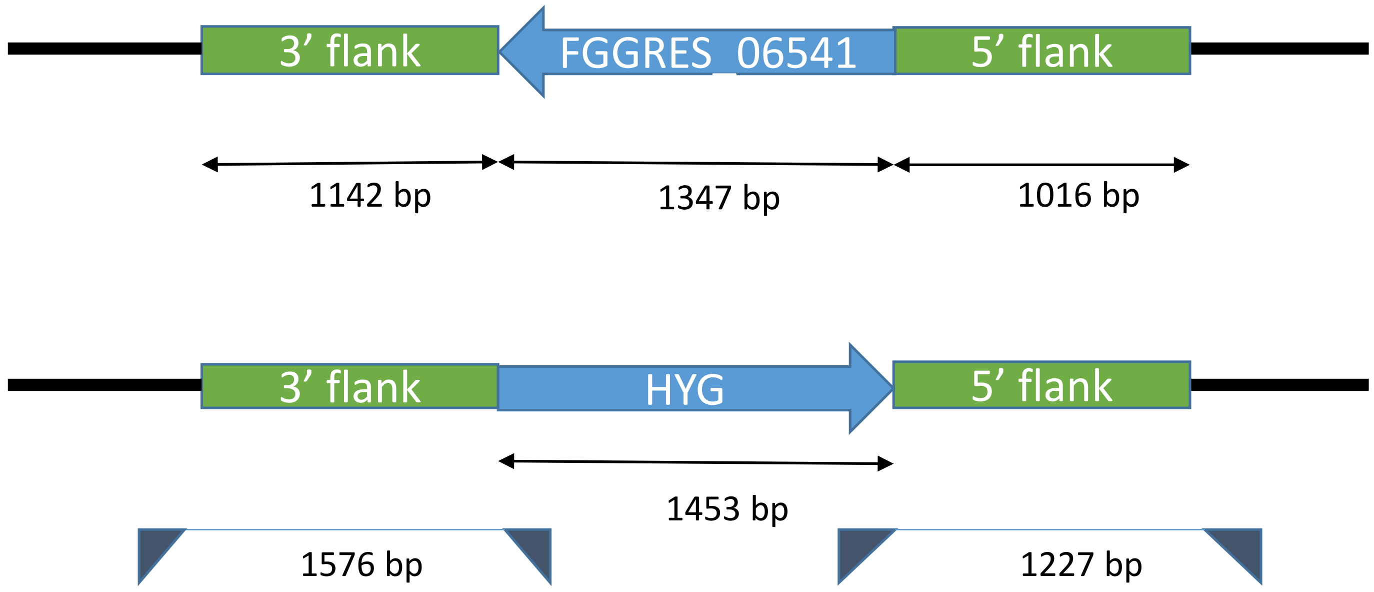

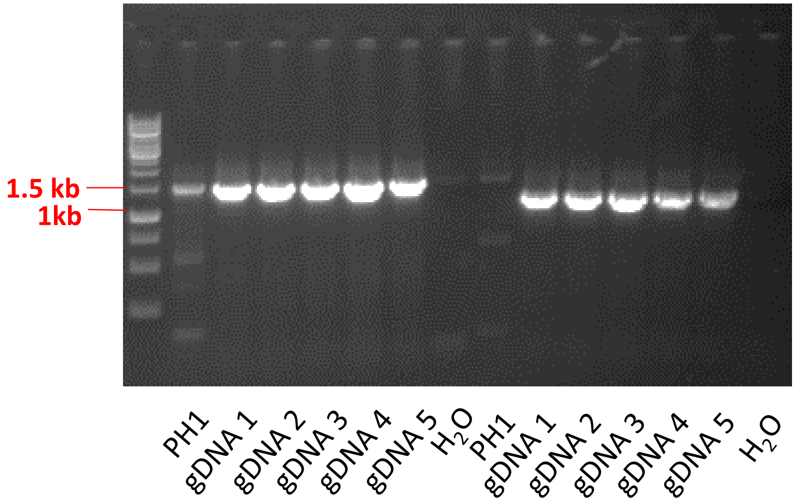

FHB symptoms on wheat 15 days post infection

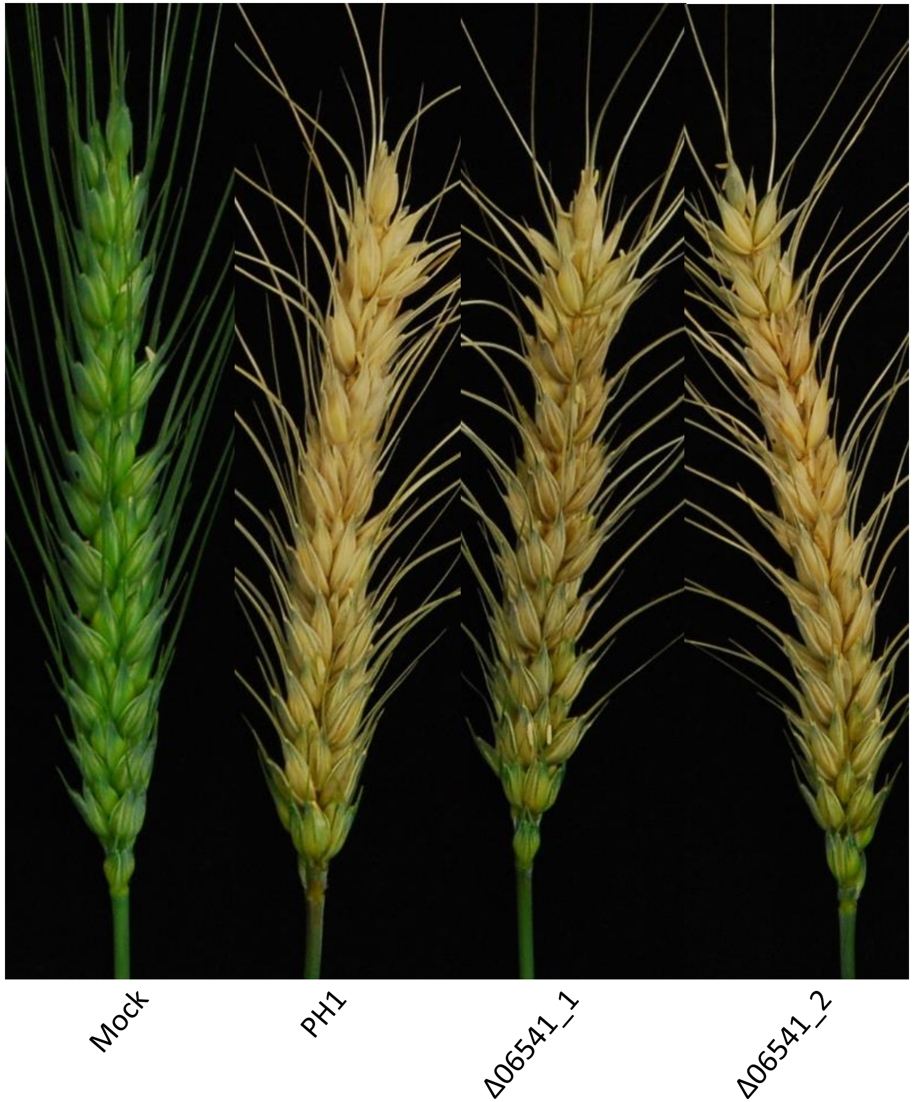

Class X: FGRRES_ 07655

Split marker mediated gene replacement

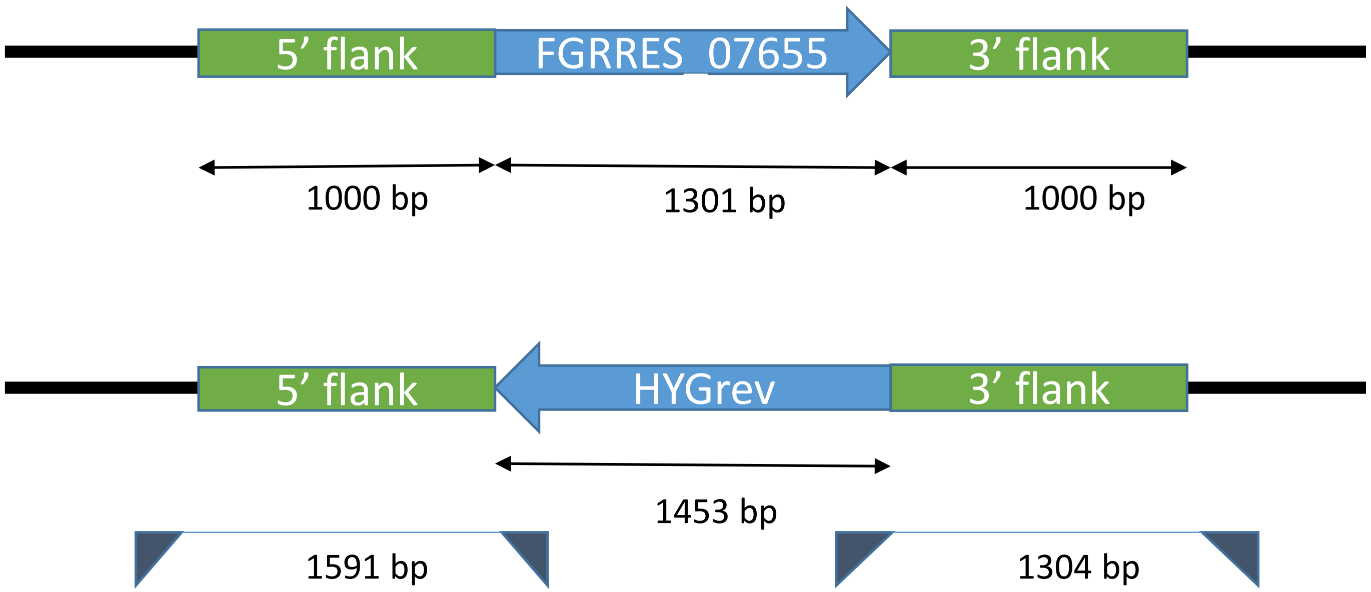

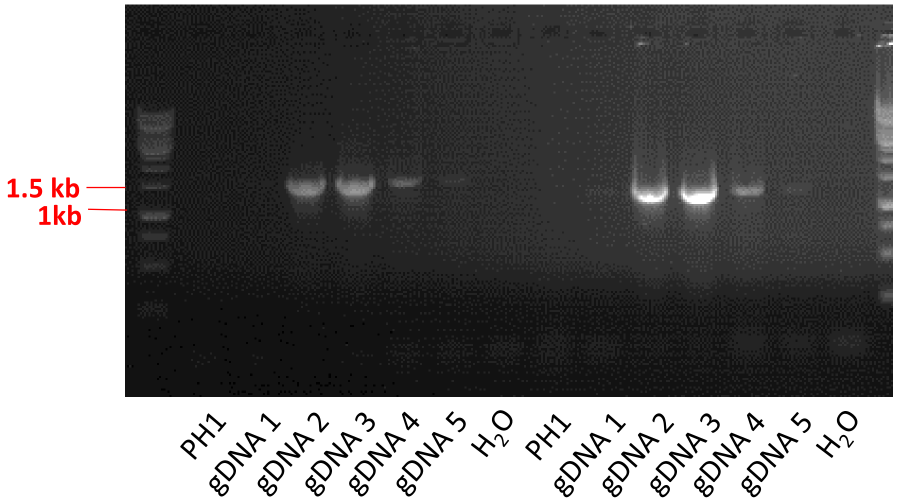

FHB symptoms on wheat 15 days post infection

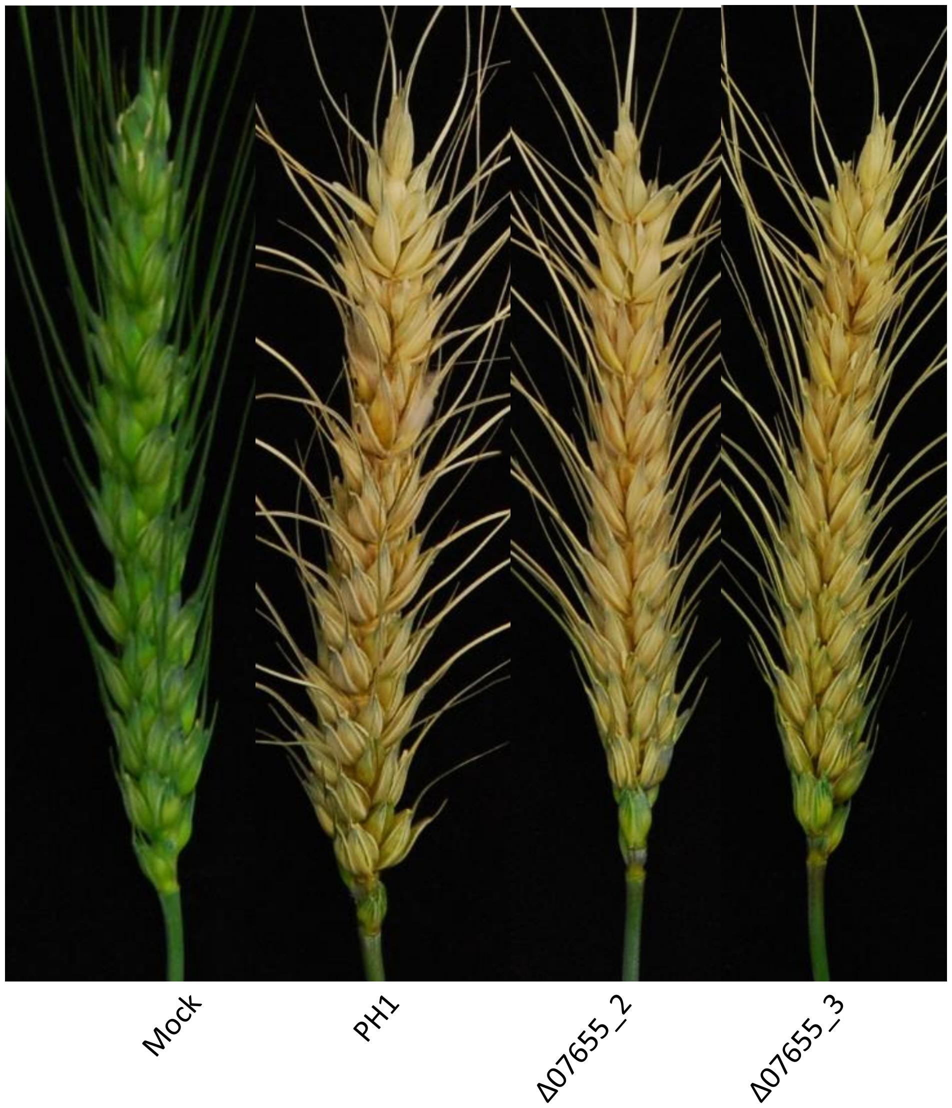

Class X: FGRRES_ 07792

Split marker mediated gene replacement

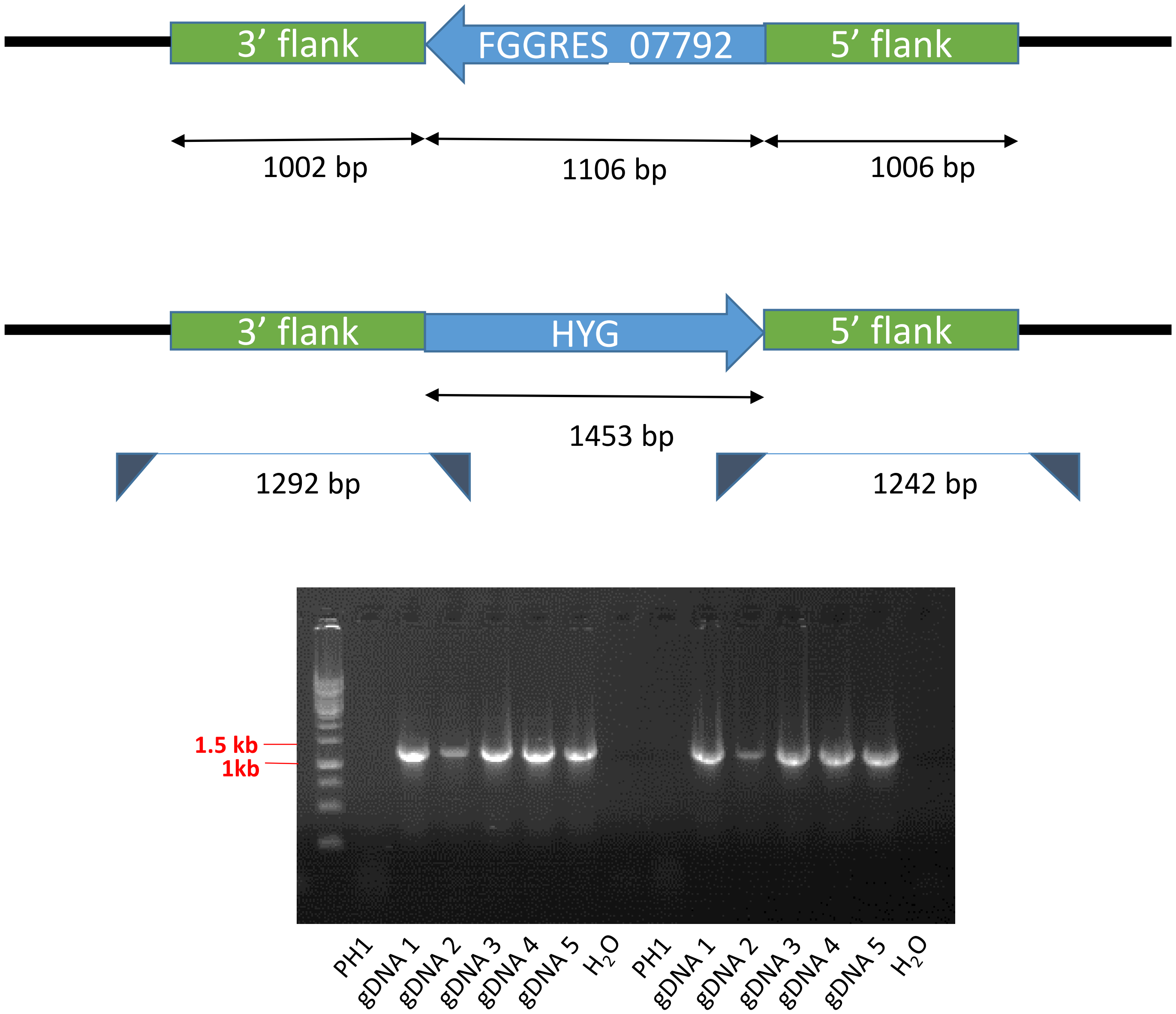

FHB symptoms on wheat 15 days post infection

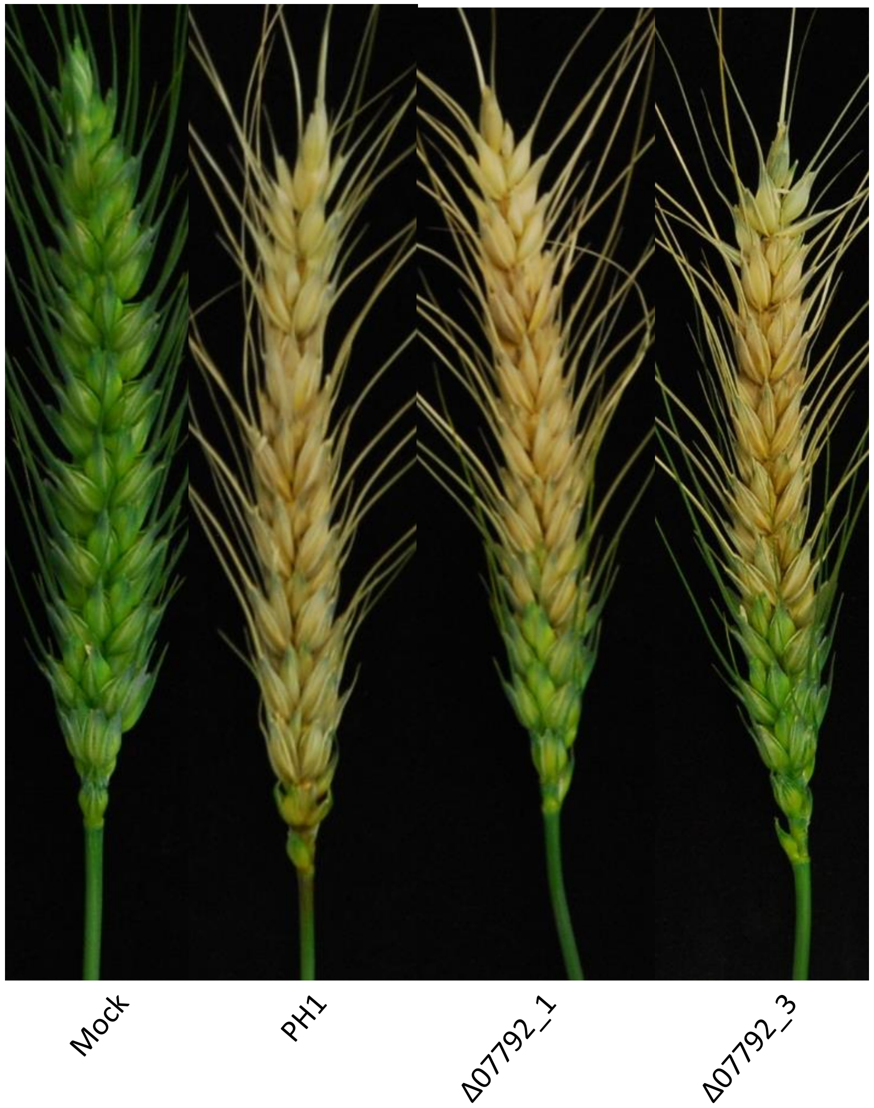

Class X: FGRRES_ 10598

Split marker mediated gene replacement

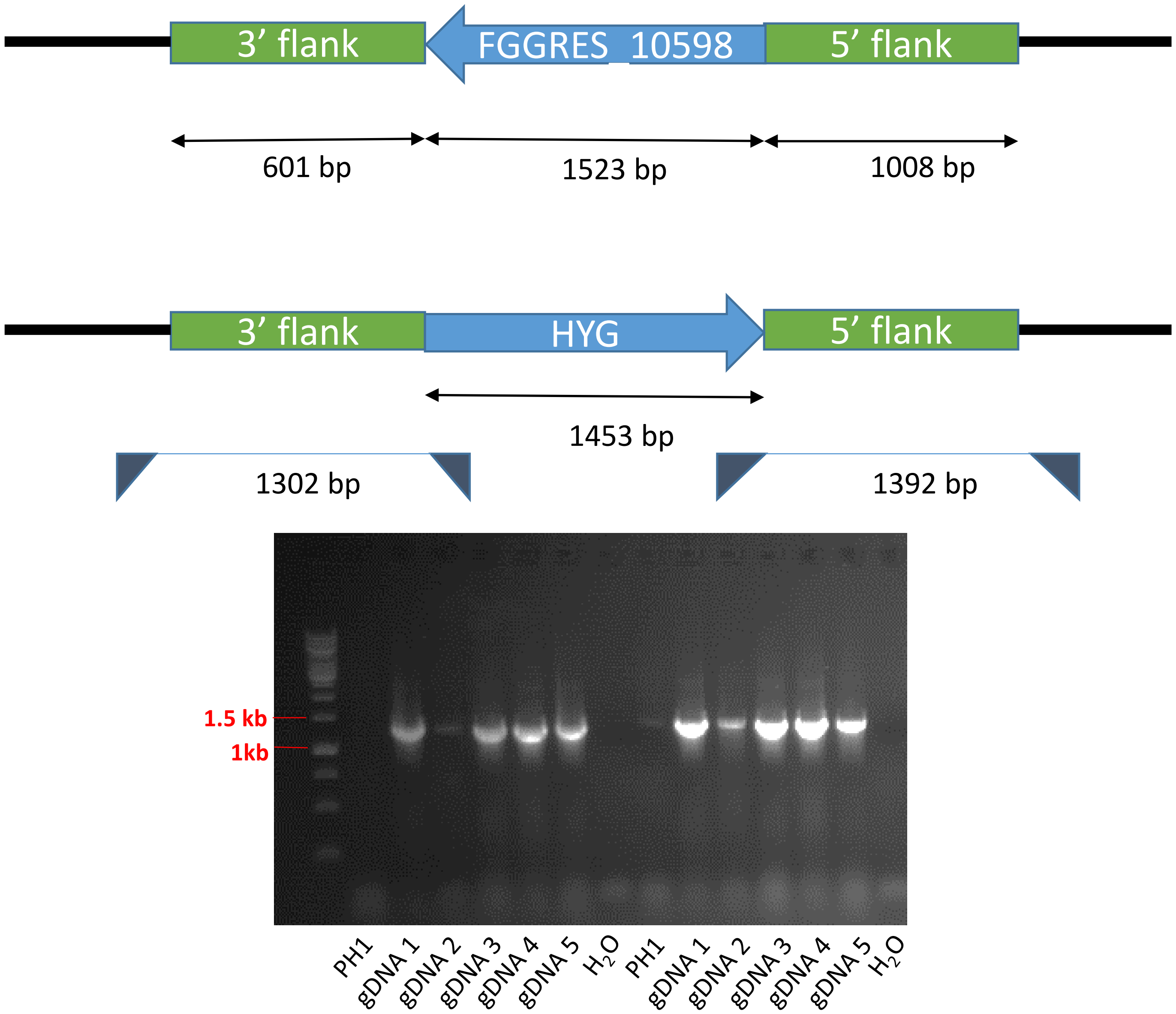

FHB symptoms on wheat 15 days post infection

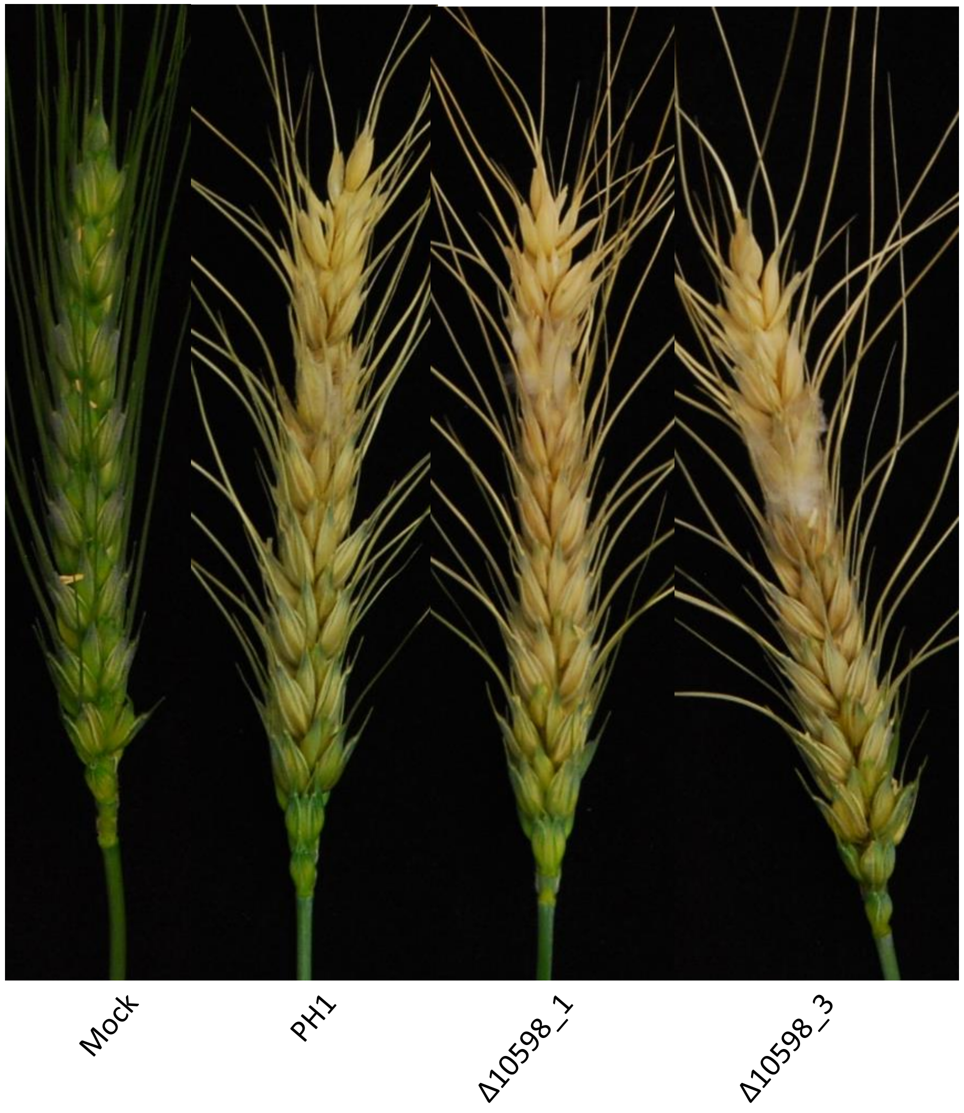

Class X: FGRRES_ 15938

Split marker mediated gene replacement

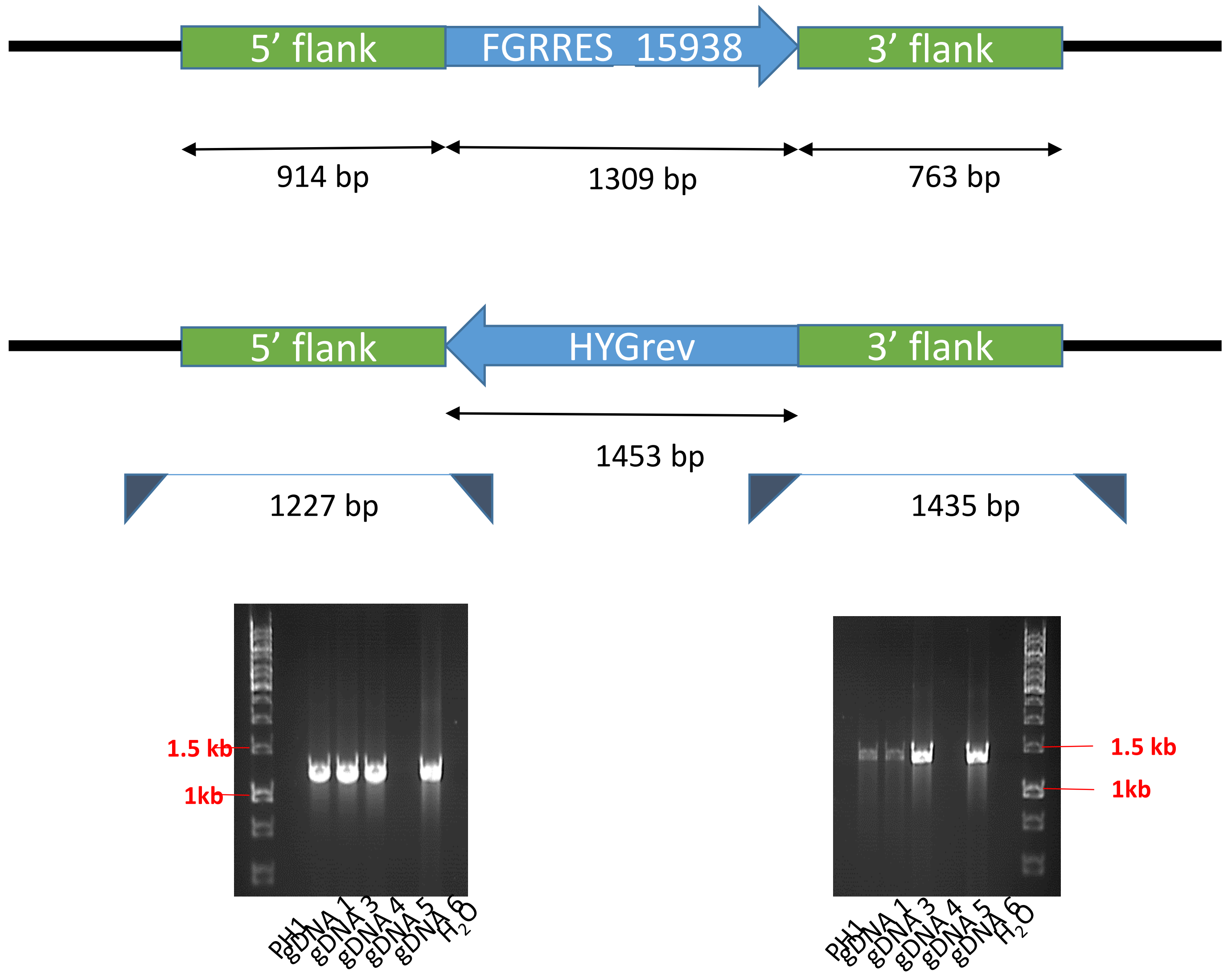

FHB symptoms on wheat 15 days post infection

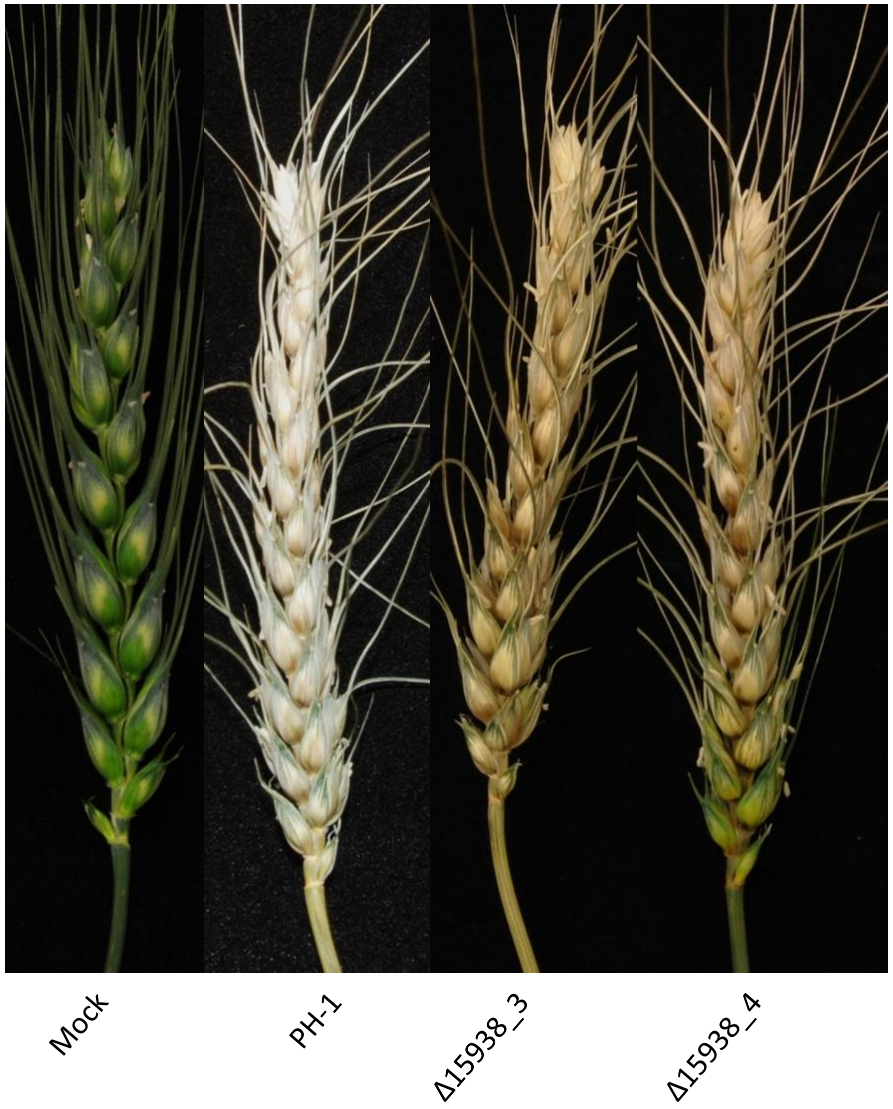

Class X: FGRRES_ 16282

Split marker mediated gene replacement

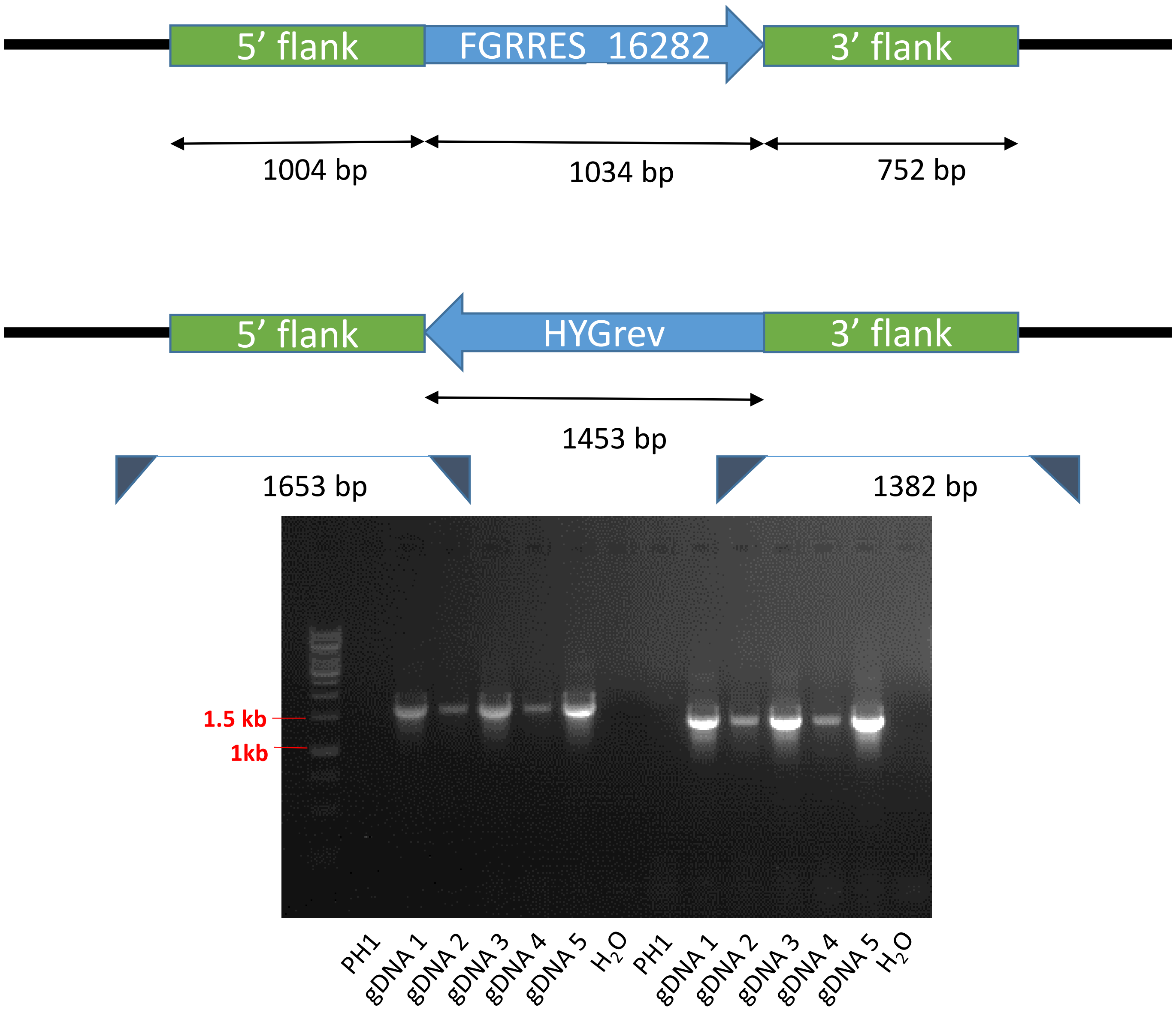

FHB symptoms on wheat 15 days post infection

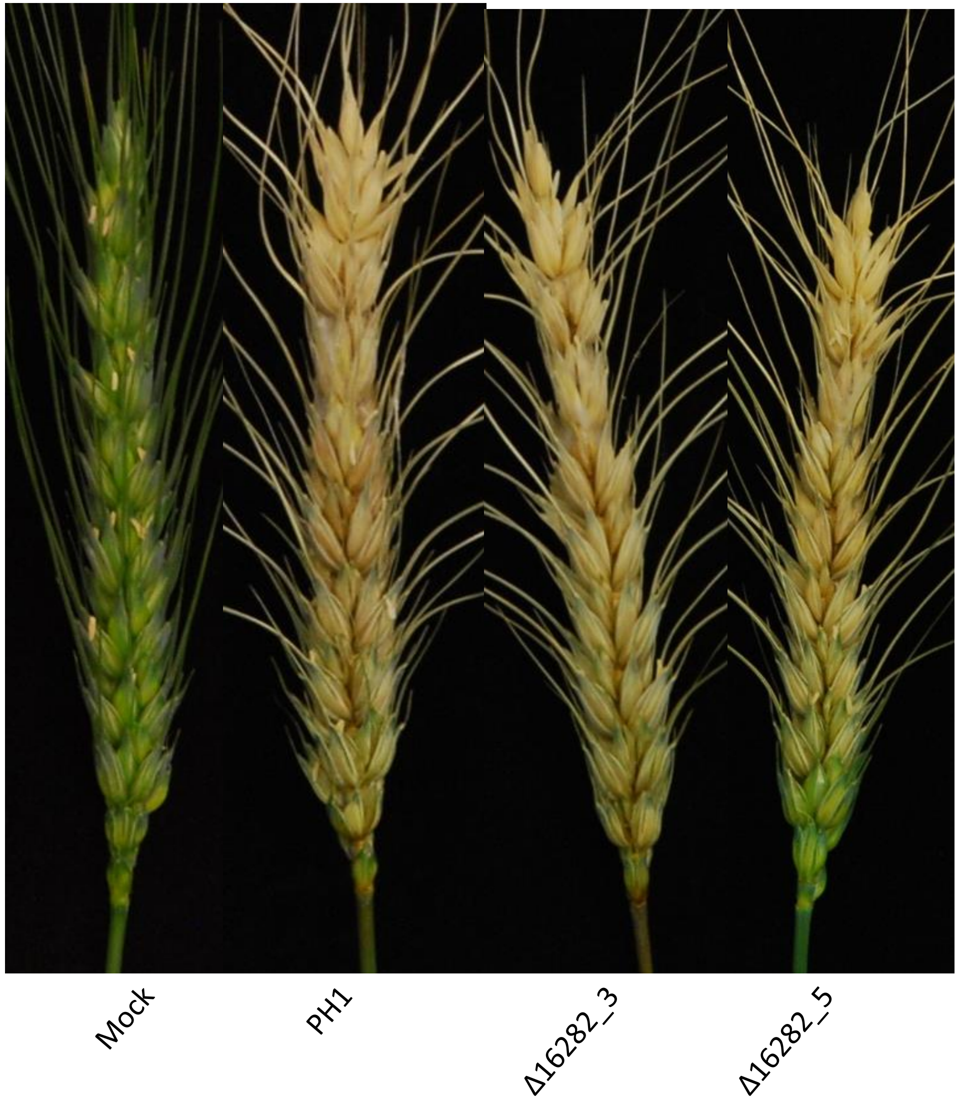

Class X: FGRRES_ 16354

Split marker mediated gene replacement

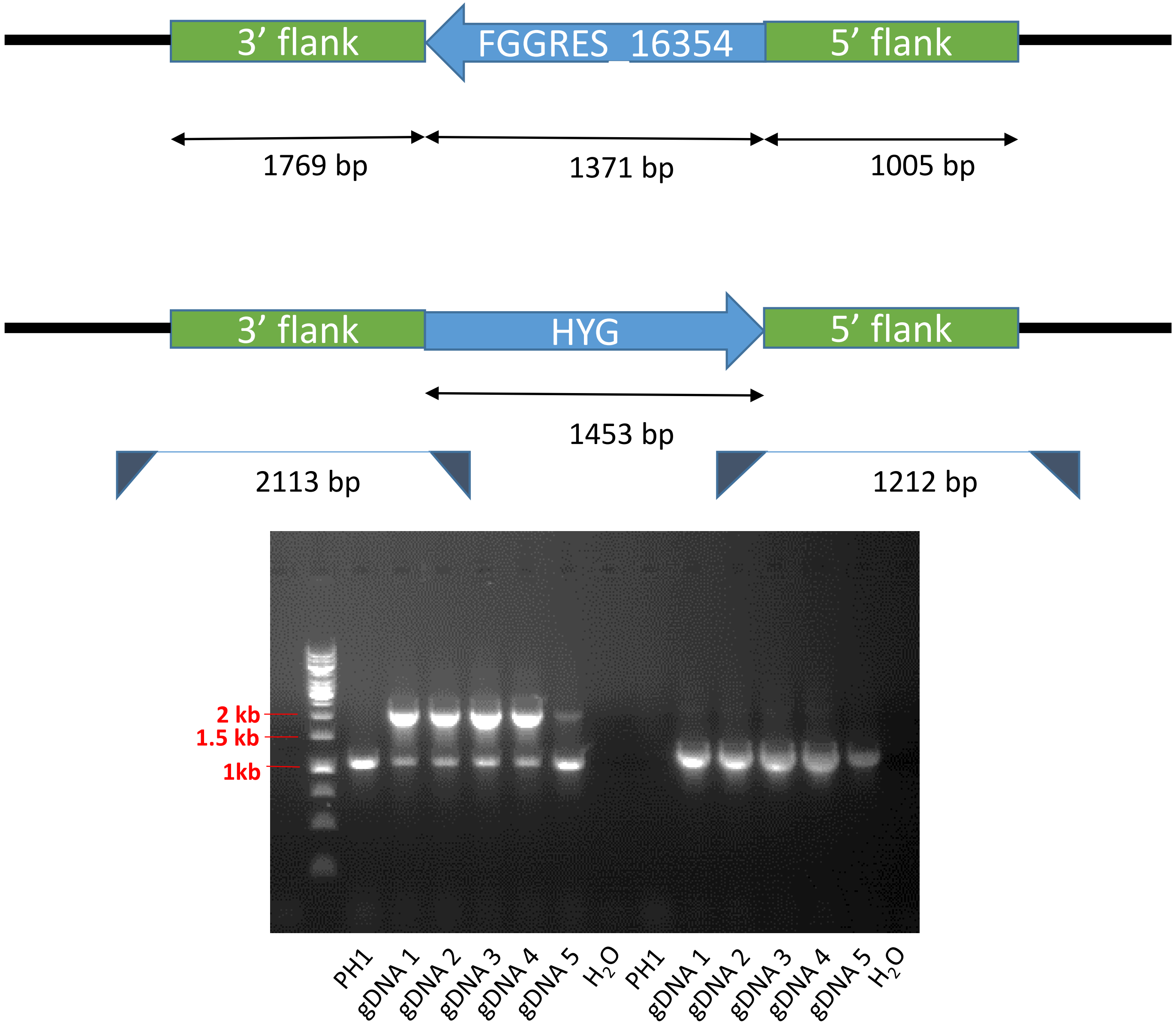

FHB symptoms on wheat 15 days post infection

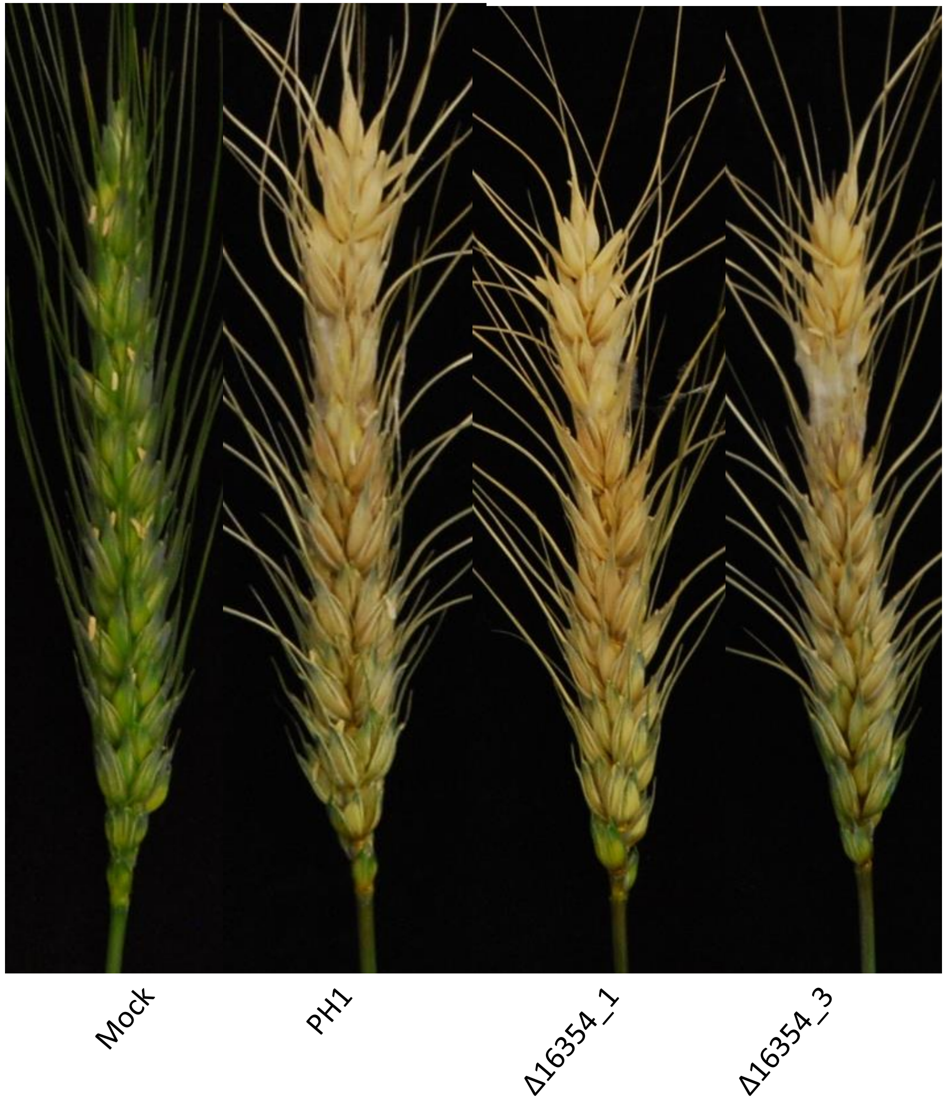

Class X: FGRRES_ 16358

Split marker mediated gene replacement

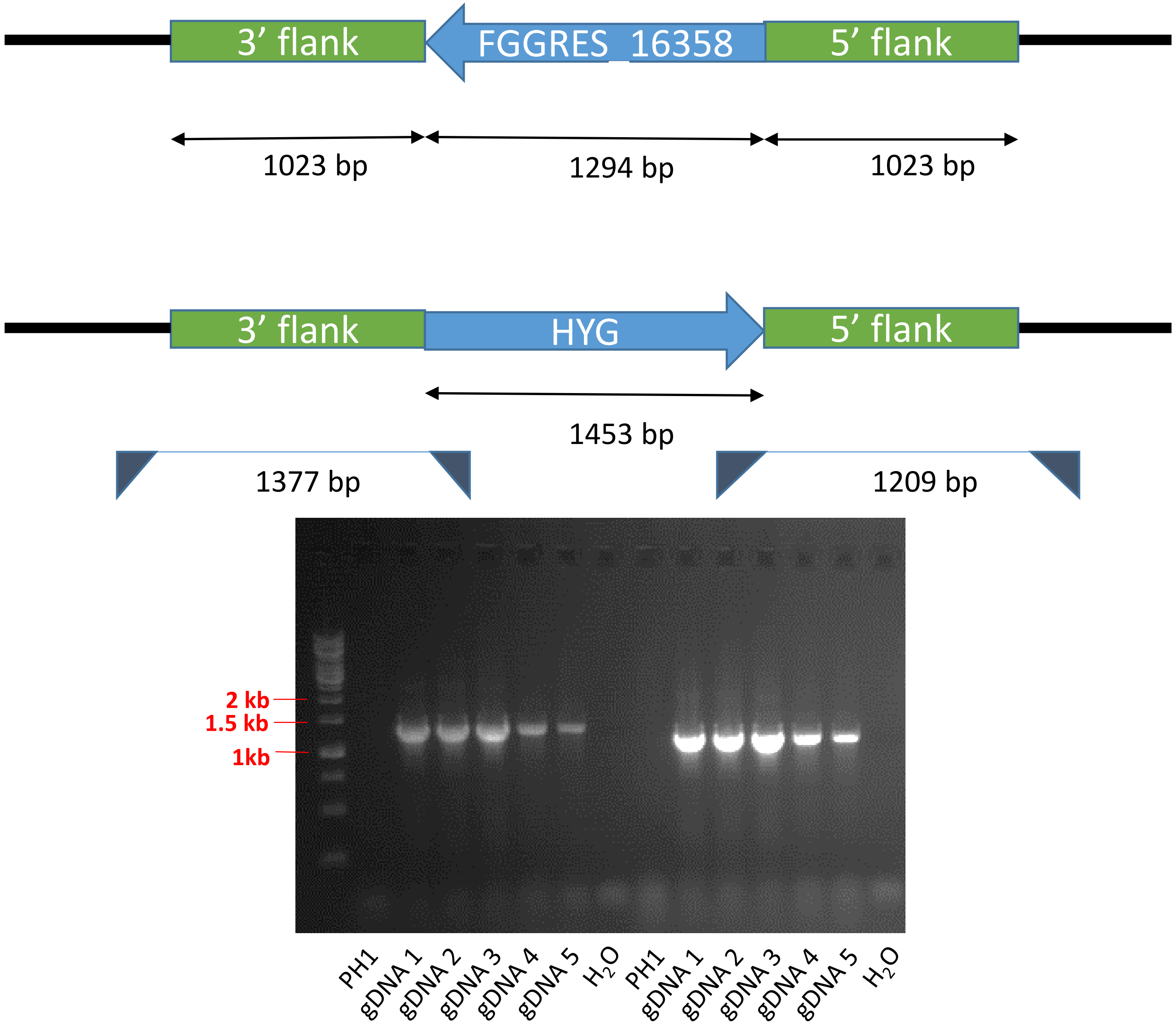

FHB symptoms on wheat 15 days post infection

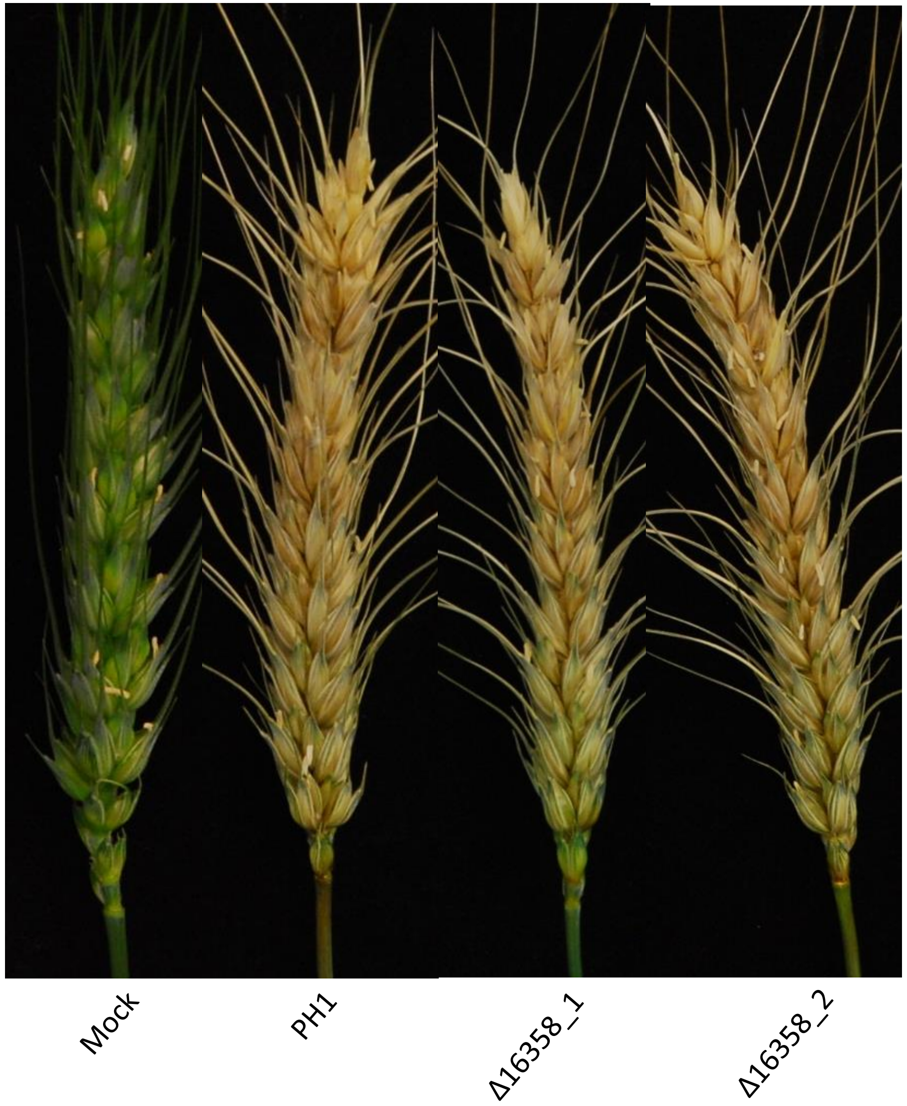

Class X: FGRRES_ 16437

Split marker mediated gene replacement

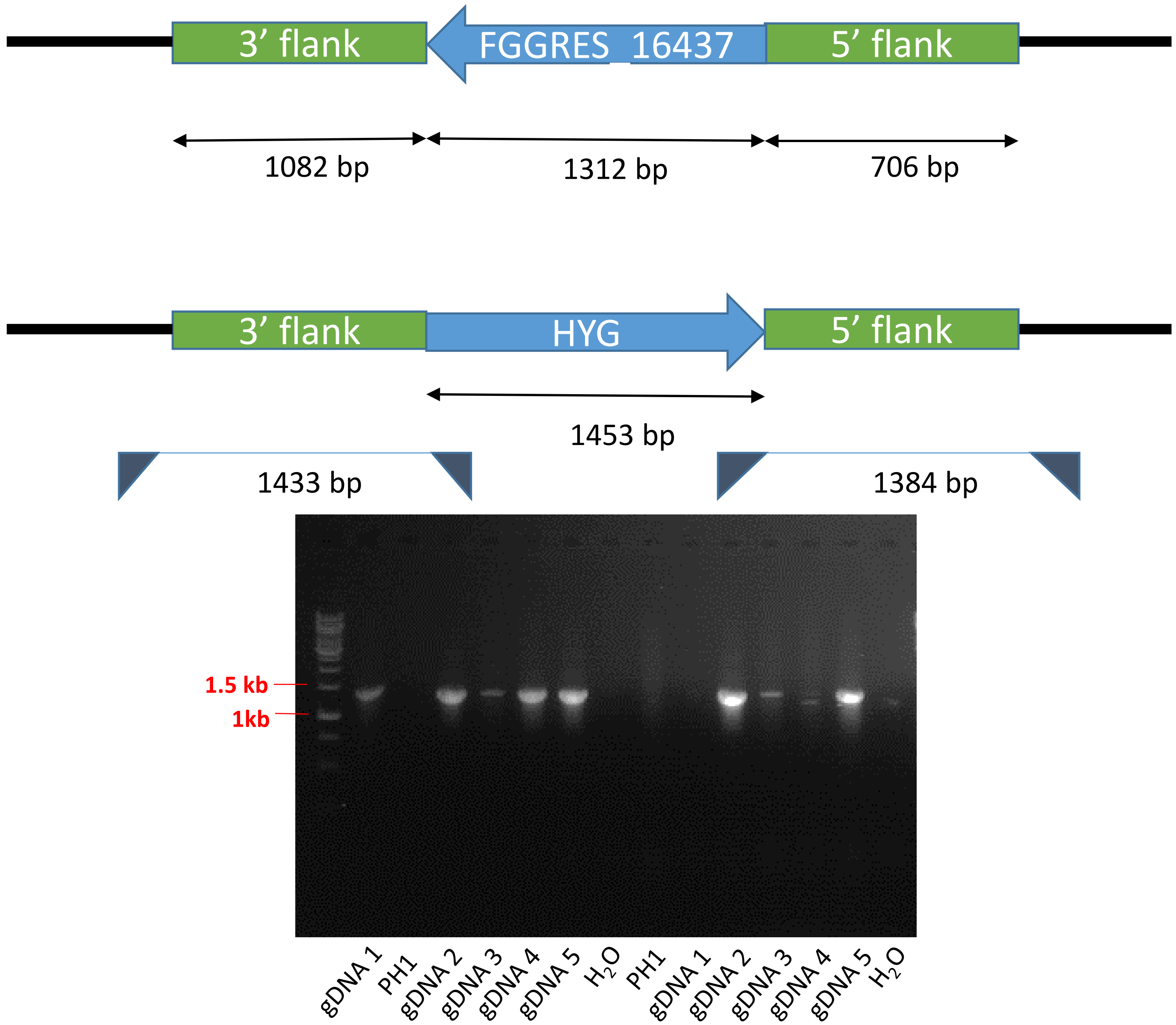

FHB symptoms on wheat 15 days post infection

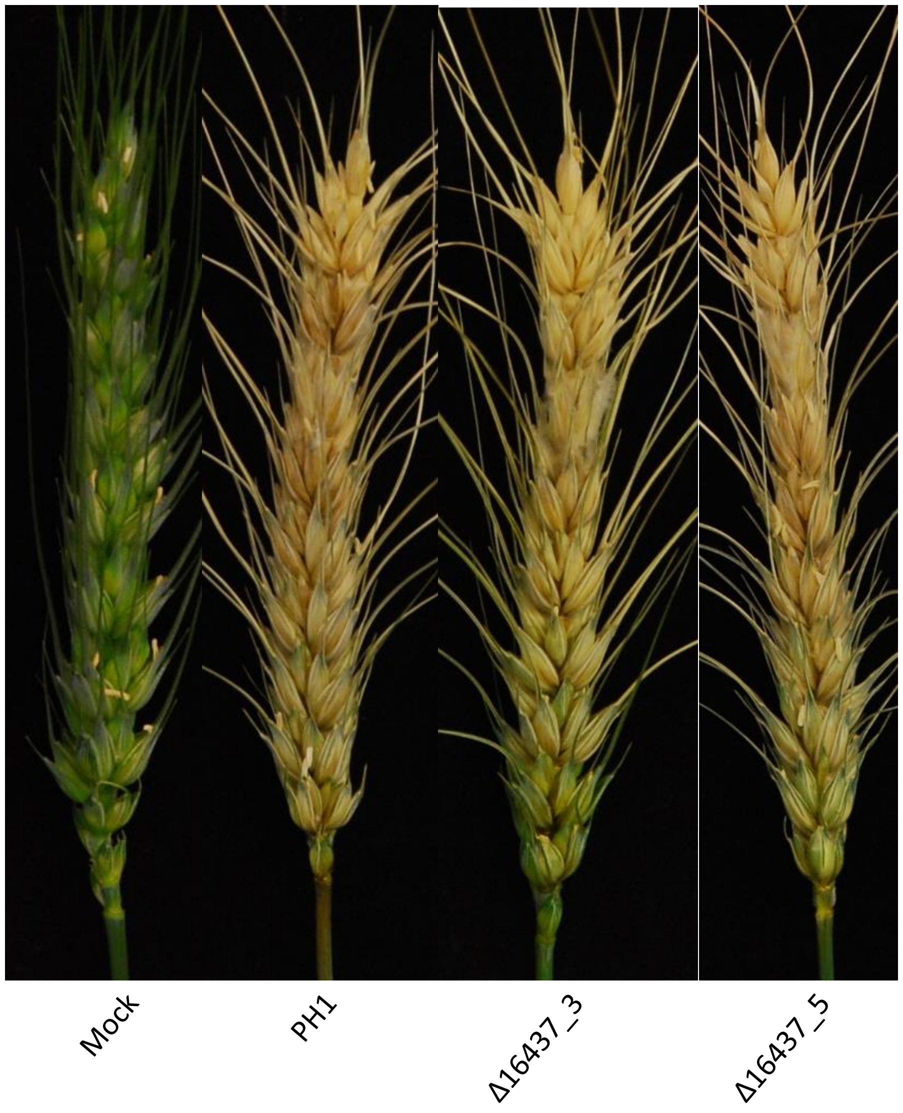

Class X: FGRRES_ 16536

Split marker mediated gene replacement

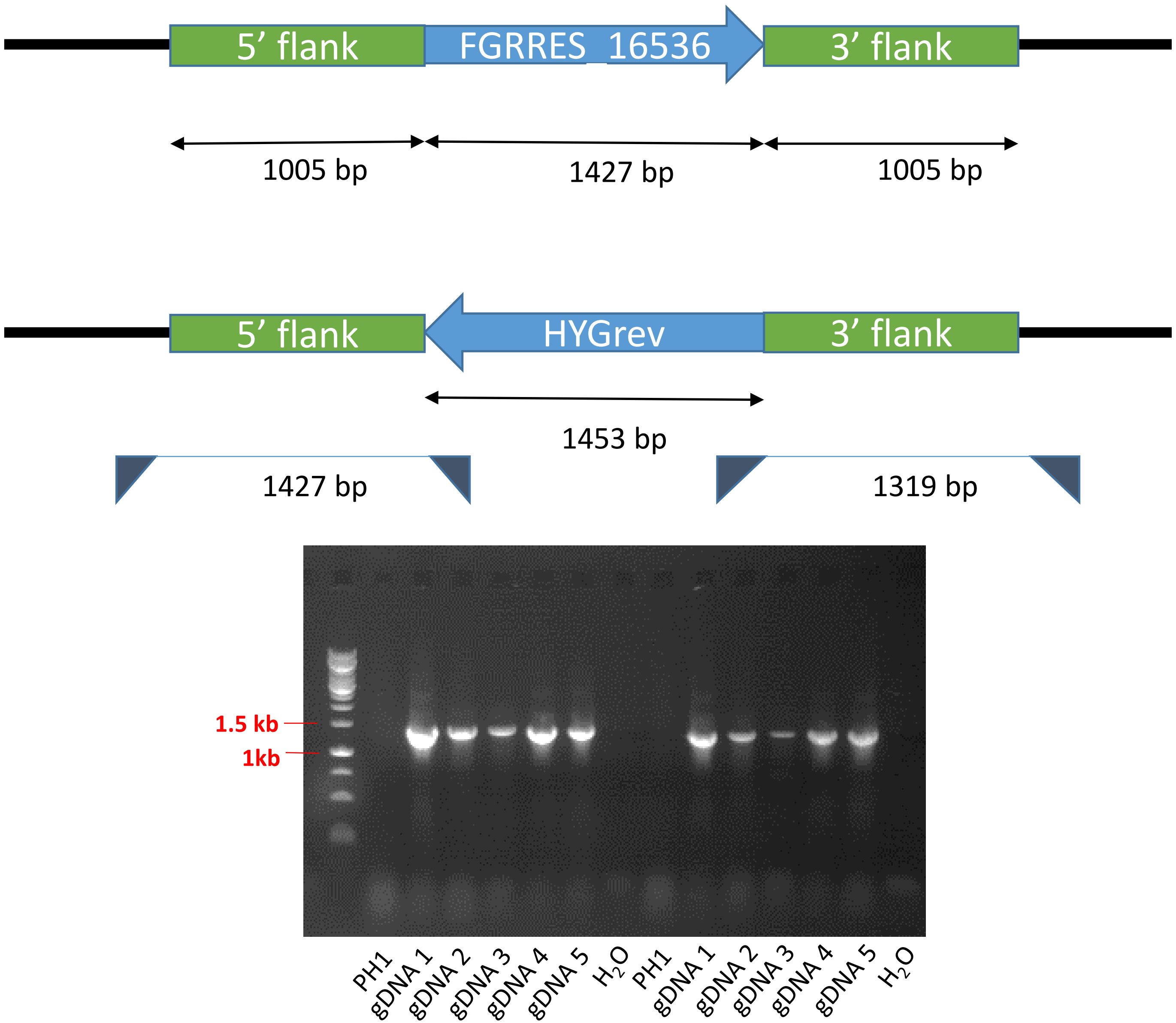

FHB symptoms on wheat 15 days post infection

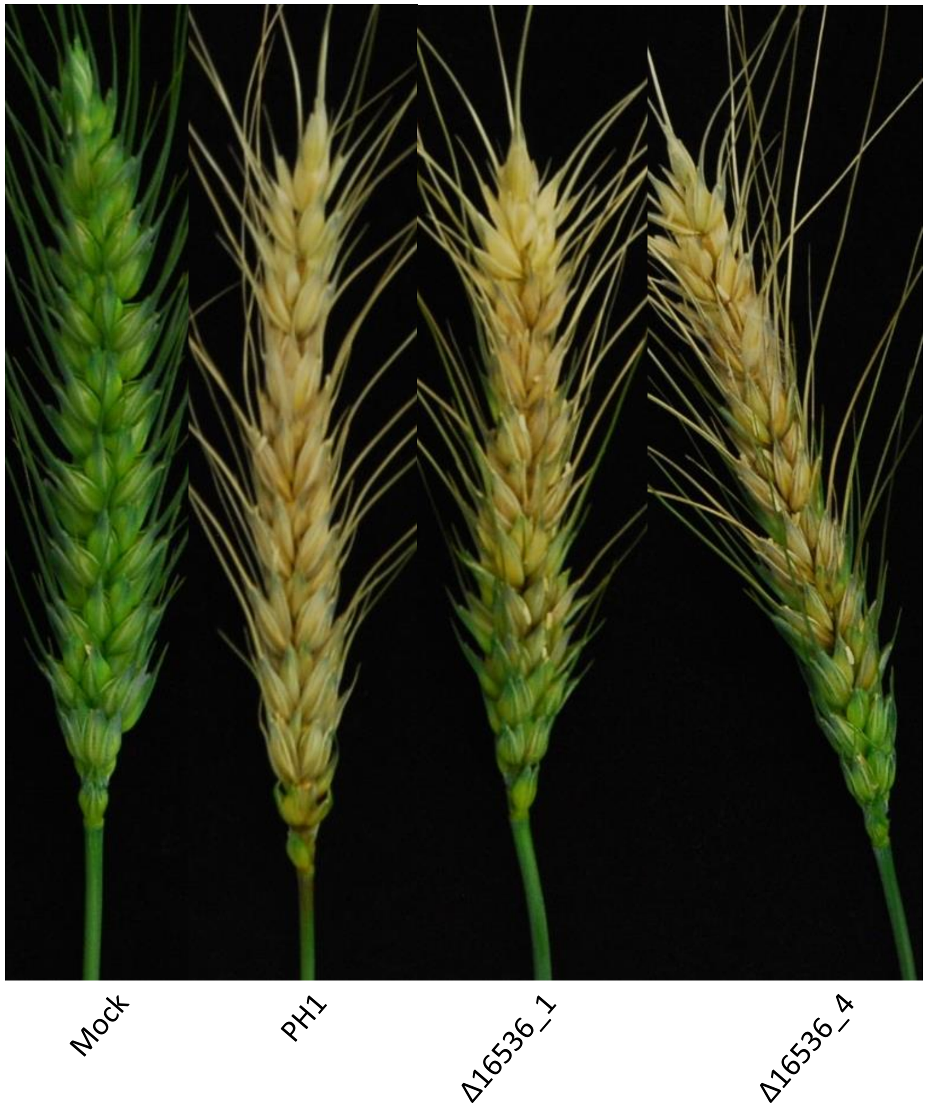

Class X: FGRRES_ 16853

Split marker mediated gene replacement

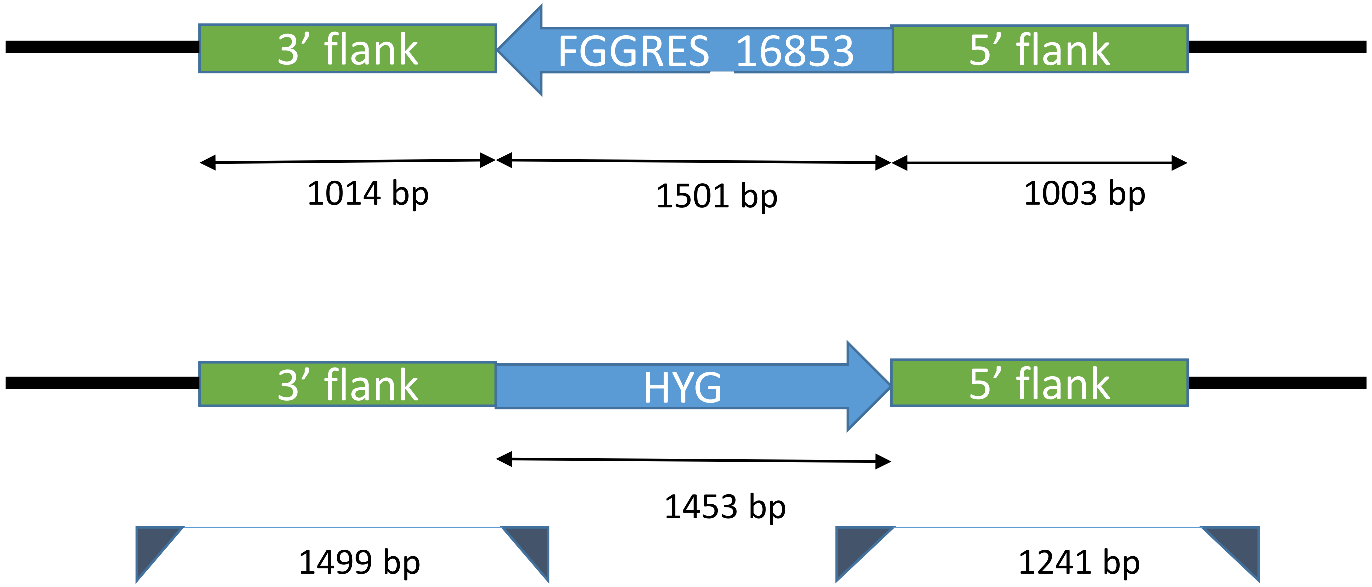

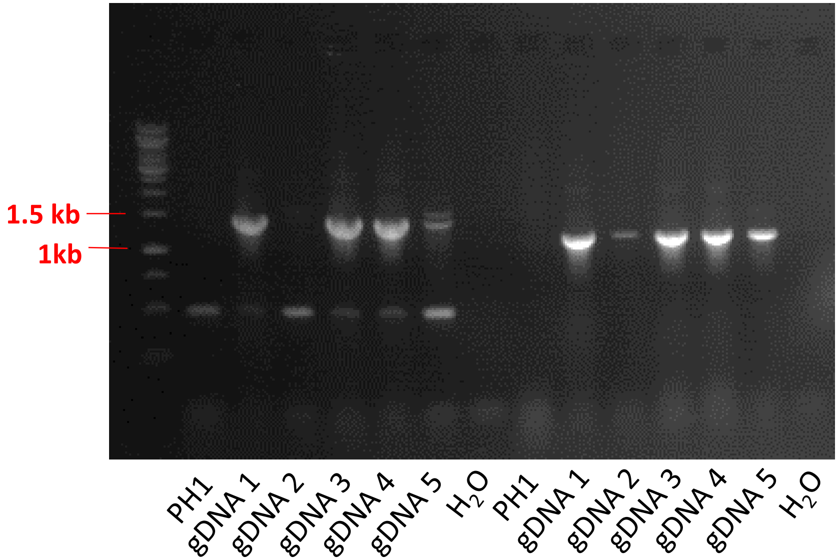

FHB symptoms on wheat 15 days post infection

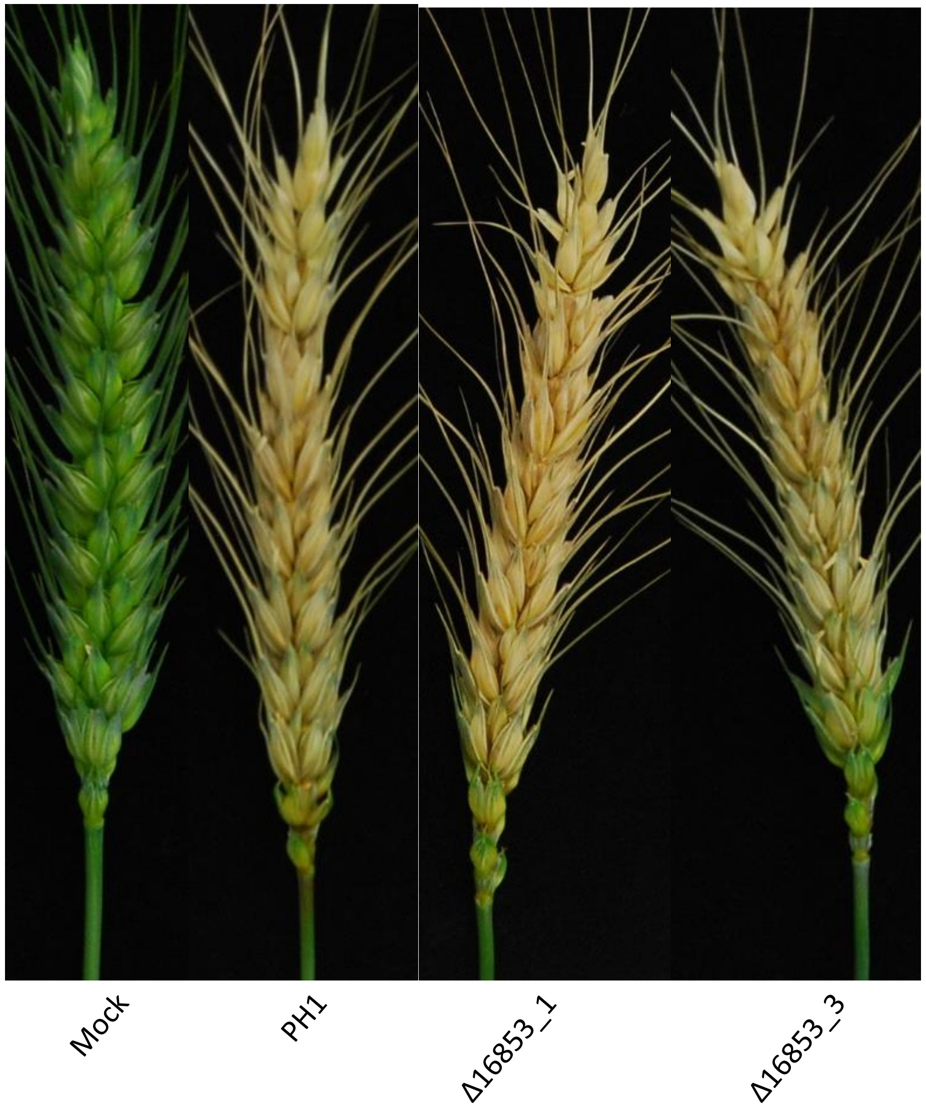

Class X: FGRRES_ 17567

Split marker mediated gene replacement

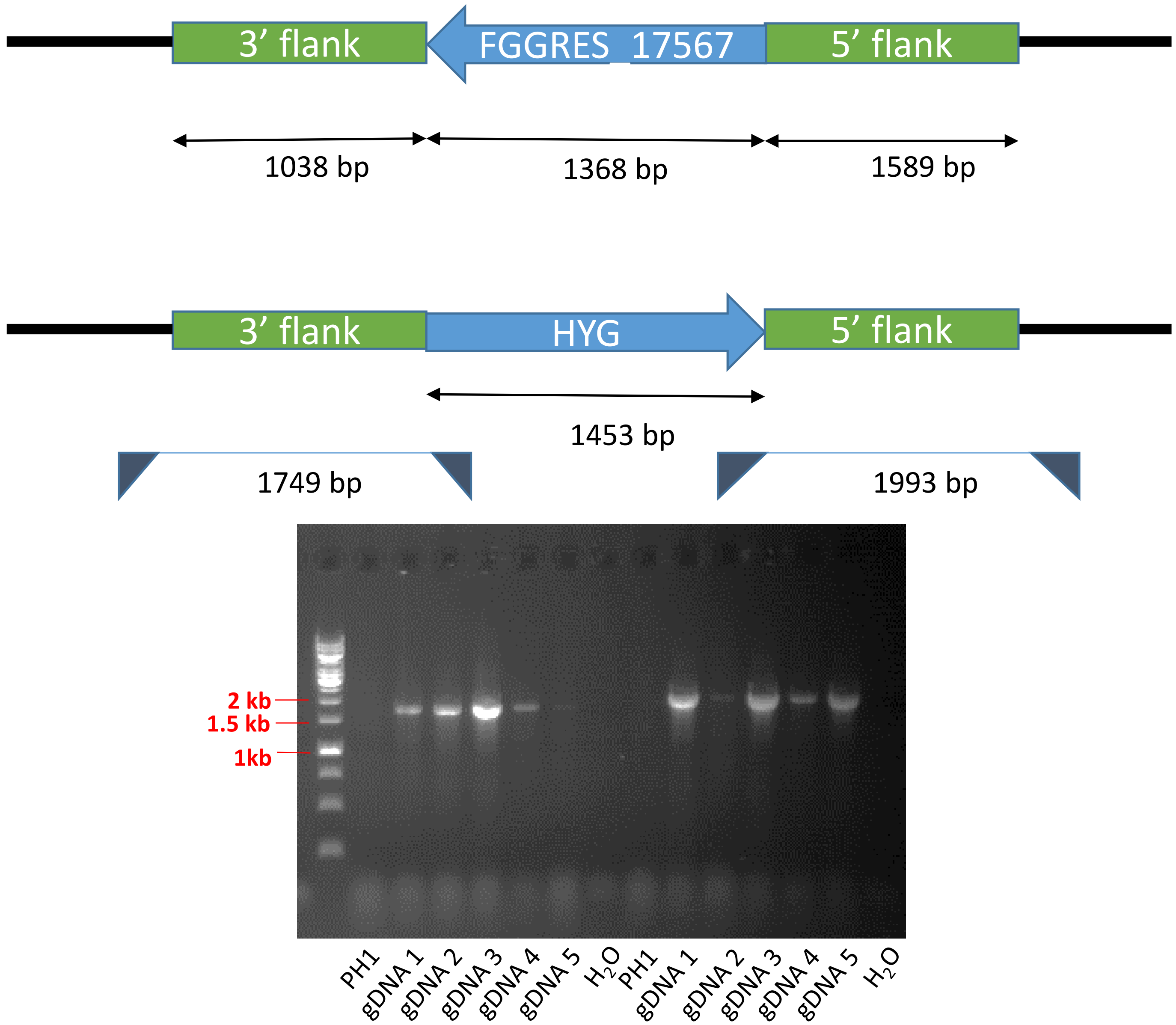

FHB symptoms on wheat 15 days post infection

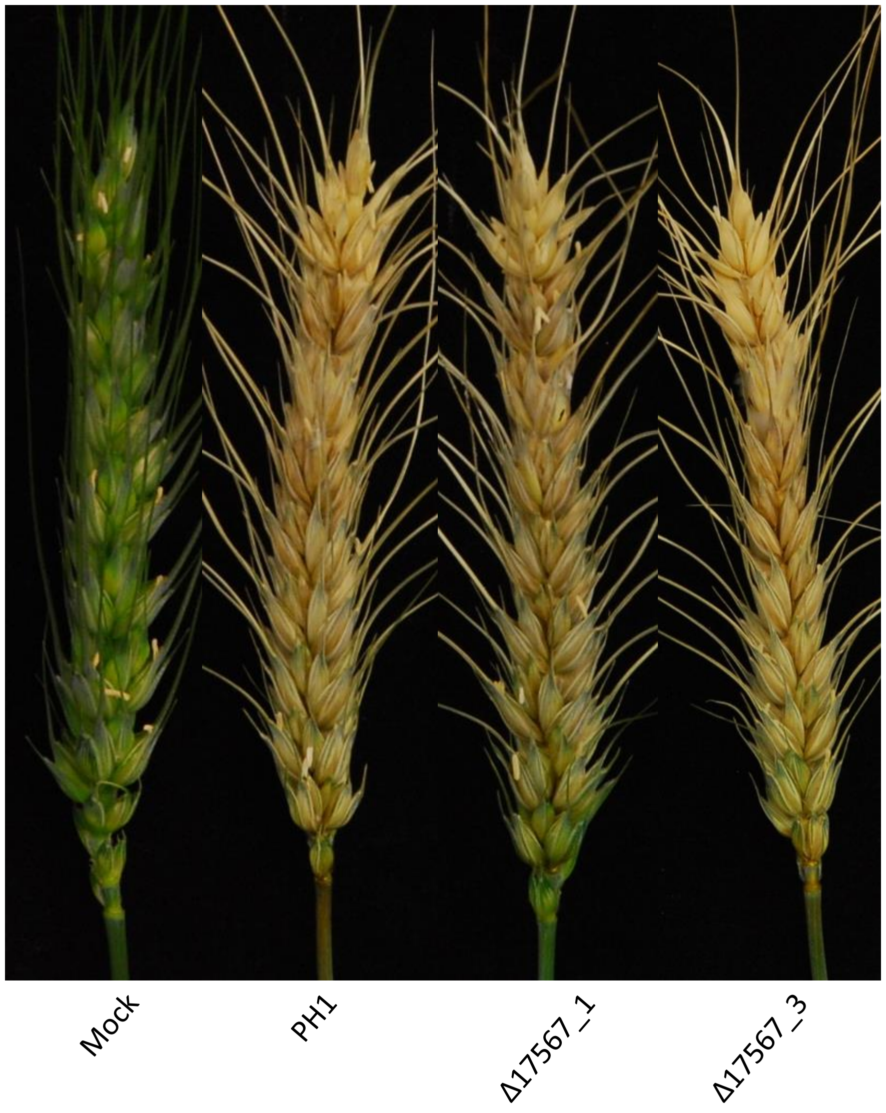

Class X+CFEM: FGRRES_ 02155

Split marker mediated gene replacement

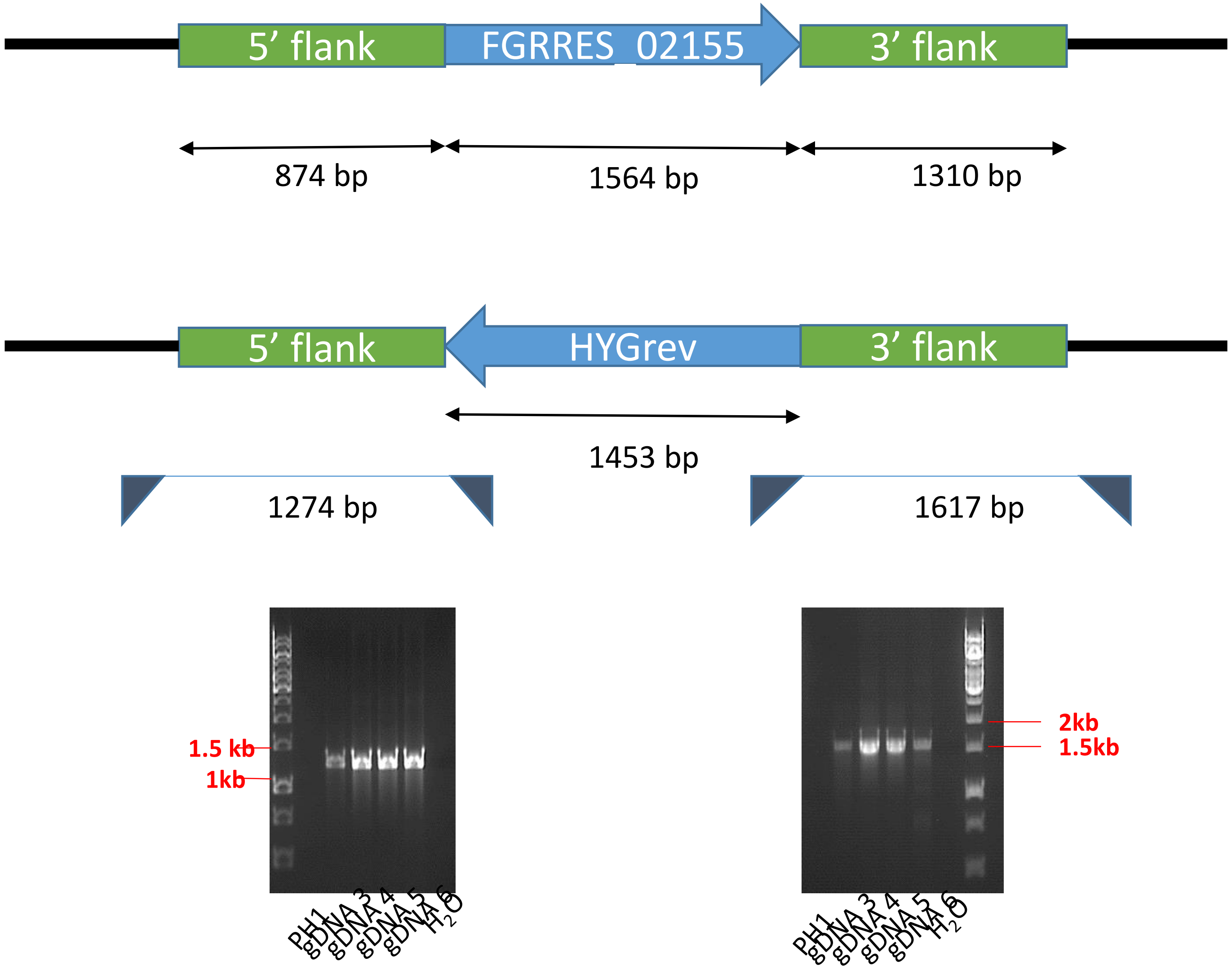

FHB symptoms on wheat 15 days post infection

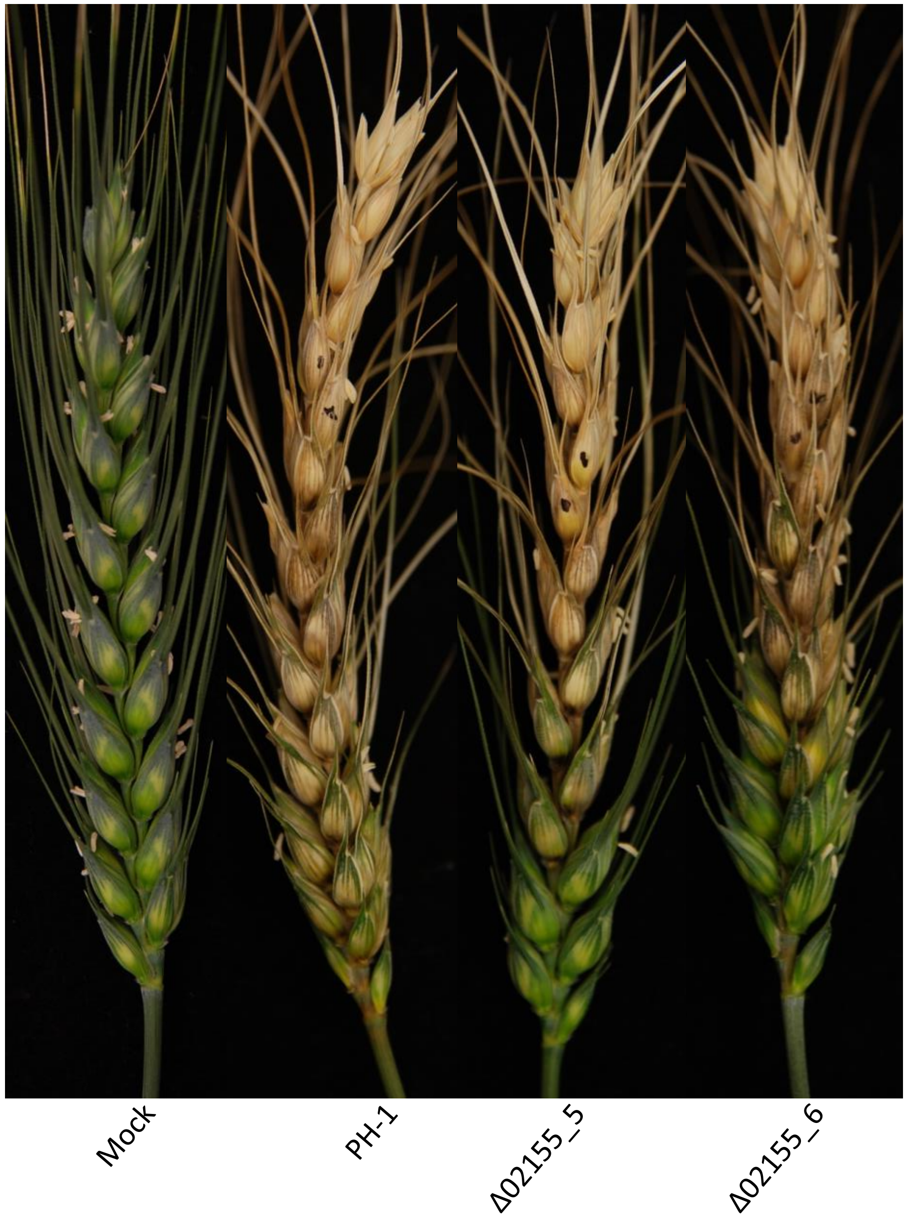

Class X+CFEM: FGRRES_ 03897

Split marker mediated gene replacement

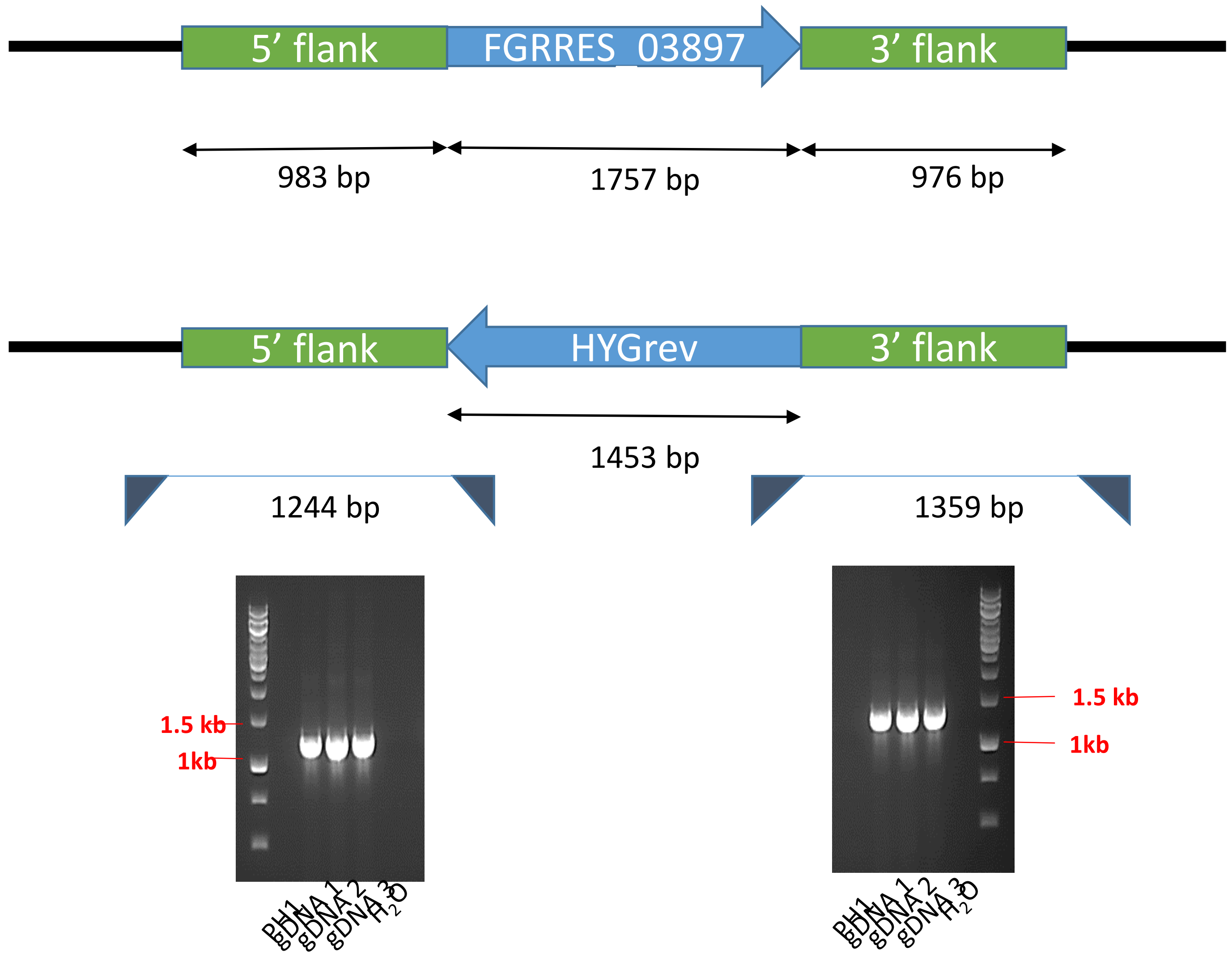

FHB symptoms on wheat 15 days post infection

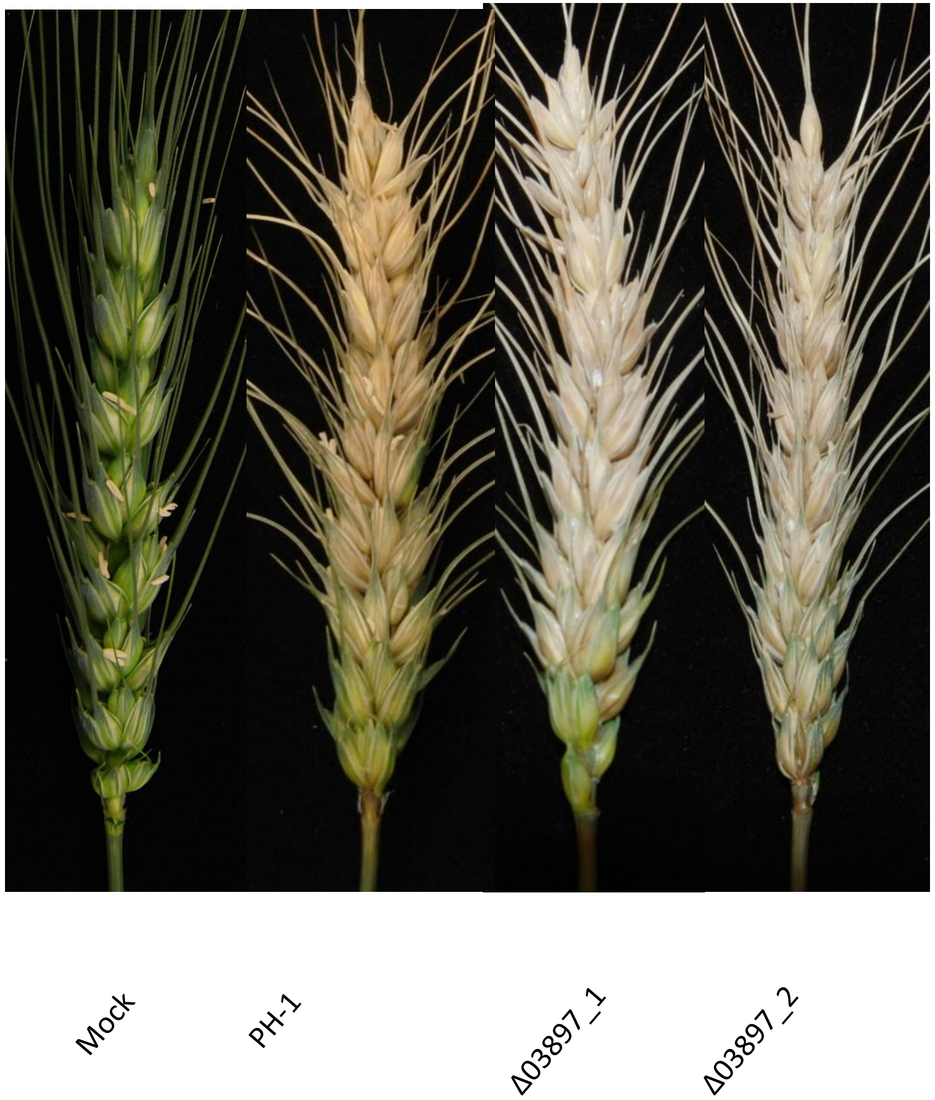

Class X+CFEM: FGRRES_ 04529

Split marker mediated gene replacement

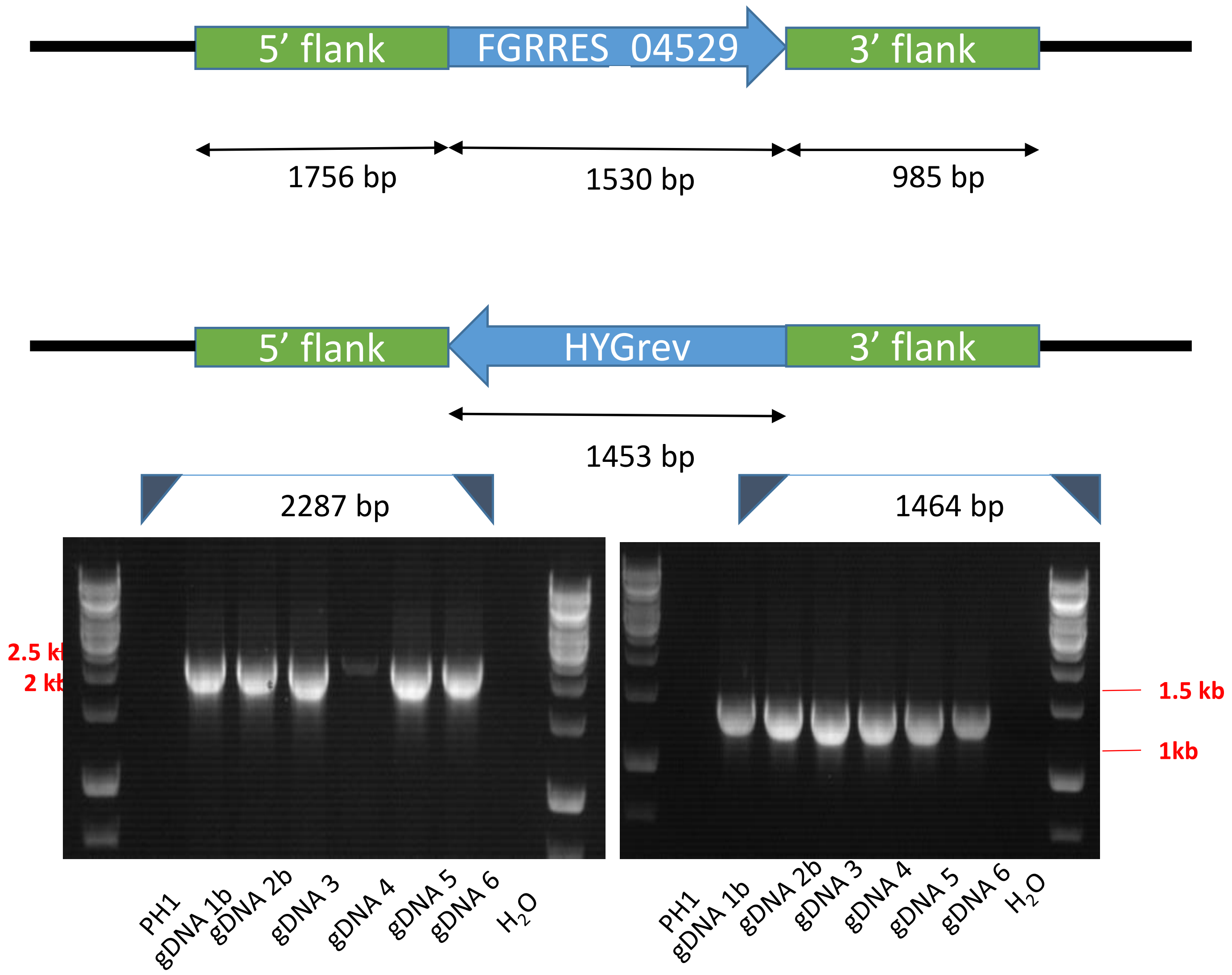

FHB symptoms on wheat 15 days post infection

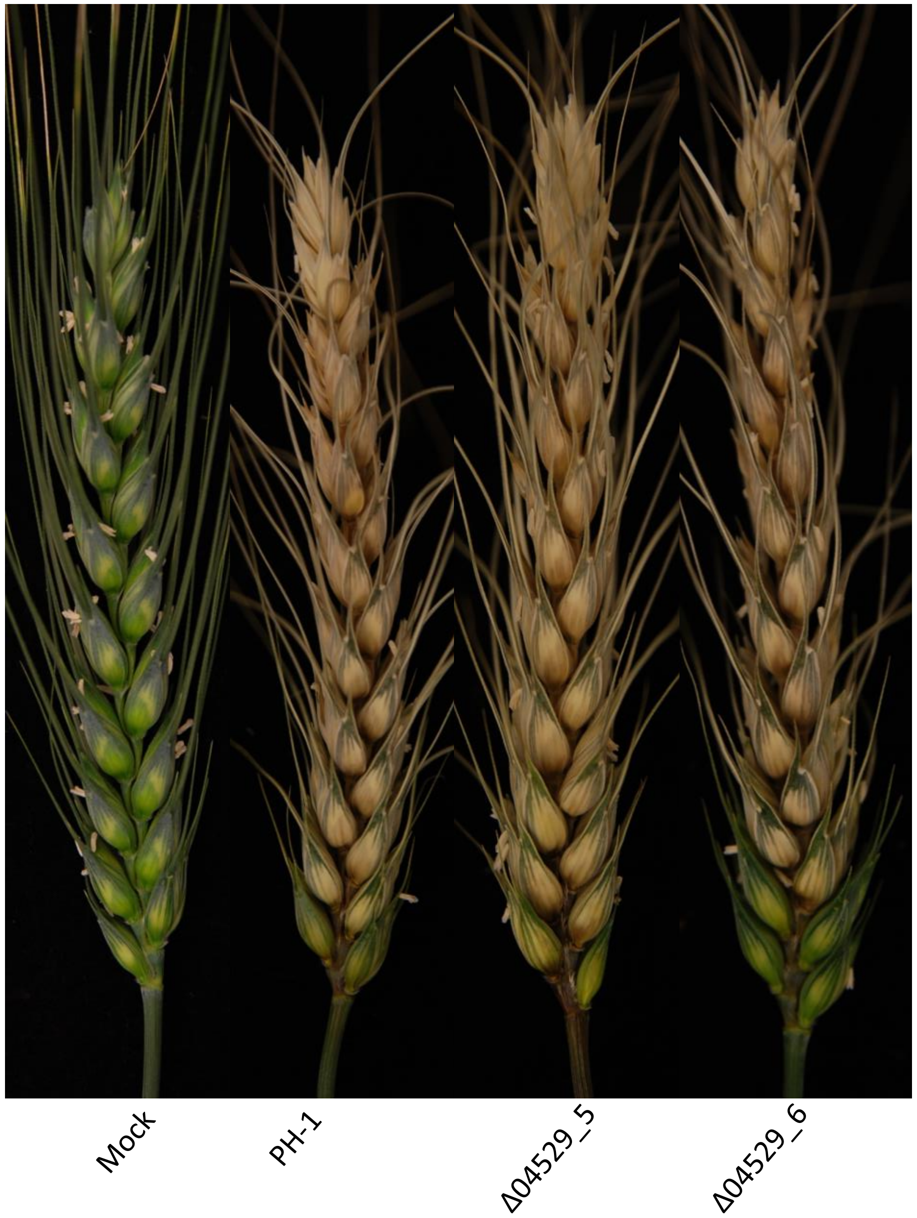

Class X+CFEM: FGRRES_ 05821

Split marker mediated gene replacement

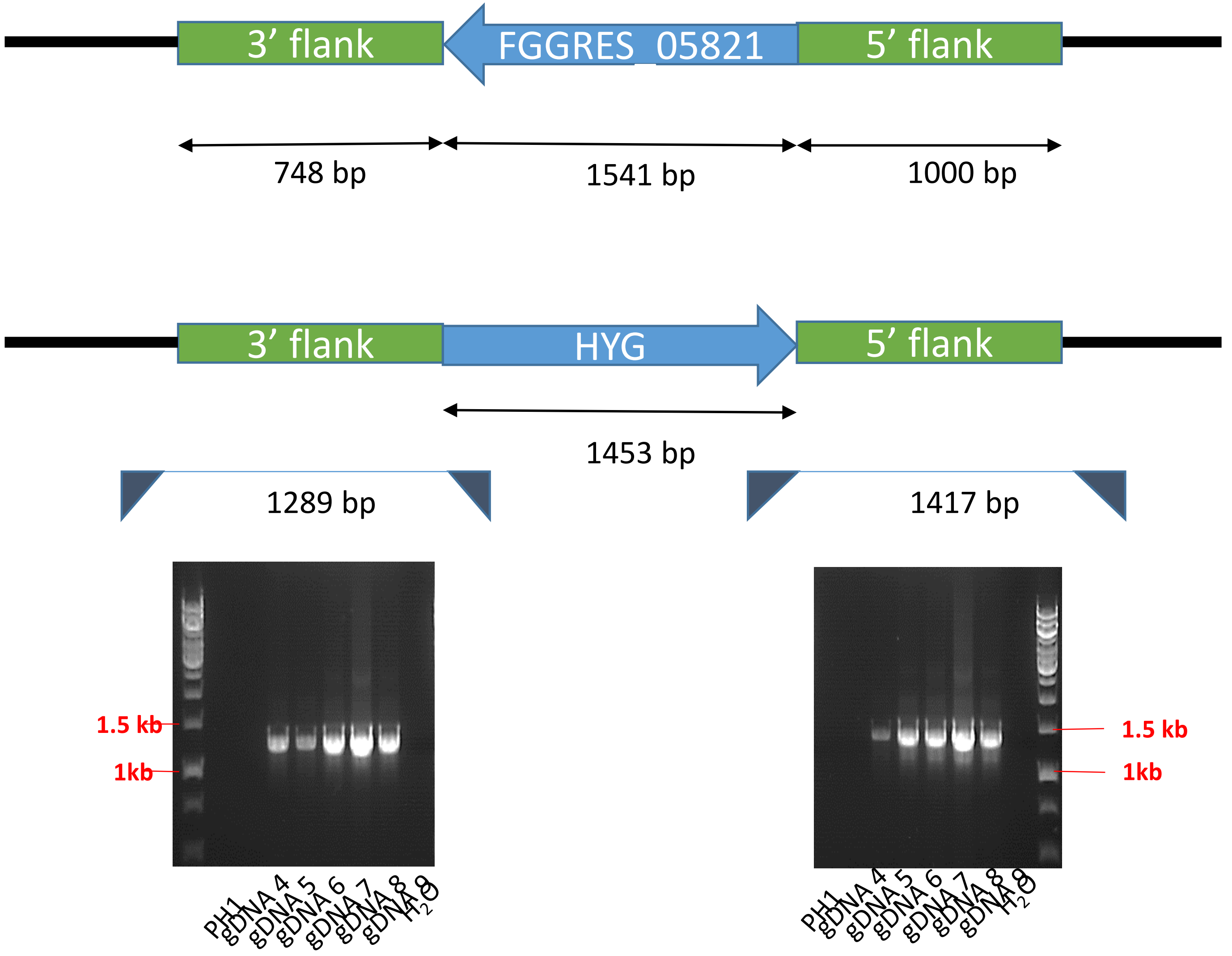

FHB symptoms on wheat 15 days post infection

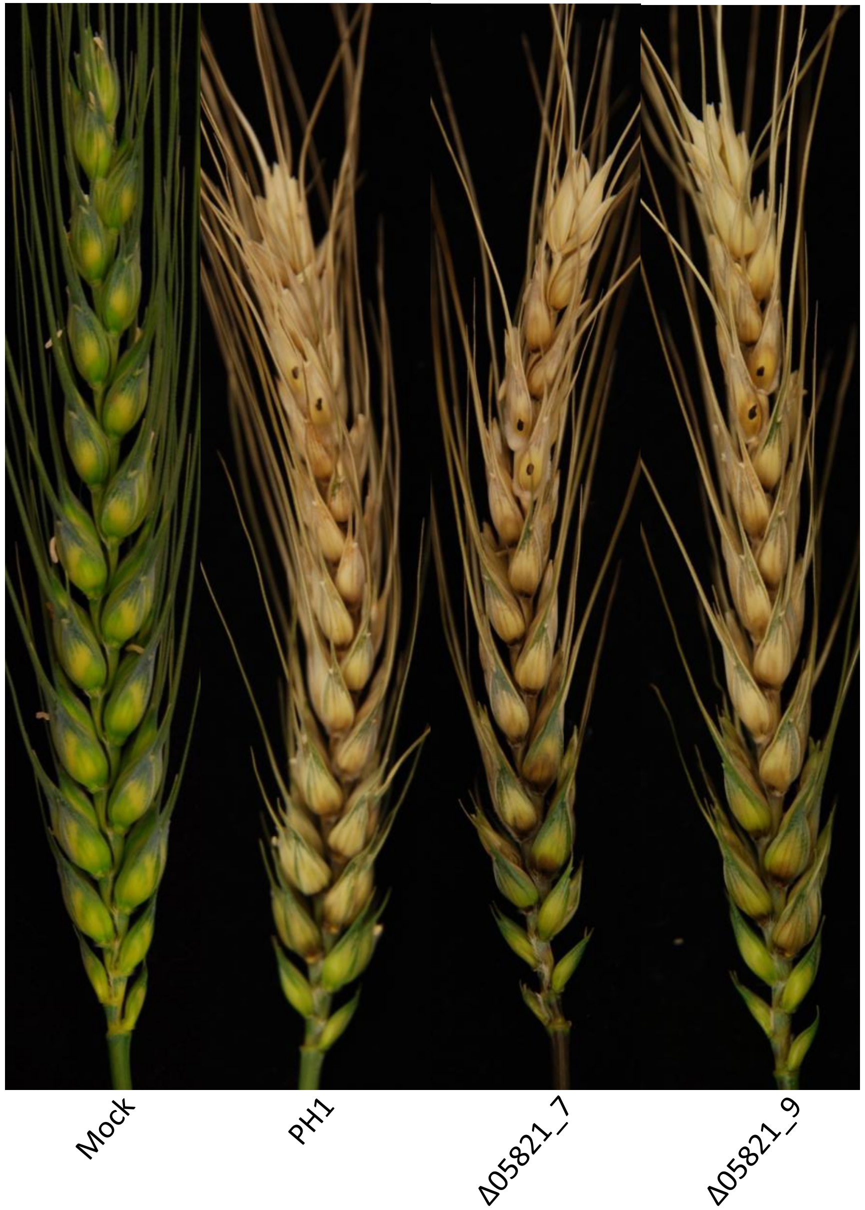

Class X+CFEM: FGRRES_ 07839

Split marker mediated gene replacement

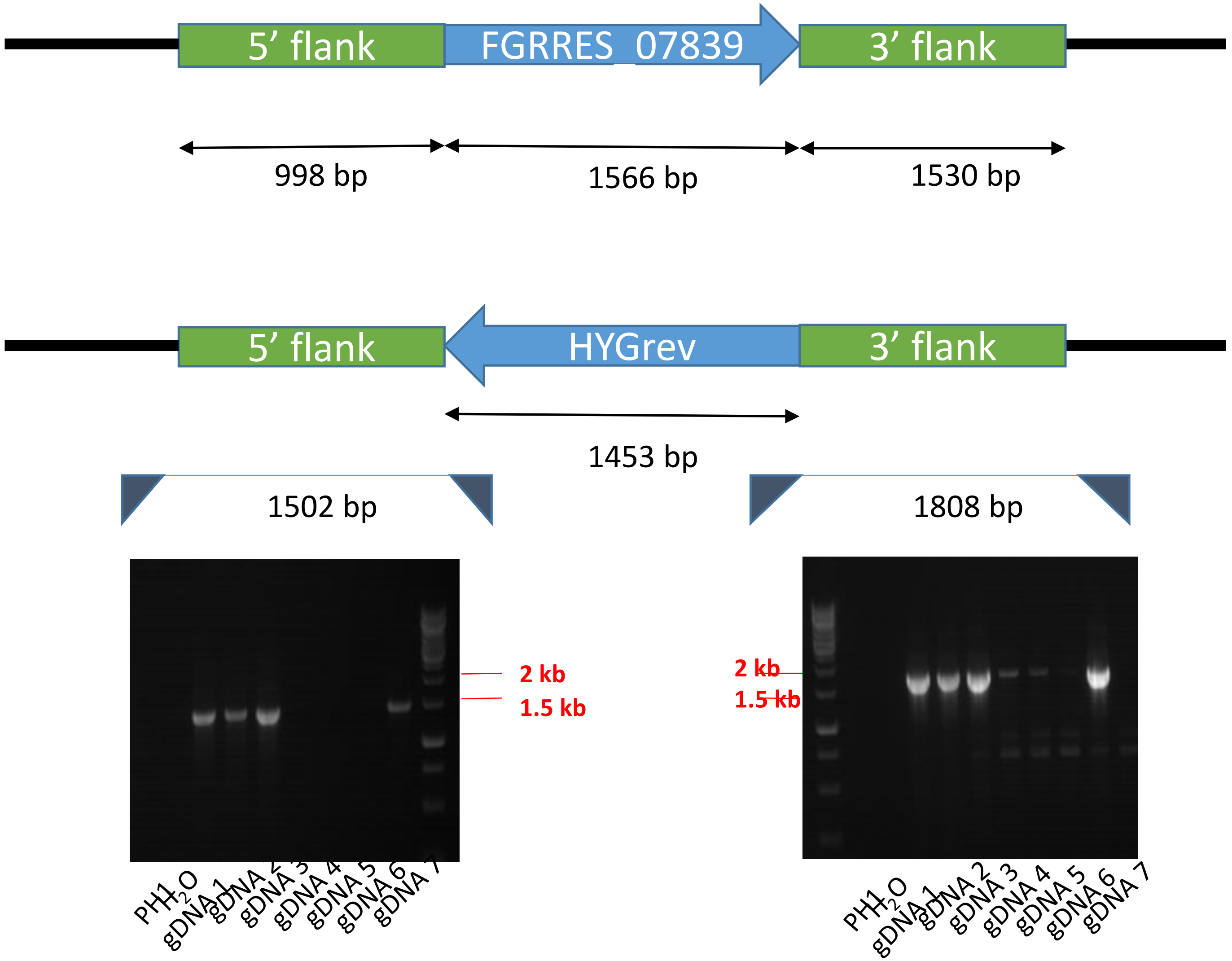

FHB symptoms on wheat 15 days post infection

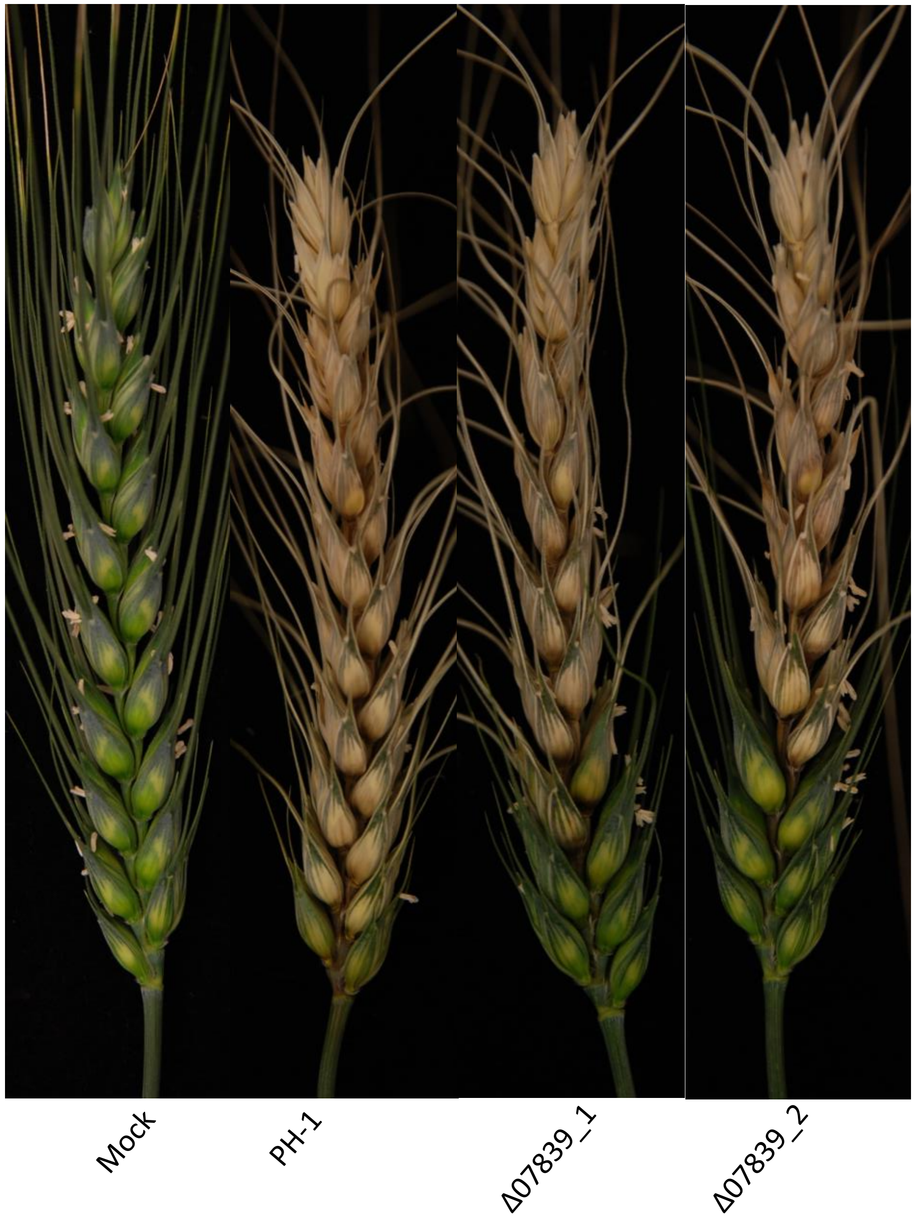

Class X+CFEM: FGRRES_ 15975

Split marker mediated gene replacement

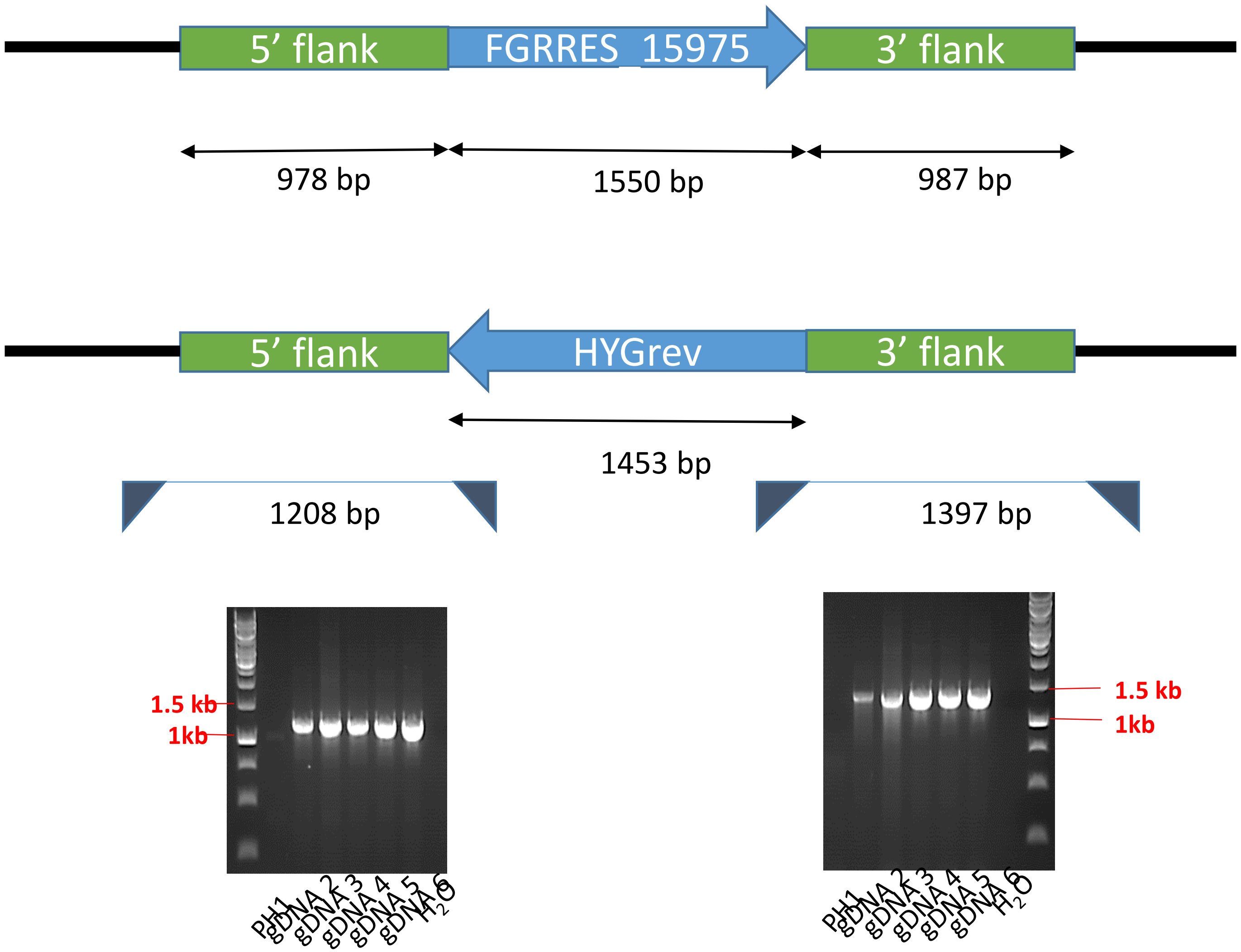

FHB symptoms on wheat 15 days post infection

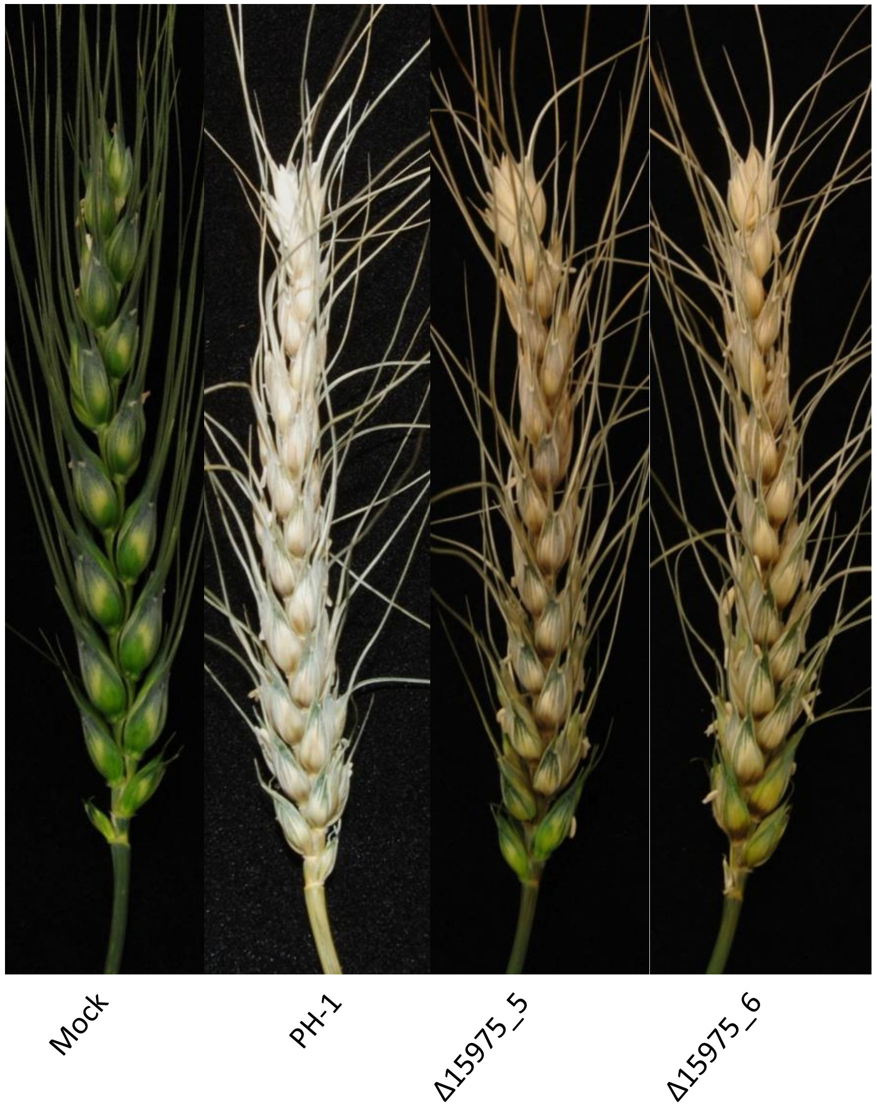

Class X+CFEM: FGRRES_16221

Split marker mediated gene replacement

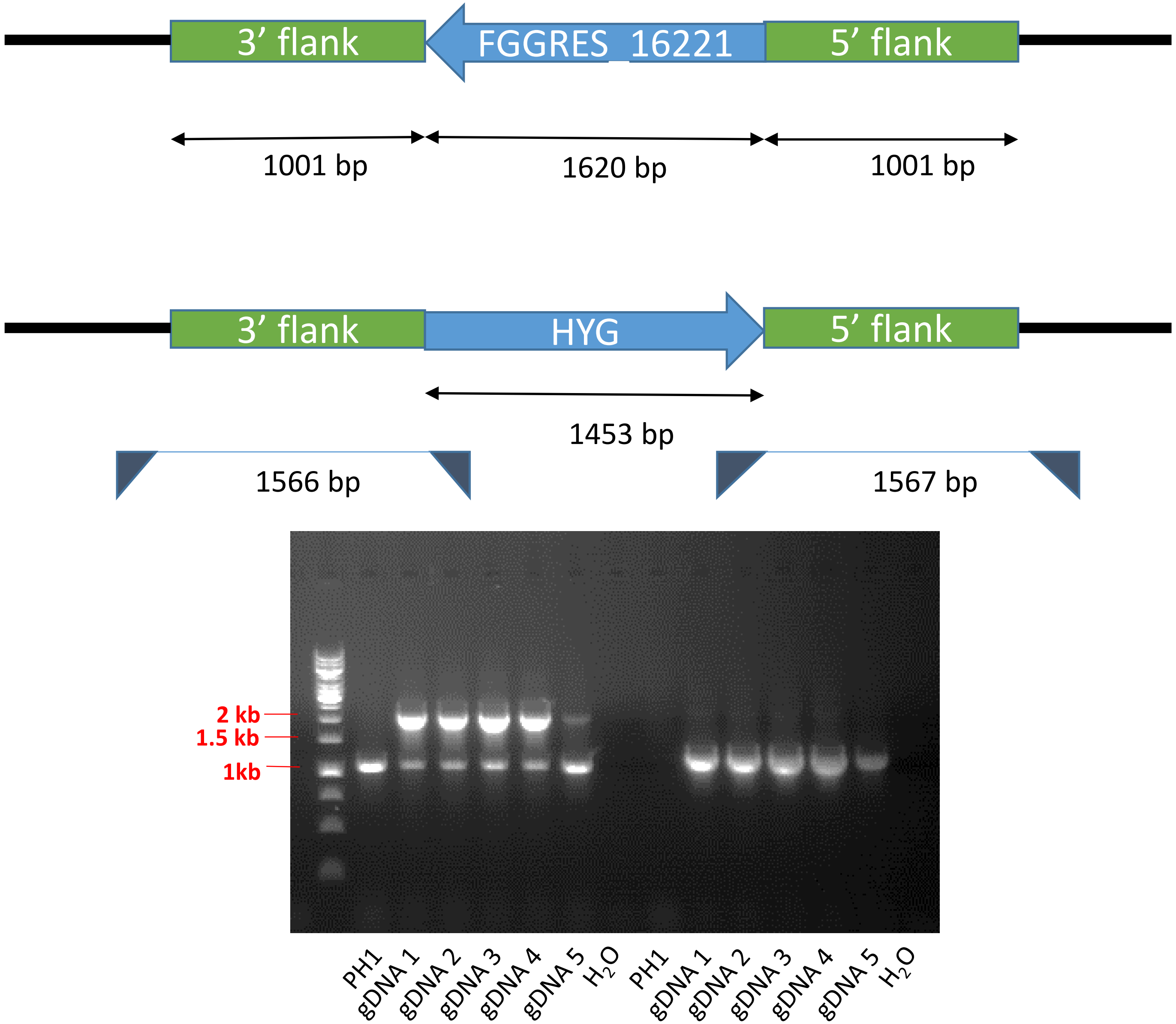

FHB symptoms on wheat 15 days post infection

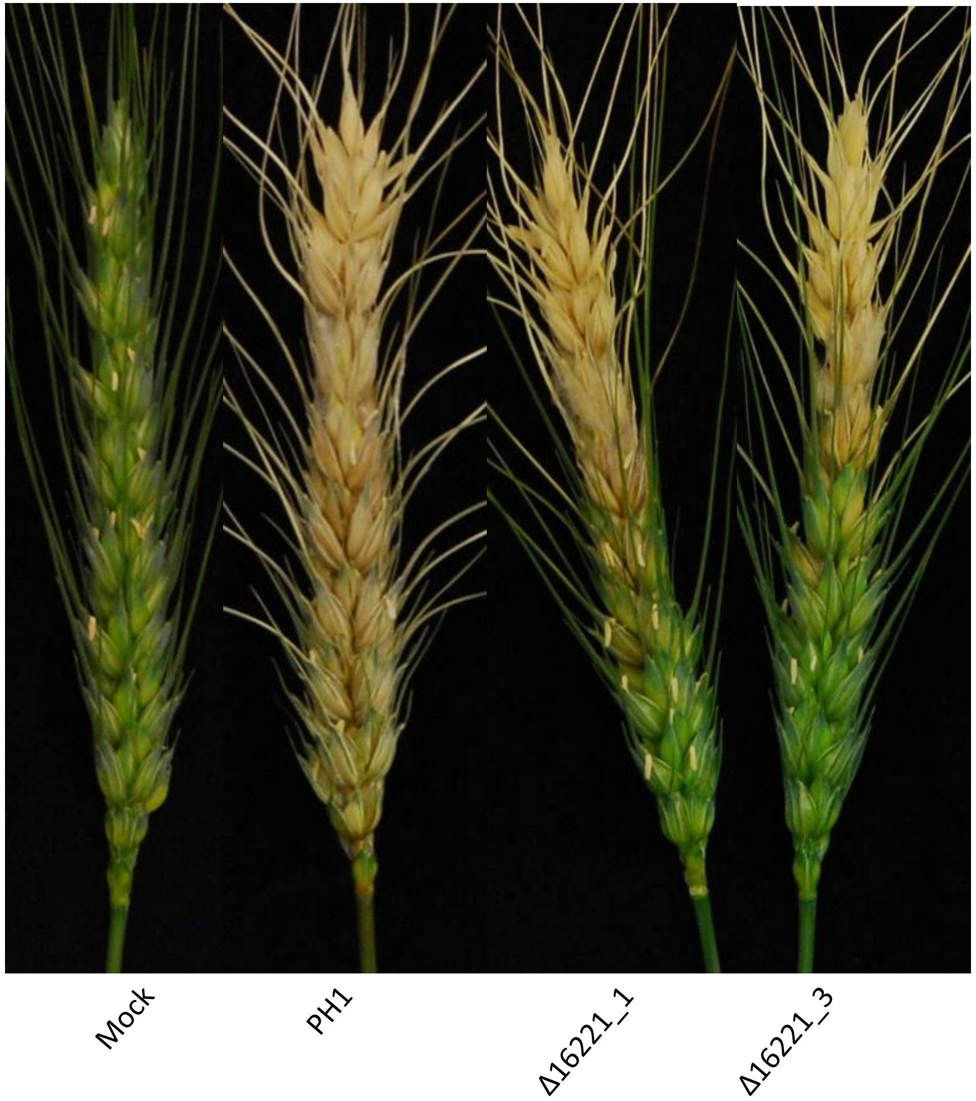

Class X+CFEM: FGRRES_16221 truncations

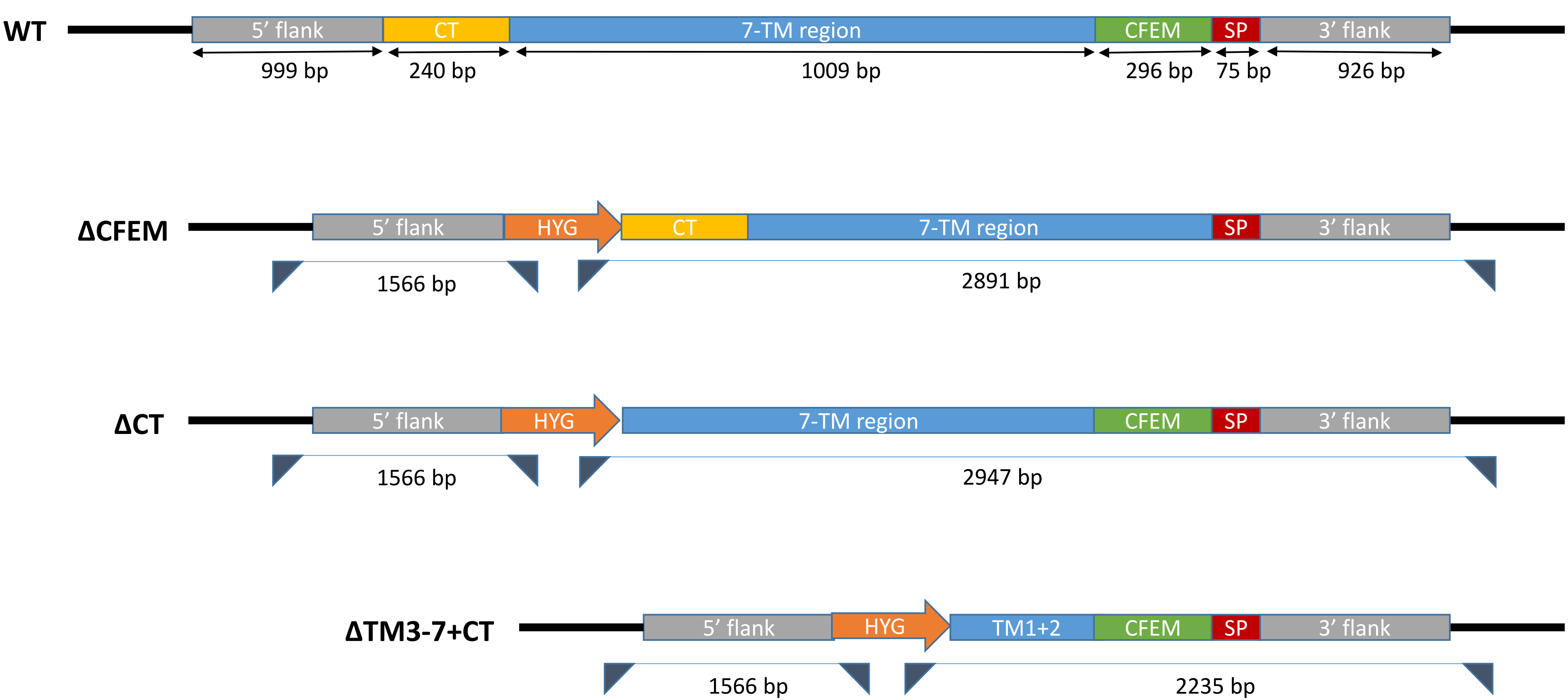

Class X+CFEM: FGRRES_16221 truncations

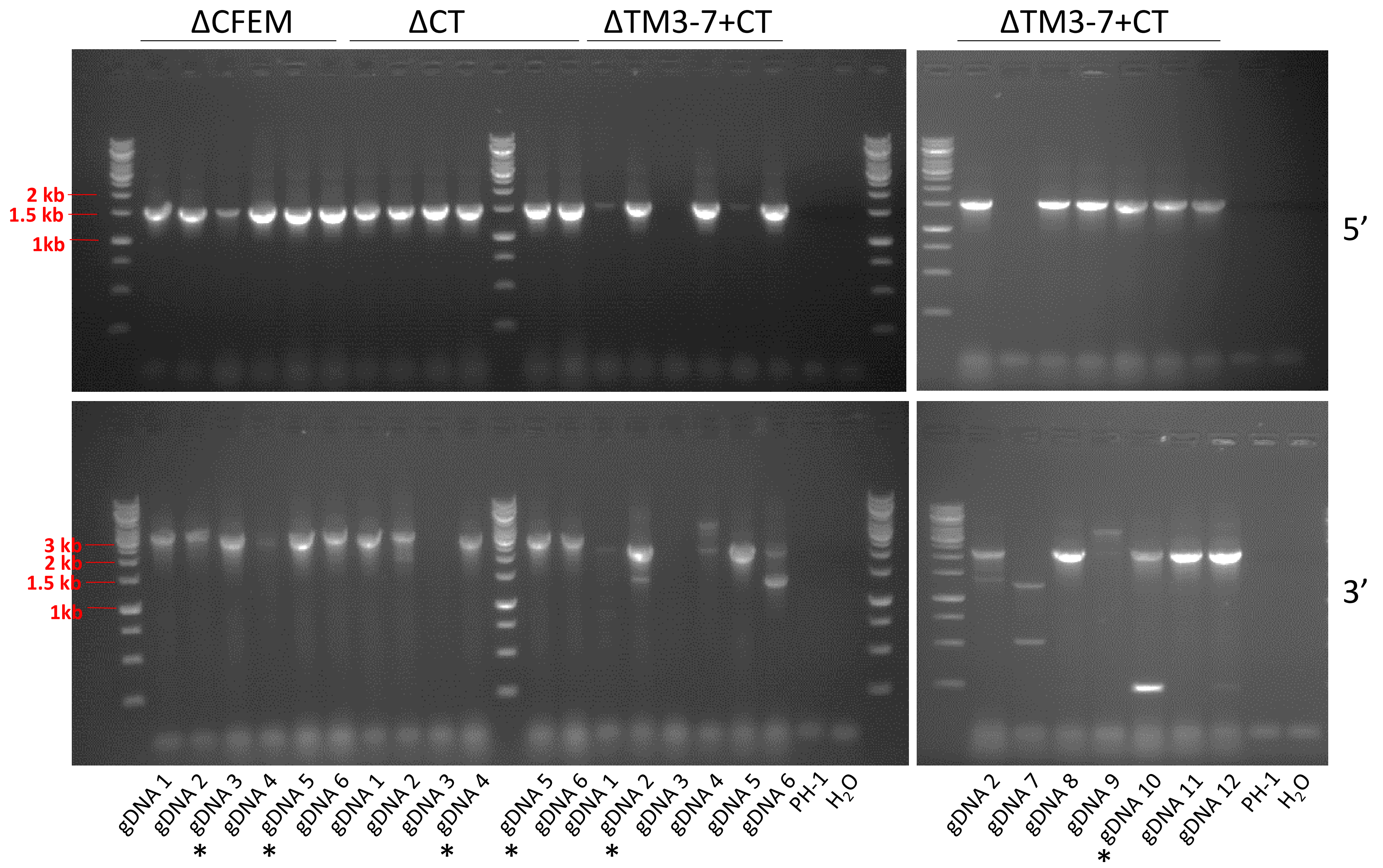

Selected transformants: ΔCFEM_1/2 ΔCT_1/2 ΔTM3-7+CT_1/2

Class X+CFEM: FGRRES_16221 complementation

Split marker mediated gene replacement

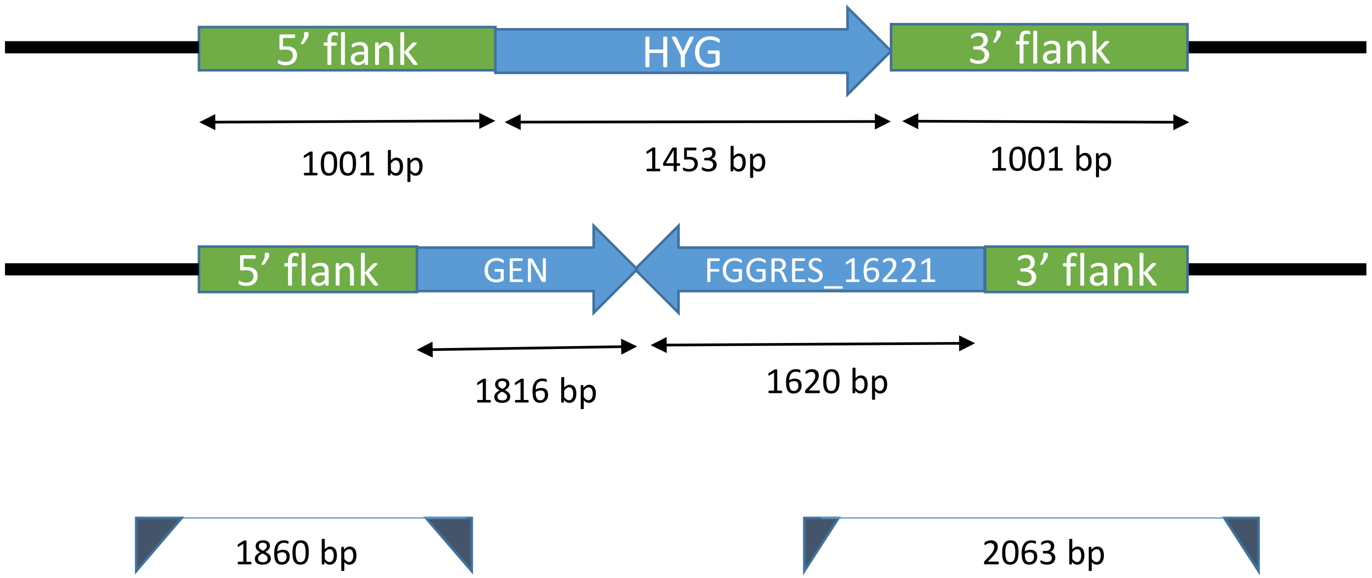

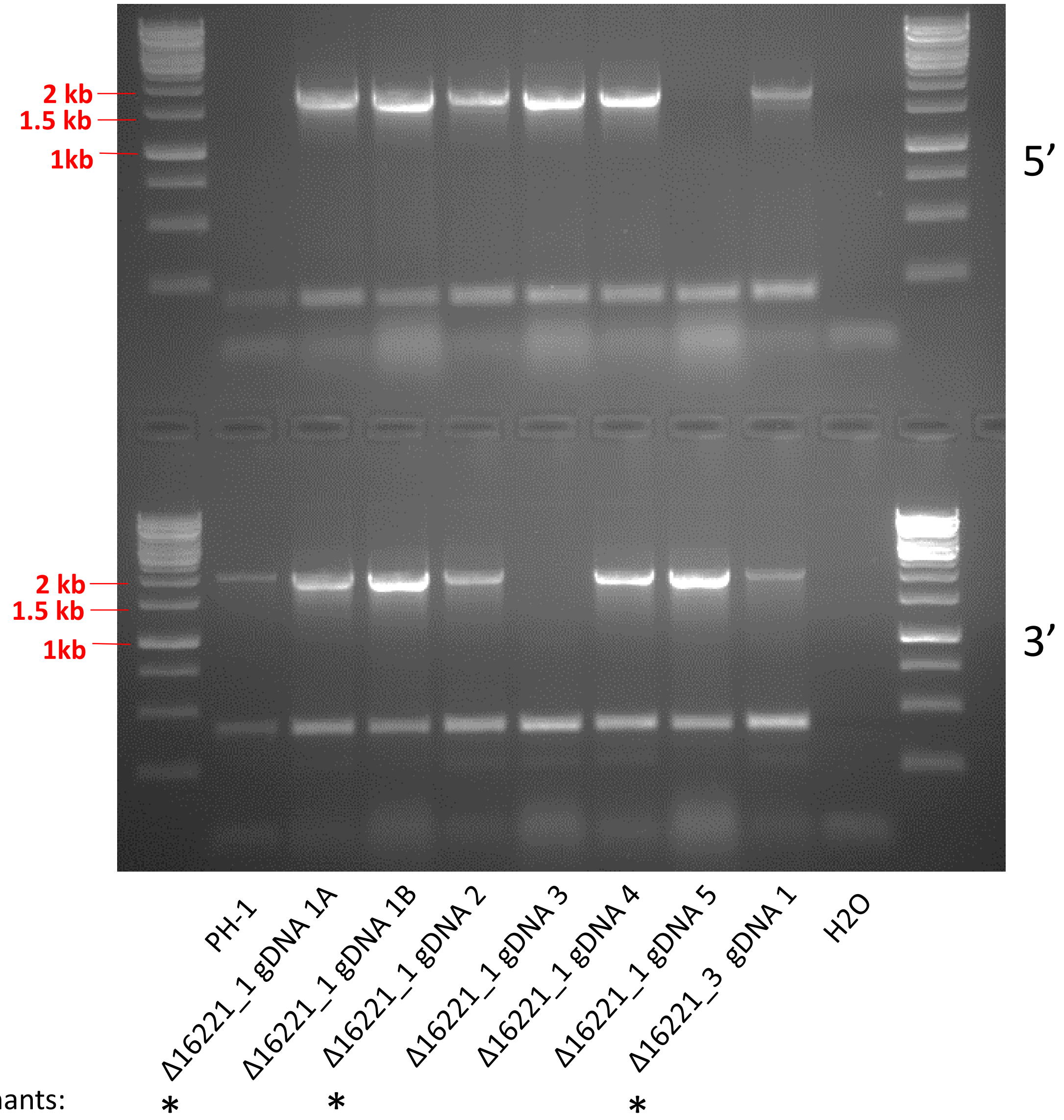

Selected transformants:
